# Genetic diversity and population structure of early-maturing yellow and orange maize inbred lines for improved breeding applications

**DOI:** 10.1371/journal.pone.0358751

**Published:** 2026-09-25

**Authors:** Neo Jeremiah Mahula, Idris Ishola Adejumobi, Julius Akinyemi Fagbayide, Baffour Badu-Apraku, John Derera

**Affiliations:** 1 Program of Plant Breeding, Pan African University Life and Earth Sciences Institute (including Health and Agriculture), PAULESI, Nigeria; 2 Maize Improvement Program, (MIP), International Institute of Tropical Agriculture (IITA), Ibadan, Nigeria; Sant Baba Bhag Singh University, INDIA

## Abstract

Early-maturing maize is vital for strengthening food security across the drought-prone agro-ecologies of Sub-Saharan Africa (SSA). Advancing genetic gain in these environments requires a clear understanding of genetic diversity and population structure patterns to support parental selection and future genomic-assisted breeding efforts. The study aimed at assessing genetic diversity and population structure of a panel of early-maturing yellow and orange kernel maize inbred lines, classify the inbred lines into putative heterotic groups, and identify a core set of elite, genetically divergent lines suitable for use in hybrid breeding and long-term genetic improvement. A panel of 376 elite early-maturing yellow and orange maize inbred lines from four source populations were genotyped using the DArTag SNP markers. After quality filtering, 1,954 high-quality SNP markers retained exhibited moderate diversity with an average polymorphic information content (PIC) of 0.38 and minor allele frequency (MAF) of 0.28. Linkage disequilibrium analysis showed a mean r^2^ of 0.046, with rapid LD decay across the genome, indicating substantial historical recombination and suggesting potential utility of the panel for future association studies. The Admixture analysis and discriminant analysis of principal components (DAPC) consistently resolved the panel into two major subpopulations with overlapping membership. Phylogenetic clustering also resolved the panel into two broad molecular clusters corresponding to putative heterotic groups. Analysis of molecular variance (AMOVA) revealed that most genetic variation occurred within groups (86%), with moderate differentiation among groups (14%). A core set representing 20% of the collection was identified using an allelic-richness–based greedy algorithm, offering valuable insights to accelerate breakthrough hybrid development for SSA farming systems. The study provides a comprehensive genomic characterization of early-maturing maize germplasm, providing genomic insights that may support future hybrid development and germplasm management efforts for SSA maize breeding programs.

## Introduction

Maize (*Zea mays* L.) is a major cereal crop that provides food, feed, and industrial products for millions of people in sub-Saharan Africa (SSA) [1]. Its starchy endosperm makes it a key energy source, and the crop plays a central role in food and nutritional security across the region [2]. However, the predominance of white-endosperm maize, which contains low levels of provitamin A and essential amino acids, has prompted breeding efforts to develop yellow and orange kernel varieties that contribute to improved human nutrition [3].

Maize production in SSA is constrained by multiple biotic and abiotic stresses, including Striga hermonthica parasitism, drought, heat, low soil nitrogen, fall armyworm, and foliar diseases. These stresses frequently occur in combination on farmers’ fields and significantly reduce yields [4]. In response, the International Institute of Tropical Agriculture (IITA) Maize Improvement Program (MIP) has prioritized development of early-maturing, multiple-stress tolerant germplasm to enhance productivity and resilience in the Guinea and Sudan Savannas [5,6]. Over the last two decades, this effort has expanded to also include the development of nutritionally enhanced yellow and orange kernel maize populations and inbred lines to support hybrid maize production in SSA [7].

Hybrid maize production relies heavily on the availability of genetically divergent parental inbred lines and a clear understanding of their heterotic patterns. Accurate knowledge of genetic diversity and population structure can assist breeders in selecting genetically divergent parental lines and may support the development of complementary breeding pools from broad-based source populations. As new early-maturing inbred lines are continually developed within the IITA-MIP, periodic genetic characterization is essential to confirm diversity levels, refine heterotic group alignments, and identify elite germplasm for future hybrid development [5,8].

Advances in molecular genotyping, particularly the use of single nucleotide polymorphism (SNP) markers, have significantly improved the resolution of genetic diversity analysis in maize. Compared to earlier marker systems such as RFLPs [9,10], AFLPs [11,12], and SSRs [13,14], SNPs offer higher genome coverage, greater reproducibility, and improved capacity to detect fine-scale population differentiation. DArTseq and DArTag SNP markers, in particular, have been successfully applied for evaluating genetic relationships and population structure in tropical maize germplasm [8,15,16].

We hypothesized that the newly developed early-maturing yellow and orange kernel maize inbred lines possess sufficient genetic diversity and well-defined population structure that can be effectively resolved using DArTag SNP markers, allowing their classification into putative molecular clusters and identification of genetically diverse lines that may be useful for future maize breeding efforts.

The objectives of this study were therefore to assess the genetic diversity of a panel of early-maturing yellow and orange kernel maize inbred lines developed within the IITA-MIP, examine their population structure, classify the inbred lines into putative molecular heterotic groups, identify a core set of genetically diverse lines with potential utility for future hybrid breeding and long-term genetic improvement.

## Materials and methods

### Development of genetic materials

The genetic materials used for this study comprised 376 multiple stress-tolerant yellow and orange endosperm elite early maturity inbred lines obtained from the Maize Improvement Program of the International Institute of Tropical Agriculture, Ibadan. The pedigree of these elite inbred lines showed that they were derived from four source populations (Table S1). These populations included two broad-based (TZE-Y Pop DT C_5_ STR C_5_ and DTE STR-Y Syn Pop C_4_) and two bi-parental populations (TZEIOR 9 x TZEIOR 56 and TZEIOR 9 x TZEIOR 108). The broad-based population TZE-Y Pop DT C_5_ STR C_5_ was developed from TZE-Y Pop DT STR. The TZE-Y Pop DT STR had been improved for *Striga* resistance and drought tolerance during the period 1996–2013 using gene introgression and the S_1_ family recurrent selection methods for five consecutive cycles as detailed by Badu-Apraku and Fakorede [7]. The DTE STR-Y Syn Pop C_4_ is an improved version of DTE STR-Y Syn Pop C_0_ developed in 2008. In a similar manner to the population TZE-Y Pop DT C_5_ STR C_5_, the population DTE STR-Y Syn Pop C_0_ was taken through four cycles of S_1_ family recurrent selection to obtain the broad-based population DTE STR-Y Syn Pop C_4_ as detailed by Badu-Apraku and Fakorede [7]. The parental inbreds of the biparental populations (TZEIOR 9 x TZEIOR 56 and TZEIOR 9 x TZEIOR 108) were developed from the broad-based population 2009 TZE-OR1 DT STR. The population 2009 TZE-OR1 DT STR was developed for high levels of provitamin A content. In the creation of the population 2009 TZE-OR1 DT STR through backcross breeding, the population 2004 TZE-Y Pop DT C_4_ STR C_4_ was crossed to a high provitamin A source [Syn –Y-STR-34-1-1-1-1-2-1-B-B-B-B-B/NC354/SYN-Y-STR-34-1-1-1 (OR1)] in the year 2007. The resulting progenies were backcrossed to the recurrent parent (2004 TZE-Y Pop DT C_4_ STR C_4_) to produce the BC_1_F_1._ Selection for endosperm with deep orange color was initiated at this stage. Selected F_1_ were advanced to the F_2_ and F_3_ stages through inbreeding. At this stage (F_3_), the lines with deep orange color were recombined to form 2009 TZE-OR1 DT STR in the year 2011. In each of the four source populations, inbred lines extraction began with the generation of the S_1_ lines through selfing. These lines were advanced to the S_2_, S_3_, and S_4_ stages of inbreeding through selfing. After each cycle of inbreeding, the lines were evaluated under artificial *Striga* infestation and induced moisture stress in the major season at Mokwa and Abuja (*Striga* infestation) and the dry season in Ikenne (drought), Nigeria. At the S_4_ stage of inbreeding, 250–300 selected lines were crossed to broad-based testers to estimate the General Combining Ability (GCA) of the lines. Based on the performance of the testcrosses, the selected S_4_ lines were advanced through selfing and selection to the S_7_ stage. Of the S_7_ inbred lines, we selected a panel of 376 yellow and orange kernel inbreds used for this study. The summary of the inbred lines, source population, kernel color, and geographical origin was presented in Table 1.

**Table 1:** Summary of the 376 early inbred lines, source population, and kernel color genotyped with 3,305 DArTag SNP markers.

Source Population	Number of inbred	Kernel Color
DTE STR-Y Syn Pop C4	70	Yellow
	56	Orange
TZEIOR 9 x TZEIOR 108	0	Yellow
	47	Orange
TZEIOR 9 x TZEIOR 56	0	Yellow
	67	Orange
TZE-Y Pop DT C5 STR C5	59	Yellow
	77	Orange

### Experimental location

Seeds of the S_7_ inbred lines were planted in the research field at International Institute of Tropical Agriculture (IITA), Ibadan. The institute’s coordinates are longitude 7^o^ 30’8” N and latitude 3^o^ 54’37” E and characterized by an altitude of 243 m above sea level. It has an annual rainfall of 1,300–2,000 mm and a bimodal rainfall distribution pattern. The annual mean temperature is 27.2°C and 25.6°C during the dry and rainy seasons, respectively.

### Leaf sample collection and genotyping procedure

Leaf samples were collected from each inbred line at three weeks after planting. The samples were stored at − 80°C for 72 h and subsequently lyophilized using a Labconco FreeZone 2.5 L System lyophilizer (Marshall Scientific, USA). Leaf discs from four lyophilized samples per inbred were punched into labeled polypropylene tubes with strip caps and transferred into 96-well deep plates for genotyping. The plates containing the leaf samples were shipped to Intertek Laboratory, Australia, for mid-density DArTag SNP genotyping. DArTag (Diversity Arrays Technology targeted genotyping) is a next-generation sequencing (NGS)-based targeted SNP genotyping platform derived from DArT technology. Unlike conventional high-density DArTseq genotyping, which generates tens of thousands of genome-wide SNP markers, DArTag uses a predefined panel of informative SNP markers (3,305) for targeted genotyping at moderate marker density. Genomic DNA was extracted, purified, and digested using a combination of restriction enzymes to reduce genome complexity. Adaptors were ligated to the resulting fragments, followed by selective PCR amplification of target genomic representations enriched for informative SNP loci. The amplified libraries were sequenced using the DArTag SNP platform, and SNP calling was conducted using the proprietary DArT analytical pipeline following the procedure described by Kilian et al. [17]. Sequence reads were aligned to the maize reference genome (B73 RefGen_v5), and the service provider performed genotype calling together with initial quality filtering prior to downstream analyses.

### Marker quality control

Marker quality control was performed in R to ensure reliable downstream population genomic analyses. The initial DArTag SNP markers dataset consisted of 3,305 SNP markers. SNP filtering was conducted based on commonly applied quality control criteria for maize SNP datasets generated using DArTag SNP markers and related genotyping platforms. A minor allele frequency (MAF) threshold of 0.05 was applied to reduce the influence of rare variants and improve the robustness of population structure analyses [15,18]. Markers with more than 20% missing data were excluded to retain informative loci with high genotype reliability while minimizing excessive missingness in downstream analyses [15]. In addition, only SNPs with read depth > 5 and genotype quality (GQ) ≥ 20 were retained to ensure high-confidence genotype calls. Only bi-allelic SNP markers were retained. This is because downstream analyses such as ADMIXTURE analysis [19], principal component analysis (PCA) [20,21], and discriminant analysis of principal components (DAPC) [22], and analysis of molecular variance (AMOVA) [22] assume bi-allelic loci for accurate estimation of allele frequencies and genetic relationships. After filtering, 1,954 high-quality SNP markers were retained for all subsequent analyses. Summary statistics including minor allele frequency (MAF), polymorphic information content (PIC), and observed and expected heterozygosity were computed using PLINK version 2.0 [23].

### Genetic diversity, linkage disequilibrium decay, and population structure analysis

The final hapmap with high-quality SNP data was converted to variant call format (VCF) and numeric genotype matrix coded as 0, 1, and 2 using the GAPIT package [24] implemented in R [25]. The identical-by-state matrix between the inbreds, a measure of the genetic relatedness or distance, was generated using PLINK 2.0 [26]. A genetic relationship matrix (additive matrix) was extracted from the numeric SNP genotype matrix for downstream analysis.

Linkage disequilibrium (LD) among SNP markers was quantified to evaluate the extent of non-random associations between alleles across the genome and to estimate the rate of LD decay within the maize inbred panel. Pairwise LD was calculated using the squared correlation coefficient (r^2^) between all marker pairs in TASSEL version 5 [27]. Physical distances between markers were obtained from SNP positional information based on the maize B73 RefGen_v5 reference genome. Pairwise LD estimates were plotted against physical distance using ggplot2 [28]. A non-linear LOESS regression curve was fitted to pairwise r^2^ values plotted against physical distance to visualise LD decay patterns. LD decay was characterised by the half-decay distance (the point at which the LOESS curve declined to half its maximum r^2^ value) and the distance at which r^2^ declined to 0.1, following commonly applied practices in maize LD studies [29,30]. Background LD was estimated from unlinked marker pairs to confirm the threshold was above the population noise floor.

To reveal the genetic stratification of the 376 elite inbred population, two methods were employed, namely; model-based population structure analysis and the discriminant analysis of principal component (DAPC) [31]. The former was performed using the sparse non-negative matrix factorization (sNMF) algorithm implemented in the LEA package [19] in R. The genotype data were converted to the LEA.geno format, with genotypes numerically coded for analysis. The number of ancestral populations (K) was evaluated from K = 1–10, with five independent repetitions performed for each K. The analyses were conducted using a fixed random seed (123) to ensure reproducibility, and the regularization parameter was set to α = 0.5. Cross-entropy values were calculated for each run and used to evaluate the fit of the alternative population-structure models and identify the most appropriate K value. For the selected K value, the ancestry coefficient (Q) matrix was extracted from the corresponding sNMF run. Individual ancestry proportions were subsequently visualized using a stacked bar plot, in which each individual was represented by a vertical bar and the relative contributions of the inferred ancestral populations were displayed as proportional segments. Individuals were ordered according to the genotype dataset, and ancestry coefficients ranged from 0 to 1, with higher values indicating a greater estimated contribution from the corresponding ancestral population. A membership probability level of ≥ 80 was used to assign the inbreds to corresponding sub-populations while inbreds with < 80 were designated as admixed. Finally, Inbred lines in the STRUCTURE bar plot were ordered according to membership coefficients within inferred subpopulations.

For DAPC using adegenet package [22], transformation into a new coordinate system was first done using PCA, thus reducing the dimensionality in the data while retaining most of the variation. Finally, discriminant analysis was applied to the principal components to maximize the separation between inferred genetic clusters. Using the IBS matrix, a dissimilarity matrix was estimated as 1-IBS similarity matrix for the 376 inbreds. This dissimilarity matrix was then used to perform phylogenetic analysis using the Analysis of Phylogenetic and Evolution (APE) package [32] implemented in R. The final phylogeny results visualized as dendrogram was used to identify major molecular clusters and patterns of genetic relatedness among the inbred lines. This was done in the ggtree package [33] in R. Analysis of molecular variance (AMOVA) which partitioned the total genetic variability into among- and within-group variances was done using GenAlEx software version 6.5 [34].

### Identification of core set for practical utilization in IITA-MIP

To establish a practically useful core subset of the 376 IITA maize inbred lines for routine breeding applications, core set selection was performed using two complementary approaches: a simulated annealing-based optimization algorithm implemented in CoreHunter 3 [35] and a greedy stepwise heuristic algorithm, the latter retained primarily for methodological comparison. All analyses were conducted on the imputed SNP genotype matrix, with alleles numerically coded as 0 (homozygous reference), 1 (homozygous alternate), and 2 (heterozygous). The target core size was set at 20% of the total collection (n = 76 lines), consistent with established recommendations for breeding-oriented core collections in crops with moderate to narrow genetic diversity [36–39].

Prior to core selection, principal component analysis (PCA) was performed on the full genotype matrix using the prcomp function in R [38] to characterize the population diversity structure. Visual inspection of the PCA biplot revealed two genetically distinct clusters along PC1, suggesting the presence of divergent subgroups within the panel. To account for this structure, k-means clustering (k = 2, nstart = 25) was applied to the first two principal components to formally assign accessions to subgroups. Core set selection was then performed using two CoreHunter configurations: an unconstrained approach applied to the full panel (CoreHunter Original), and a stratified approach in which CoreHunter was run independently within each k-means-defined cluster, with accessions allocated to the core proportionally to cluster size (CoreHunter Stratified). The stratified approach was implemented to ensure representation of genetically distinct subgroups that might otherwise be underrepresented under unconstrained distance-based optimization. CoreHunter employed the Modified Rogers Distance (MRD) as the optimization criterion, maximizing the average pairwise genetic distance among selected accessions. A fixed random seed was applied prior to all CoreHunter runs to ensure full reproducibility of results. The greedy algorithm iteratively selected the accession contributing the greatest marginal gain in allelic representation at each step, using a vectorised gain function applied across all remaining unselected accessions. Selection continued until the predefined core size was reached.

All three core sets were evaluated against the full collection using five complementary diversity metrics namely allelic richness (total number of unique alleles across all loci), expected heterozygosity (He), observed heterozygosity (Ho), the Shannon diversity index, and mean pairwise genetic distance. The PCA-based diversity space coverage was assessed by projecting all core sets onto the PCA space derived from the full collection. Minor allele frequency (MAF) distributions were compared across all sets to evaluate how well each core mirrored the allele frequency spectrum of the complete panel. All plots were constructed using the ggplot2 package [28] implemented in R.

## Results

### Summary statistics of diversity indices

**Fig 1:**
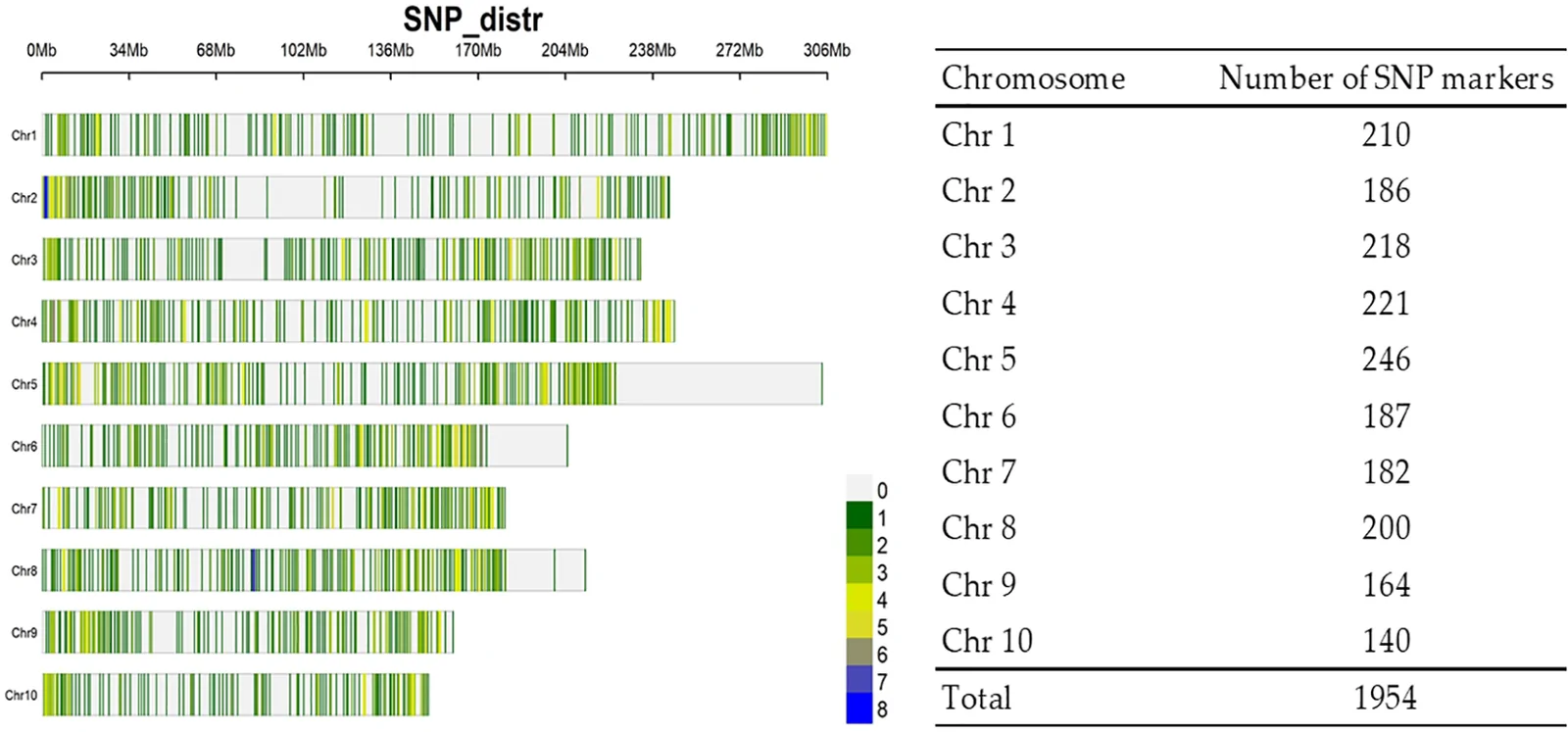
Distribution of the 1954 SNP marker across the 10 chromosomes of the maize genome. The number in the key indicated the number of SNP marker corresponding to the colour pattern depicted in the plot. Grey; no markers, dark to light green; 1 - 3 SNPs, light to dark yellow; 4 - 6 SNPs, light and deep blue; 7 and 8 SNPs, respectively.

A total of 1,954 high-quality SNP markers were retained after QC, providing adequate genome-wide coverage for diversity assessment. Across all markers, PIC averaged 0.38, and 62% of markers exceeded this mean, indicating that the dataset captured substantial polymorphism suitable for discriminating among closely related early-maturing lines. Expected heterozygosity (He) averaged 0.36, reflecting a moderate level of genetic variability typical of elite inbred panels subjected to recurrent selection. Observed heterozygosity (Ho) was extremely low (mean 0.03), consistent with the high homozygosity expected in advanced inbreds (Table 2). The highest Ho values occurred on chromosome 10, and the lowest on chromosomes 5 and 7, although differences were small. Minor allele frequency (MAF) ranged from 0.05 to 0.50 (mean 0.29), indicating that most markers captured segregating polymorphisms rather than rare variants. Chromosome 5 contained the highest number of SNPs (246), providing strong representation of this region of the genome (Fig 1 and Table 2).

**Table 2:** Diversity indices of the 376 maize inbred lines based on 1954 SNP markers.

Chromosome	H_0_	H_e_	MAF	PIC
CHR1	0.02	0.36	0.29	0.378
CHR2	0.03	0.36	0.27	0.378
CHR3	0.03	0.37	0.29	0.384
CHR4	0.03	0.37	0.28	0.380
CHR5	0.02	0.35	0.26	0.380
CHR6	0.03	0.37	0.29	0.380
CHR7	0.03	0.35	0.26	0.381
CHR8	0.02	0.37	0.29	0.382
CHR9	0.03	0.37	0.28	0.382
CHR10	0.03	0.38	0.29	0.375
Min	0.00	0.08	0.05	0.00
Average	0.03	0.36	0.28	0.38
Max	0.99	0.50	0.50	0.50

MAF = Minor allele frequency, He = Expected Heterozygosity, Ho = Observed Heterozygosity, PIC = polymorphic information content.

### Linkage disequilibrium decay

**Fig 2:**
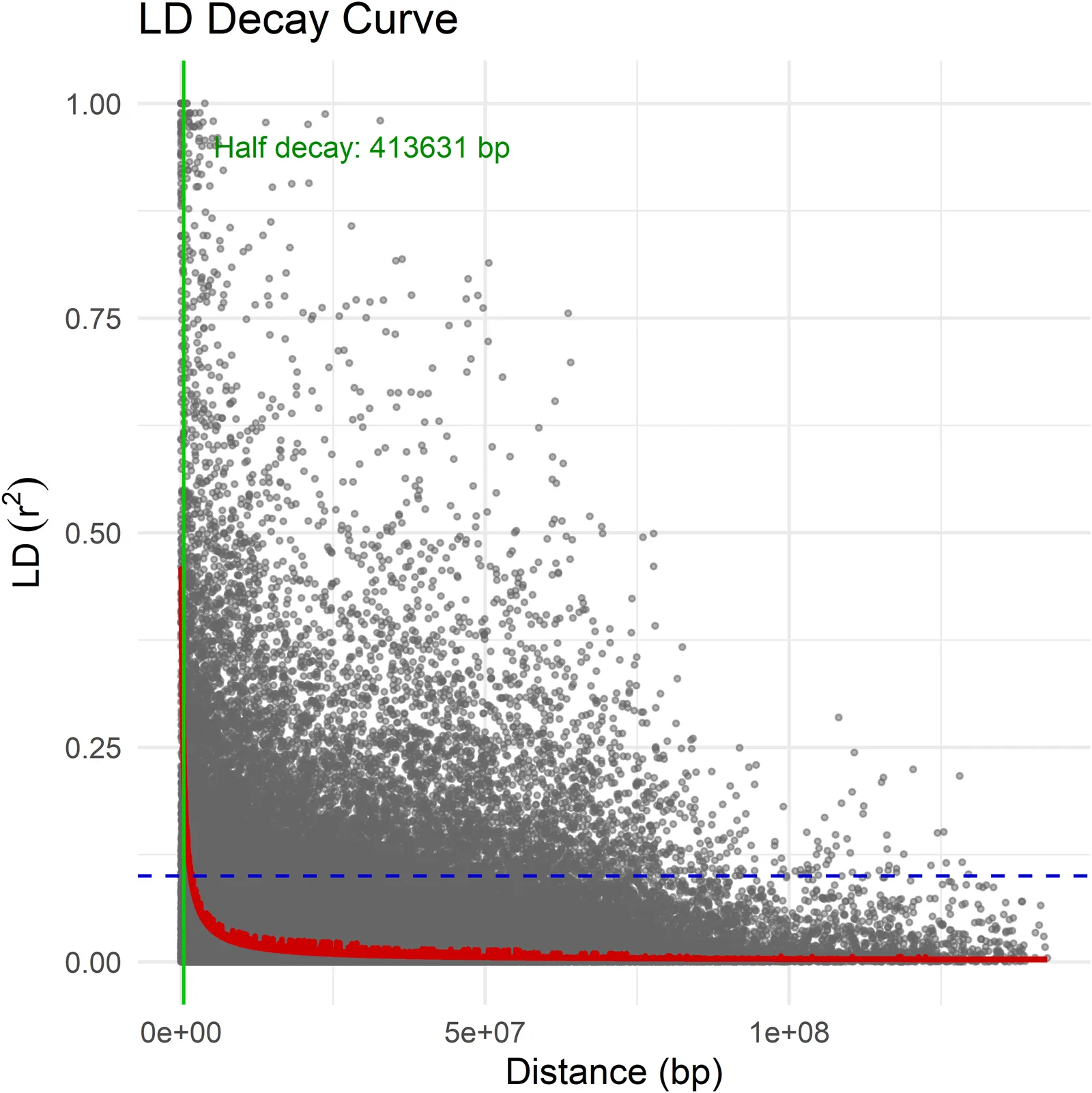
Decay of Linkage Disequilibrium (r^2^) with increasing physical distance (bp) in the early-maturing yellow and orange maize inbred lines. Red curve indicates decay curve, blue horizontal line is the applied threshold of r^2^ = 0.1, and the green vertical line is the point at which half of the initial LD dissipated.

The linkage disequilibrium (LD) analysis based on pairwise r^2^ estimates revealed a wide distribution of LD values across the genome, ranging from 0 to 1, with an overall mean r^2^ of 0.046. As shown in the LD decay plot in Fig 2, a dense cluster of high-LD points (r^2^ > 0.8) occurred at very short physical distances (< 50 kb), after which LD declined rapidly with increasing marker separation. The fitted non-linear regression curve (red line) showed a steep decay pattern, approaching background LD levels at larger distances. Using the commonly applied threshold of r^2^ = 0.1 (blue horizontal line), LD decayed to this level at approximately 413,631 bp (green vertical line). This distance represents the point at which half of the initial LD was dissipated. Beyond approximately 40 Mb, LD values stabilized close to zero, indicating minimal long-range LD. Overall, the rapid decline in LD suggests substantial historical recombination within the early-maturing yellow and orange maize panel (Fig 2).

### Population structure of the 376 elite early yellow and orange inbred lines

**Fig 3:**
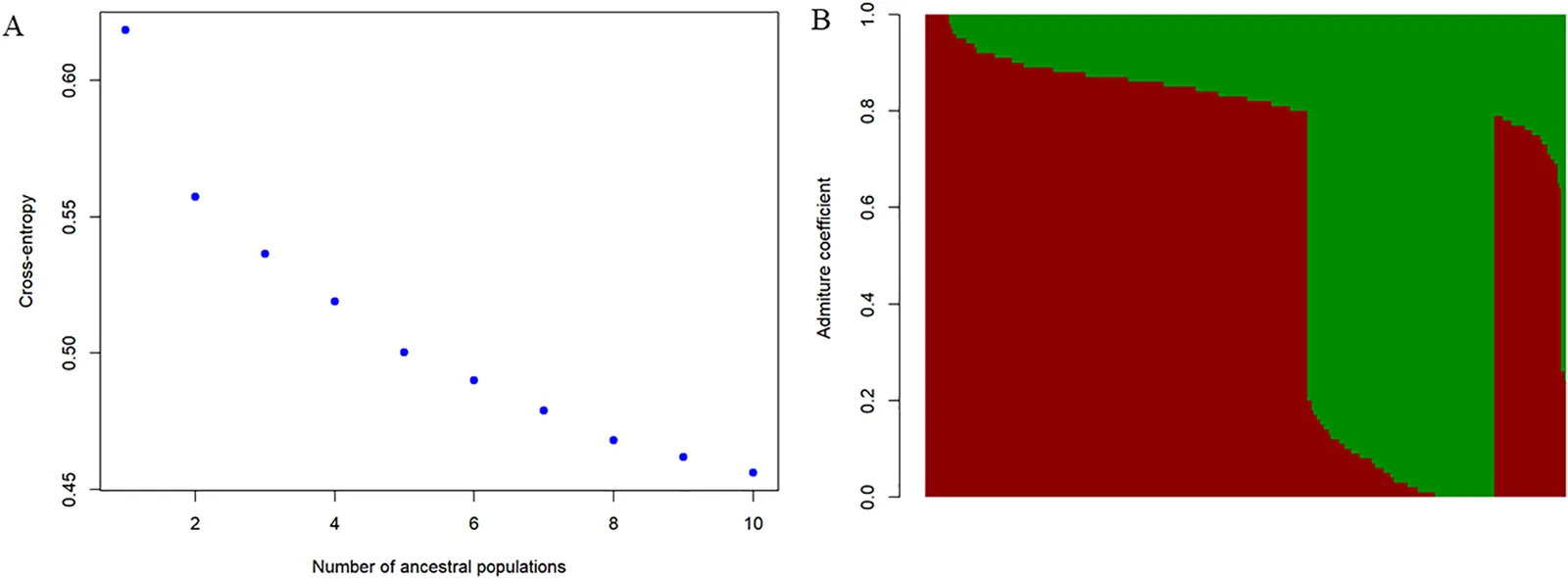
Population structure of 376 elite yellow and orange maize inbreds using 1954 DArTtag-derived SNP markers. (A) Graph of cross-entropy versus number of ancestral population showing very large drop up to K = 2, (B) Plot view of admixture coefficient based on membership probability of 80% showing sub-population one (red) and two (green).

**Fig 4:**
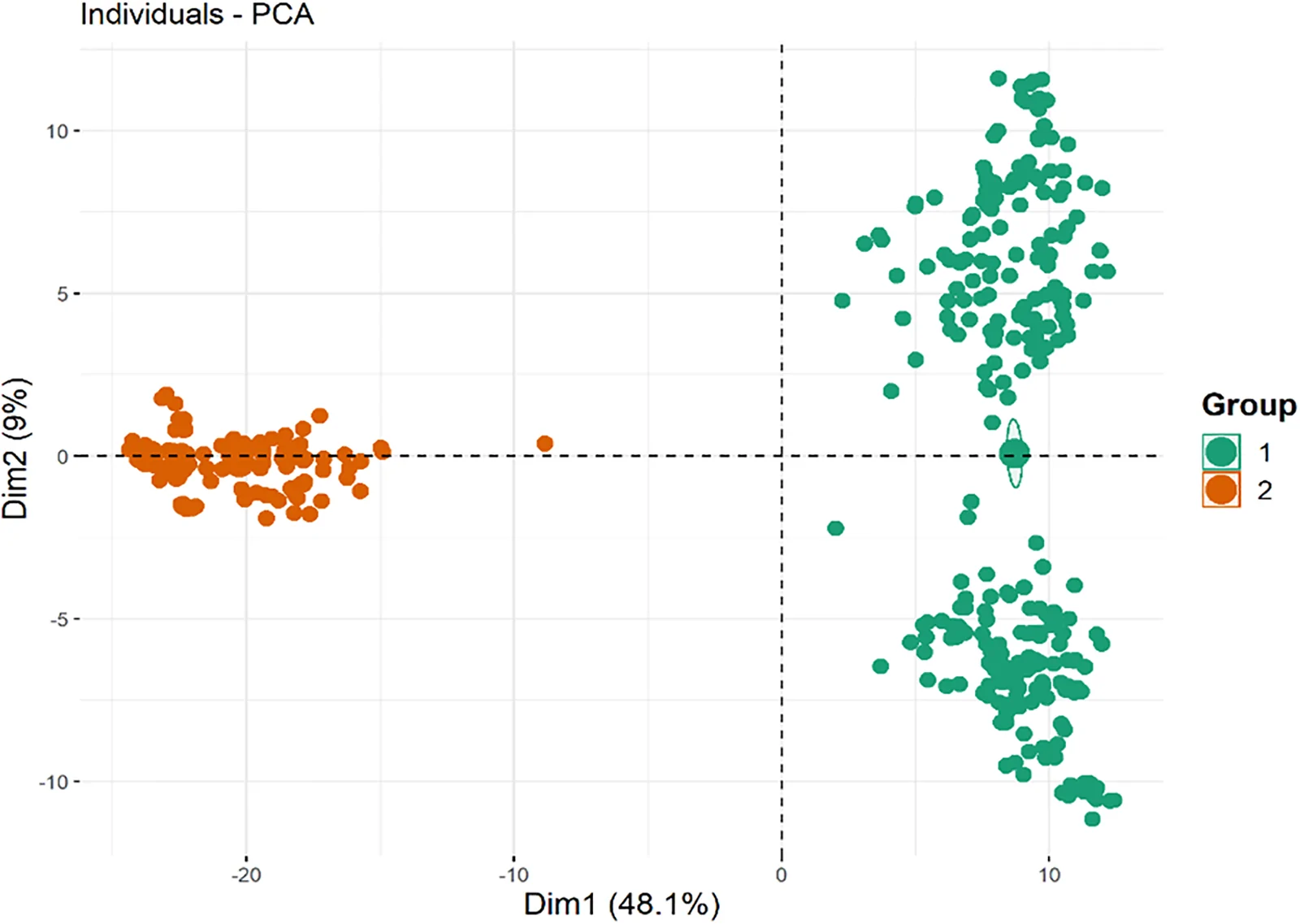
Principal component analysis showing the pattern of population stratification that characterized the 376 early yellow and orange inbred lines assessed with 1954 SNP markers. Group one in green color with two subgroups and group two in red color.

Admixture analysis revealed optimum population stratification at K = 2, indicating that the panel of 376 early yellow/orange inbred lines resolved into two major genetic clusters (Fig 3A). Using an ancestry threshold of 0.80, 357 lines (95%) were assigned with high confidence, while 39 lines (10%) exhibited admixture. Subpopulation 1 contained 225 lines, and Subpopulation 2 contained 112 lines (Fig 3B and Table S2). The discriminant analysis of principal component (DAPC) confirmed the presence of two main clusters, with ~95% correspondence to STRUCTURE assignments (Table S3). Most admixed individuals from STRUCTURE grouped into cluster 1 under DAPC, suggesting that this cluster contains more within-group diversity or more recent gene flow. The Principal Component Analysis (PCA) explained 57% of total variation in the first two components (Fig 4). While the two main clusters were distinct, Subpopulation 1 showed an internal split, suggesting additional sub-structuring that might reflect the breeding history of the underlying source populations. Biologically, this pattern suggests that the germplasm has undergone extensive historical recombination during its breeding history. The relatively short LD decay distance is consistent with observations from diverse tropical maize populations and indicates substantial genetic reshuffling across generations. Such patterns may provide useful opportunities for future genetic studies aimed at understanding the genomic architecture of important agronomic traits. This correspondence between molecular clustering and pedigree background suggests that the SNP data captured population stratification associated with breeding history and recurrent selection.

### Genetic distance and inferred molecular heterotic groups of 376 early yellow and orange inbreds using 1954 SNP markers

**Fig 5:**
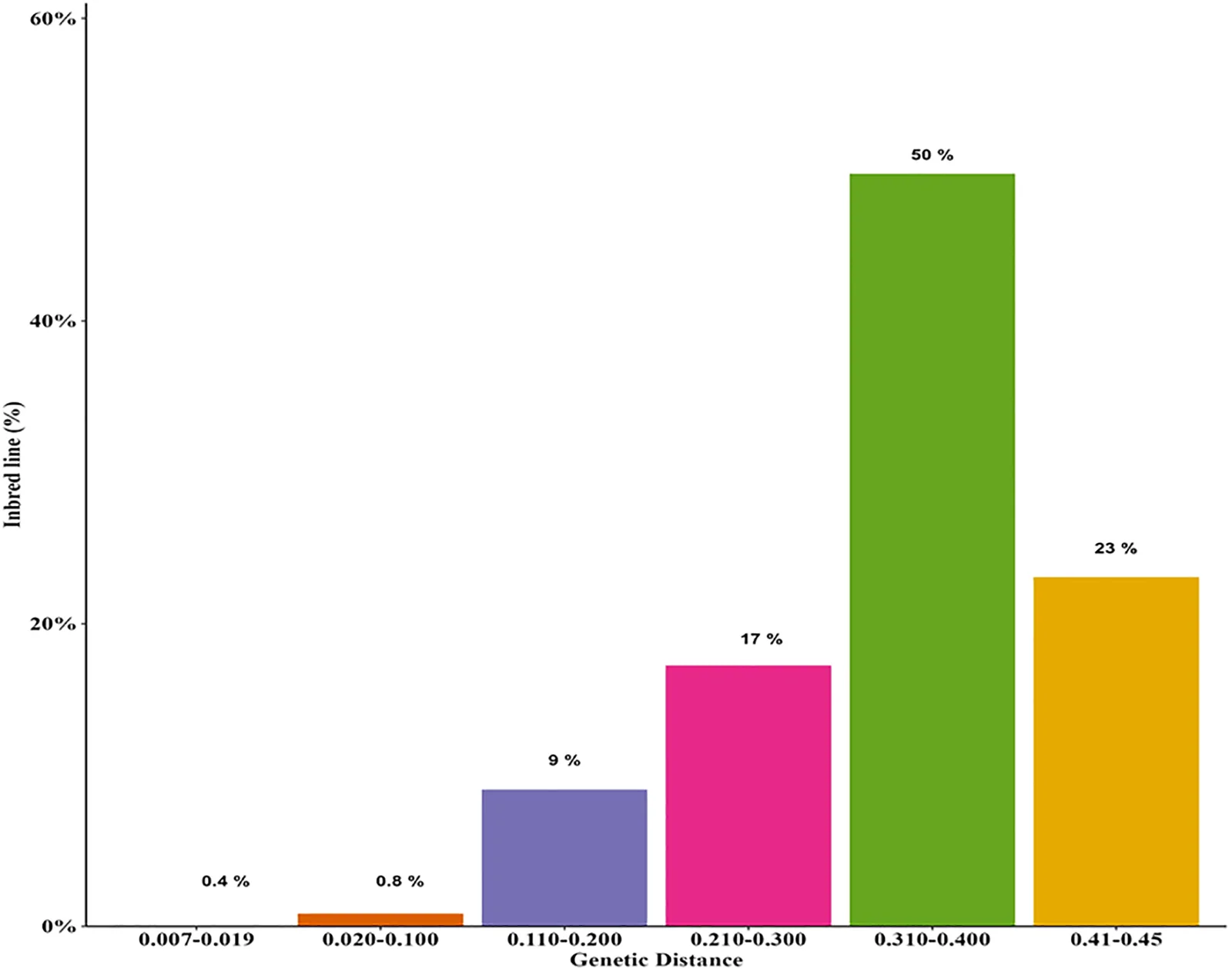
Frequency distribution of the dissimilarity matrix of 376 early yellow and orange elite inbred lines genotyped using 1954 SNP markers.

**Fig 6:**
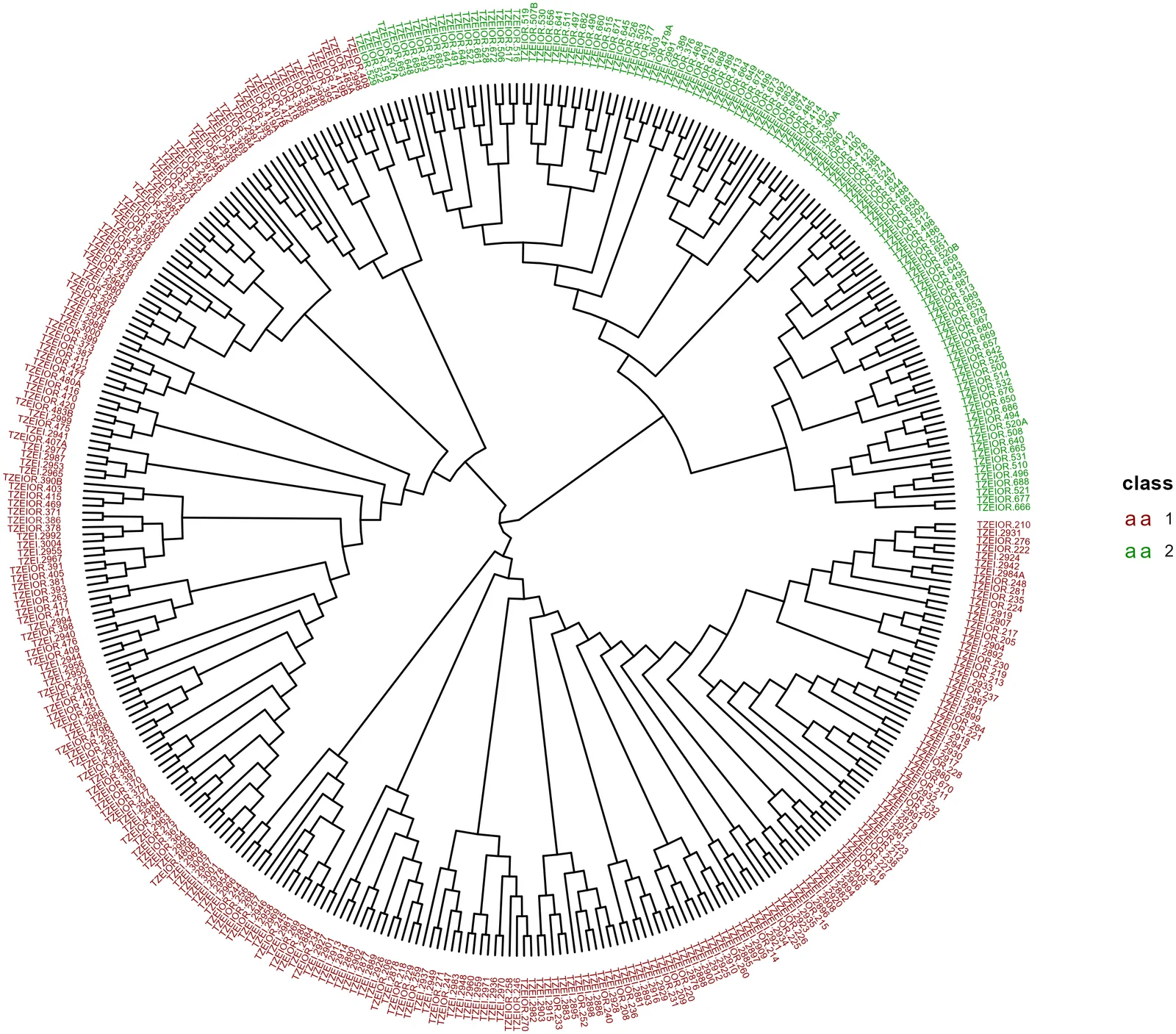
Phylogenetic analysis showing the molecular heterotic groups of the 376 elite yellow and orange inbred lines. Group one in red color with two subgroups and group two in green color.

The pairwise genetic distances (GD) among the 376 early-maturing yellow and orange inbred lines ranged from 0.006 to 0.452, with a mean of 0.361, and more than 70% of the comparisons exceeding this average. This indicates broad allelic divergence within the panel (Fig 5). Cluster analysis based on these GD values partitioned the lines into two major molecular heterotic groups (HGs). The first group (HG1) comprised 282 inbreds (approximately 75% of the collection), representing a mixture of yellow- and orange-endosperm types. HG1 showed a relatively high mean GD (0.362), reflecting considerable within-group diversity, and included lines predominantly derived from TZE-Y Pop DT STR C5 and DTE STR-Y Syn Pop C4. The second group (HG2), consisting of 94 inbreds (~25%), was dominated by orange-endosperm lines originating mainly from the TZEIOR 9 × TZEIOR 56 and TZEIOR 9 × TZEIOR 108 families. HG2 exhibited a lower mean GD (0.227), suggesting that it represents a genetically narrower but distinct subgroup (Fig 6 and Table 3). Biologically, the observed clustering corresponds closely to known IITA heterotic pools. The members of inferred HG1 align with the drought-tolerant and Striga-resistant early populations broadly used as maternal testers, while HG2 captures the orange-derived pool that typically forms the contrasting tester group. This congruence between molecular clustering and known breeding history suggests the potential utility of these molecular groupings for future hybrid evaluation and tester development studies.

**Table 3:** Genetic distance characterizing the identified molecular heterotic groups.

	HG_1	HG_2
Minimum	0.006 (TZEI 2772 and TZEI 2761)	0.008 (TZEI 2477 and TZEI 2490)
Maximum	0.442 (TZEI 2832 and TZEI 2273)	0.350 (TZEI 2273 and TZEI 2748)
Average	0.362	0.227

HG_1, putative heterotic group one; HG_2, putative heterotic group two.

### Relationship between source populations and putative heterotic groups

**Fig 7:**
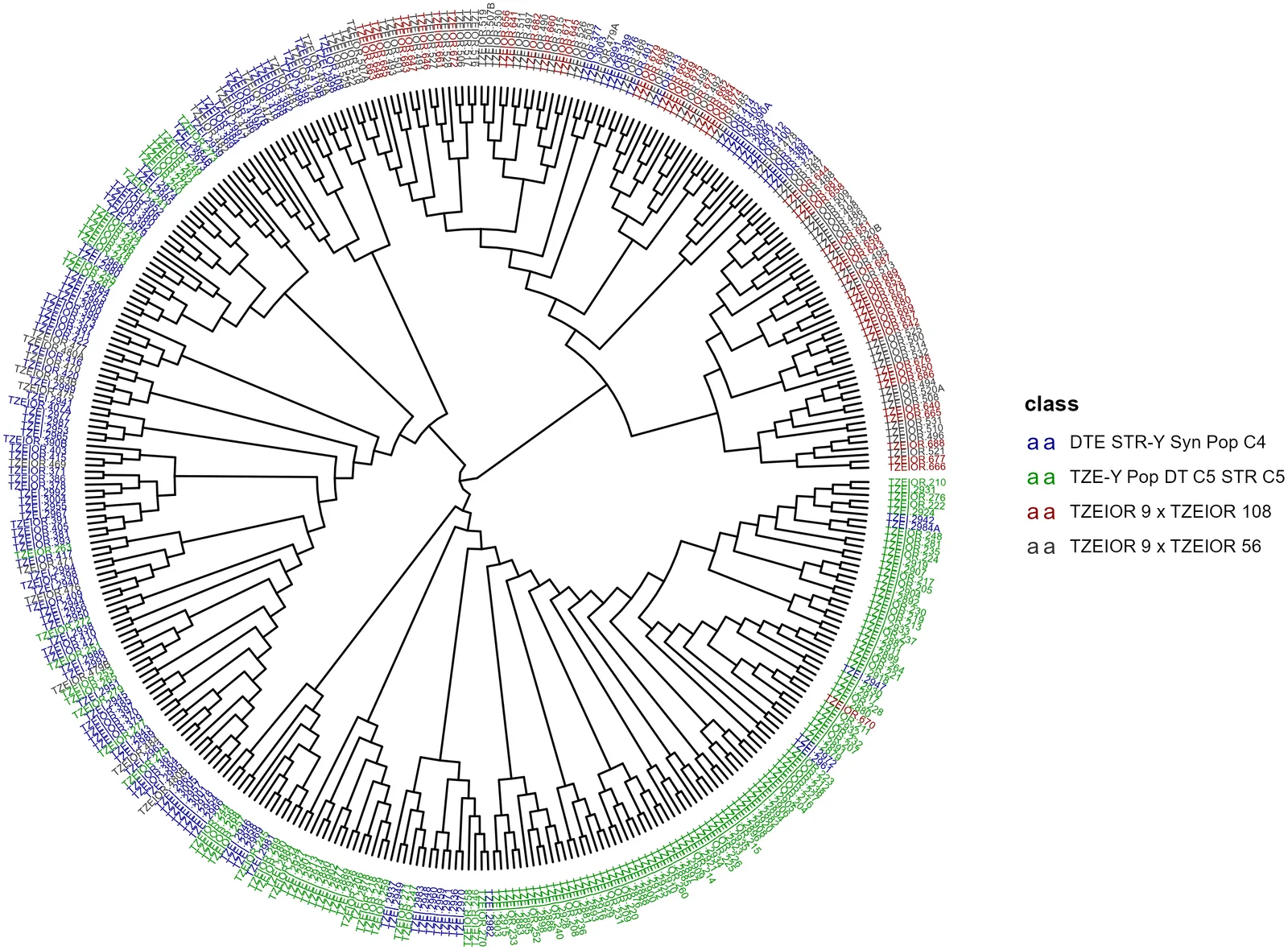
Phylogenetic relatedness among 376 early-maturing yellow and orange maize inbreds based on the source populations from which the inbreds were developed. HG_1 means putative heterotic group one and HG_2 means putative heterotic group two.

Principal component analysis incorporating pedigree information confirmed that the clustering of inbred lines reflected both their genetic structure and their breeding origins. Lines belonging to HG1 were primarily derived from TZE-Y Pop DT STR C5 (52%) and DTE STR-Y Syn Pop C4 (41%), demonstrating that these two sources share substantial allelic overlap and have contributed heavily to the formation of this putative heterotic pool. Conversely, HG2 contained a larger proportion of inbreds from the TZEIOR 9 × TZEIOR 56 (45%) and TZEIOR 9 × TZEIOR 108 (41%) families, indicating that these populations form a genetically distinct lineage that aligns with the second putative heterotic group (Fig 7). The strong association between pedigree background and molecular grouping highlights the extent to which historical selection, recurrent breeding cycles, and tester usage have structured the gene pool into two complementary putative heterotic patterns. This alignment supports the consistency between pedigree background and molecular clustering patterns.

### Analysis of molecular variance and genetic differentiation

Analysis of molecular variance (AMOVA) revealed significant but moderate population stratification. When the inbred lines were grouped according to the two putative heterotic clusters, 14% of the total genetic variance was attributable to differences between groups, while 86% of the variance was observed within groups. The corresponding FST value (0.139, P < 0.001) indicates moderate genetic differentiation, consistent with levels commonly reported among maize heterotic pools (Table 4). When AMOVA was performed using the four major pedigree sources, the variance explained among populations was 10% (FST = 0.099, P < 0.001), again reflecting moderate but meaningful differentiation (Table 4). The pairwise FST estimates further clarified the relationships among the source populations. Very low differentiation (FST = 0.026–0.028) among the closely related populations suggests recent shared ancestry or ongoing introgression. In contrast, higher divergence between TZE-Y Pop DT STR C5 and the TZEIOR-derived populations (FST ≈ 0.145) supports the presence of two genetically differentiated gene pool that warrant further evaluation in heterotic hybrid development (Table 5). Collectively, these results demonstrate that the structure of the germplasm is suitable for heterotic group formation and may support future tester selection and hybrid breeding efforts.

**Table 4:** Analysis of molecular variance among and within maize inbred subpopulations.

	Source	DF	SS	MS	Est. Var.	% Var	Fst	P
	Among HG	1	9308.74	9308.74	56.67	14%	0.139	0.001
K = 2	Within HG	374	131179.3	350.75	350.75	86%		
	Total	375	140488.00		407.41	100%		
	**Source**	**DF**	**SS**	**MS**	**Est. Var.**	**% Var**	**Fst**	**P**
	Among Ped.	3	11240.90	3746.97	38.23	10%	0.099	0.001
Pedigree	Within Ped.	372	129247.10	347.44	347.44	90%		
	Total	375	140488.00		385.67	100%		

Among HG: among-putative heterotic groups; Within HG: within-putative heterotic groups; Among Ped.: among-pedigrees; Within Ped.: within-pedigrees; DF: degree of freedom; SS: sum of squares; MS: mean squares; Est.Var: estimated variance; %: percentage variance; Fst: Pairwise differentiation; P: significance level

**Table 5:** Pairwise genetic differentiation and gene flow based on pedigree information of the 376 elite yellow and orange inbreds.

Source	DTE STR-Y Syn Pop C4	TZEIOR 9 x TZEIOR 108	TZEIOR 9 x TZEIOR 56	TZE-Y Pop DT C5 STR C5
DTE STR-Y Syn Pop C4		0.001	0.001	0.001
TZEIOR 9 x TZEIOR 108	0.026		0.001	0.001
TZEIOR 9 x TZEIOR 56	0.056	0.028		0.001
TZE-Y Pop DT C5 STR C5	0.149	0.145	0.083	
	**DTE STR-Y Syn Pop C4**	**TZEIOR 9 x TZEIOR 108**	**TZEIOR 9 x TZEIOR 56**	**TZE-Y Pop DT C5 STR C5**
DTE STR-Y Syn Pop C4	0.000			
TZEIOR 9 x TZEIOR 108	18.535	0.000		
TZEIOR 9 x TZEIOR 56	8.448	17.164	0.000	
TZE-Y Pop DT C5 STR C5	2.866	2.956	5.506	0.000

### Core subset selection and diversity characterization

A core subset of 76 inbred lines (20% of the full panel of 376 accessions) was identified from the IITA maize inbred collection using the three complementary selection approaches (CoreHunter Original, stratified CoreHunter; and Greedy stepwise algorithm). The diversity metrics for all three core sets relative to the full collection are summarized in Table 6. The PCA of the full genotype matrix revealed two genetically distinct clusters along PC1, which explained 12.3% of the total genetic variance (PC2 = 4.9%), indicating the presence of at least two divergent subgroups within the panel. This population structure informed the stratified CoreHunter approach, in which proportional sampling from each cluster was enforced prior to distance-based optimization.

**Table 6:** Diversity metrics showing allelic richness, expected heterozygosity (He), observed heterozygosity (Ho), Shannon diversity index, mean pairwise genetic distance, and the proportion of allelic richness retained relative to the full collection for the full collection and three core subsets of 376 IITA early-maturing maize inbred lines.

Set	Number of Accessions	Allelic Richness	Expected Heterozygosity	Observed Heterozygosity	Shannon Diversity	Mean Genetic Distance	Proportion of Allelic Retained
Full	376	5814	0.36	0.03	0.62	0.73	1.00
CoreHunter_Original	76	4984	0.37	0.02	0.59	0.74	0.86
CoreHunter_Stratified	76	4834	0.37	0.02	0.58	0.74	0.83
Greedy	76	5636	0.35	0.04	0.62	0.69	0.97

### Allelic richness and retention

The greedy core retained the highest proportion of allelic richness relative to the full collection (96.9%; 5,636 of 5,814 alleles), compared to 85.7% for CoreHunter Original (4,984 alleles) and 83.1% for CoreHunter Stratified (4,834 alleles). This outcome is expected given that the greedy algorithm directly optimizes for allelic richness at each selection step, whereas CoreHunter optimises for genetic distance — a fundamentally different criterion.

### Heterozygosity and allele frequency fidelity

**Fig 8:**
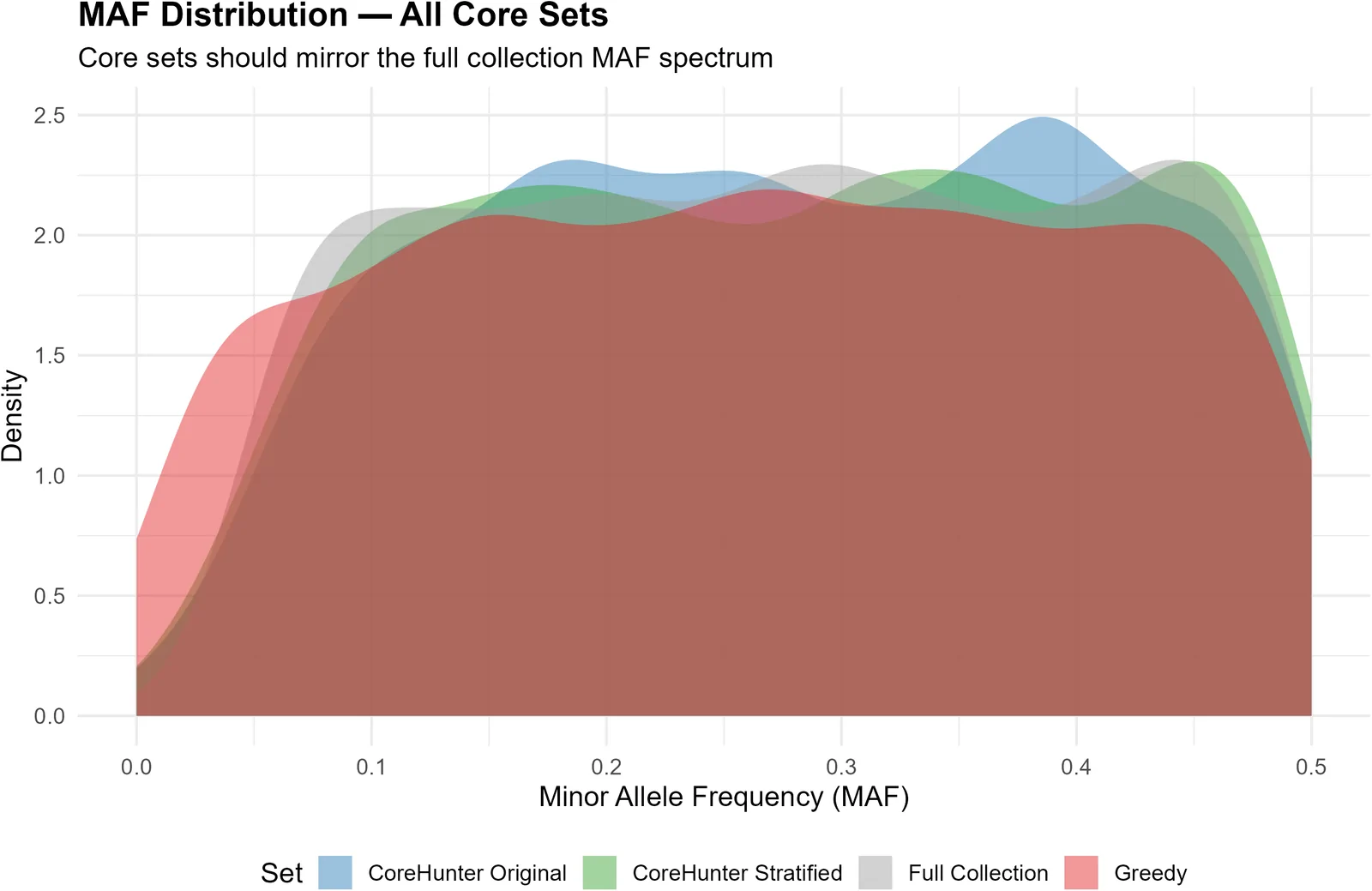
Minor allele frequency (MAF) distribution across the full collection and three core subsets of IITA early-maturing maize inbred lines. Kernel density estimates of MAF are shown for the full collection (grey, n = 376), CoreHunter Original (blue, n = 76), CoreHunter Stratified (green, n = 76), and Greedy core (red, n = 76), based on 1,954 DArTag SNP markers.

Both CoreHunter configurations more closely preserved the expected heterozygosity (He) of the full collection (He = 0.36) compared to the greedy core. CoreHunter Original achieved the closest match (He = 0.37), followed by CoreHunter Stratified (He = 0.37), while the greedy core showed a notable reduction (He = 0.34), indicating underrepresentation of balanced allele frequency loci. Observed heterozygosity (Ho) in the full collection was low (Ho = 0.03), consistent with the inbred nature of the panel. The greedy core inflated Ho to 0.04, exceeding the full collection value, suggesting preferential selection of lines with residual heterozygosity. Both CoreHunter cores maintained Ho values below the full collection (0.02 and 0.02 for Original and Stratified, respectively), reflecting more faithful representation of the inbred panel’s homozygosity structure. Minor Allele Frequency (MAF) distribution comparisons further confirmed that both CoreHunter configurations provided a closer approximation of the full collection’s allele frequency spectrum, while the greedy core showed relative underrepresentation of low-frequency alleles (MAF < 0.1) (Fig. 8).

### Genetic distance and diversity space coverage

**Fig 9:**
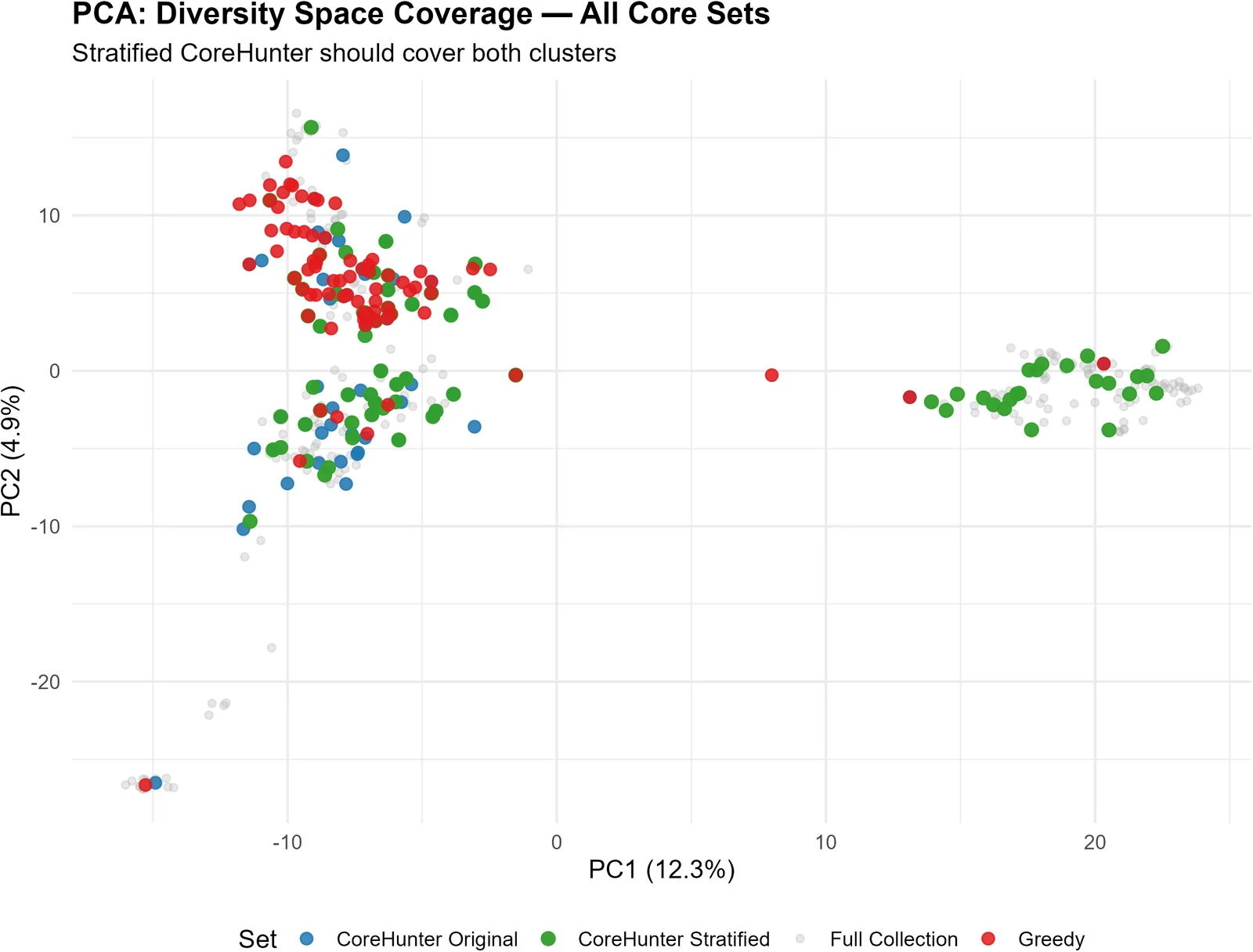
Principal component analysis (PCA) of genetic diversity space coverage for the full collection and three core subsets of 376 IITA early-maturing maize inbred lines generated from 1,954 DArTag SNP markers. Each point represents a single accession projected onto PC1 (12.3% of total variance) and PC2 (4.9% of total variance). The full collection is represented by grey points (n = 376). Accessions selected by the unconstrained CoreHunter (CoreHunter Original), population-structure-aware stratified CoreHunter (CoreHunter Stratified), and greedy stepwise algorithm are shown in blue, green, and red, respectively (each n = 76).

CoreHunter Original achieved the highest mean pairwise genetic distance among selected accessions (0.74), exceeding the full collection mean (0.73) but similar to the stratified core (0.74), demonstrating its effectiveness in maximizing genetic distinctness among core entries. The greedy core yielded the lowest mean genetic distance (0.69), indicating greater genetic similarity among its selected lines and reduced utility for maximizing crossing diversity. PCA-based diversity space coverage revealed a critical limitation of the unconstrained CoreHunter approach, despite achieving the highest mean genetic distance overall, CoreHunter Original failed to include representatives from the genetically distinct cluster at the high PC1 extreme, which likely corresponds to a divergent putative heterotic group within the panel. In contrast, both the greedy core and the stratified CoreHunter core captured representatives from this cluster, demonstrating the importance of accounting for population structure in core set selection. The stratified CoreHunter core achieved comparable mean genetic distance (0.74) to the unconstrained approach while ensuring complete coverage of both genetic clusters, representing the most balanced solution across all evaluated criteria (Fig. 9).

## Discussion

### Genetic diversity and marker informativeness

This study employed 1,954 high-quality DArTag SNP markers to characterize the genetic diversity, population structure, and molecular heterotic patterns of 376 early-maturing yellow and orange maize inbred lines. The levels of expected (He) and observed heterozygosity (Ho) were comparable to findings from similar studies in West African and tropical maize [8,15], and consistent with the reduction in heterozygosity expected in advanced inbred lines. The moderate values of PIC and the distribution of minor alleles further indicate that the SNP panel had sufficient discriminatory power to differentiate closely related lines. These patterns generally align with observations from International Maize and Wheat Center (CIMMYT) and the Water Efficient Maize for Africa (WEMA) early-maturity maize panels where moderate PIC values (0.25–0.35) and a high proportion of rare alleles have been reported [40,41]. This is typical of tropical germplasm subjected to recurrent selection and drought-stress screening

### Linkage disequilibrium and recombination history

The linkage disequilibrium (LD) decay pattern observed in this study is consistent with expectations for genetically diverse, predominantly outcrossing maize germplasm. The relatively low mean r^2^ (0.046) and the rapid decline to the r^2^ = 0.1 threshold at approximately 413 kb indicated a high historical recombination rate within these yellow/orange early-maturing lines. This decay distance falls within the range reported for tropical maize breeding materials from CIMMYT, which typically show LD decay between 100 kb and 500 kb depending on population diversity and marker density [40,42,43]. The dense cluster of high r^2^ values near zero distance reflects the presence of tightly linked SNPs within haplotype blocks; however, their rapid breakdown confirms that the population is not under genetic bottleneck. Biologically, this pattern suggests that the germplasm has undergone extensive recombination during its breeding history. The observed LD decay pattern suggests substantial historical recombination within the panel and indicates that the germplasm may provide useful resolution for future association studies in tropical maize breeding populations.

### Population structure and putative heterotic group classification

Both STRUCTURE and DAPC consistently revealed two major genetic clusters within the panel. This agrees with reports from CIMMYT, USDA-GRIN, and West African breeding programs, which frequently identify two or three predominant heterotic patterns in short to long duration maturity maize populations [44,45]. In our study, these clusters corresponded closely with the breeding origins of the lines: Cluster 1 drew largely from drought-tolerant TZE-Y and DTE STR-Y populations, while Cluster 2 comprised predominantly TZEIOR-derived families. Similar alignment between population structure and pedigree has been observed in CIMMYT early-drought panels and WEMA drought-tolerant hybrids, where genetic clustering often reflects long-term tester usage and recurrent selection pipelines.

The finding that each putative heterotic group contained lines from multiple source populations indicates substantial allele sharing within broader IITA breeding pools. This is consistent with previous studies showing that elite tropical breeding programs tend to maintain moderate differentiation while allowing gene flow among synthetics and broad-based populations to preserve adaptive diversity [46,47]. The presence of shared ancestry is biologically meaningful because it suggests that breeders have successfully combined favorable alleles for early maturity and stress adaptation across multiple selection cycles.

AMOVA results showed that most genetic variation occurred within rather than between groups, which is typical for outcrossing species like maize and aligns with global maize diversity studies [44,48]. The moderate FST values (0.099–0.139) observed here are comparable to levels reported in CIMMYT stress-tolerant germplasm and indicate moderate differentiation among the identified molecular groups. Pairwise FST patterns also reflected known breeding history, with the tight relatedness among TZEIOR populations attributable to shared founder lines. In contrast, greater divergence between the TZE-Y and TZEIOR families corresponds to the complementary gene pools historically exploited to generate high-yielding early-maturing hybrids for West Africa.

### Core subset selection and practical utility

The construction of a genetically optimized core subset from a large germplasm panel is a critical step in bridging molecular diversity characterization and practical breeding application. While the concept of core collections was first operationalized by Brown [36] the methodological debate over the most appropriate selection criterion, allelic richness maximization versus genetic distance optimization, remains active in the plant genetic resources literature [35]. The present study contributes to this discussion by empirically comparing three selection approaches under a structured tropical maize panel where the presence of distinct subgroups introduces a layer of complexity not adequately addressed by conventional single-objective optimization. The superior allelic richness retention of the greedy core (96.9%) relative to both CoreHunter configurations is expected given its explicit optimization objective, and is broadly consistent with the 70–95% retention range reported in earlier core collection studies across crops [37]. However, allelic richness alone is an incomplete criterion for evaluating core set utility in a breeding program. Maximizing the count of unique alleles captured does not necessarily ensure that those alleles are present at frequencies, or in genetic backgrounds, that render them accessible to selection. The inflated observed heterozygosity in the greedy core (Ho = 0.04 vs. Ho = 0.02 in the full collection) raises a more substantive concern. In a panel of advanced inbred lines, Ho should be uniformly low, and a core that disproportionately selects lines with residual heterozygosity may inadvertently introduce genetic instability into downstream crossing programmes. This finding echoes cautions raised by Thachuk et al. [47] and Odong et al. [48], who demonstrated that allele-counting heuristics can produce cores that are statistically rich but practically suboptimal when genetic background quality is not accounted for.

The unconstrained CoreHunter approach, by contrast, achieved the highest mean pairwise genetic distance (0.74) and maintained expected heterozygosity most faithfully relative to the full collection, reflecting its theoretical advantage in maximizing the representativeness of the selected subset as a whole. These properties are particularly relevant for genomic selection training population design, where diversity of the reference panel, rather than raw allele count, is the primary determinant of prediction accuracy across environments [49–51]. Nevertheless, the failure of unconstrained CoreHunter to capture the genetically divergent TZEIOR subgroup reveals a well-documented limitation of mean-based distance optimization: when a collection contains minority clusters, maximizing average pairwise distance across the full panel tends to oversample the dominant cluster at the expense of underrepresented subgroups [35,48]. In the context of maize hybrid breeding, where exploiting complementary heterotic pools is the primary route to yield gain, this is not a minor statistical artefact but a consequential practical failure, a core set that excludes one group cannot support the design of crosses that capture maximum heterosis.

The stratified CoreHunter approach directly addresses this limitation by imposing proportional representation of genetically defined subgroups prior to distance optimization, a strategy conceptually aligned with the stratified sampling frameworks advocated for diverse germplasm collections [52]. By running CoreHunter independently within each k-means-defined cluster, the stratified approach preserves the genetic distance optimization advantages of the algorithm while correcting for the cluster coverage bias inherent in unconstrained selection. The resulting core maintained competitive diversity metrics across all five evaluated criteria and, critically, was the only approach to ensure representation from both putative heterotic subgroups in the PCA diversity space, a finding with direct implications for hybrid programme design. The alignment between the stratified core’s cluster representation and the molecular clusters identified by STRUCTURE and DAPC supports the consistency of the inferred population structure.

The predominance of drought-tolerant population derivatives (TZE-Y Pop DT STR C5, DTE STR-Y Syn C4) among core selections further reinforces the practical relevance of the core subset for stress-adapted breeding in sub-Saharan Africa, where terminal drought and low soil nitrogen remain the primary yield-limiting constraints [53–55]. From a genomic prediction standpoint, a core subset that is genetically diverse, heterotically balanced, and derived from stress-adapted backgrounds is particularly well-positioned to serve as a training population for multi-environment genomic selection models. This is consistent with simulation and empirical studies demonstrating that training population diversity and genetic relatedness to the prediction set are the strongest determinants of cross-environment prediction accuracy [51]. Beyond genomic selection, the stratified core also represents an efficient resource for multi-parent advanced generation intercross (MAGIC) population development, tester identification, and marker-assisted introgression of stress tolerance and possibly nutritional quality alleles.

Collectively, these findings underscore that the choice of core selection algorithm is not merely a computational decision but a biologically consequential one that should be informed by the population structure of the germplasm under consideration. For breeding collections with clear heterotic group differentiation, as demonstrated here and commonly observed in CIMMYT and IITA tropical maize panels [55], unconstrained single-objective optimization methods risk producing cores that are statistically elegant but structurally incomplete. The integration of population structure information into core selection, as implemented in the stratified CoreHunter framework presented here, offers a more robust and breeding-relevant approach that we recommend for adoption in similar germplasm optimization exercises across tropical cereal improvement programmes.

### Breeding implications and future directions

Overall, the genetic structure revealed in this study has direct implications for breeding strategies. The two putative heterotic groups identified here correspond to established tester patterns used in early-maturity breeding and may provide a useful framework for future hybrid development pipelines. The considerable within-group variation provides a broad genetic base for selecting parental lines with potential combining ability, while the moderate between-group divergence supports the development of heterotic hybrids targeting drought-prone and short-season environments typical of the savannas of sub-Saharan Africa. Although nutritional traits such as provitamin-A concentration were not measured in this study, the inclusion of both yellow and orange endosperm lines ensures that the diversity characterized here is directly relevant to nutritional breeding pipelines aimed at biofortified maize. Additionally, though the present study focused on genomic characterization, the evaluated inbred lines were previously developed and selected within the IITA-MIP under recurrent screening for grain yield performance, stress tolerance, and kernel quality traits. Therefore, the molecular diversity observed here reflects germplasm with demonstrated breeding relevance for tropical maize improvement. Future studies integrating phenotypic evaluation, particularly hybrids generated from the identified putative heterotic groups, will be essential to validate combining ability patterns and translate the molecular diversity observed here into improved cultivar performance under field conditions.

## Conclusions

This study provided a comprehensive genomic characterization of 376 early-maturing yellow and orange maize inbred lines using 1,954 high-quality DArTtag SNP markers. The low observed heterozygosity confirmed the advanced inbred status of the lines, while the moderate expected heterozygosity, PIC values, and broad minor allele frequency distribution indicated substantial genetic variation within the panel. Linkage disequilibrium decayed rapidly, reaching the r^2^ = 0.1 threshold at approximately 413 kb, a pattern consistent with genetically diverse tropical maize and supportive of high-resolution mapping and effective genomic selection. Population structure analyses and genetic distance–based clustering consistently revealed two major putative heterotic groups that aligned with the breeding history of drought-tolerant TZE-Y and DTE STR-Y populations and the TZEIOR families. To enhance the practical utility of the genomic insights, a core set capturing nearly all allelic richness in only 15% of the original panel was identified. This optimized subset spans both putative heterotic groups. The results demonstrate that the germplasm possesses a rich and structured genetic base that can be employed for hybrid development, tester optimization, and the design of efficient genomic selection training populations. The integration of diversity analyses, LD patterns, and core-set optimization provides a robust foundation for accelerating the development of breakthrough (high-yielding, stress-resilient, and nutritionally enhanced) early-maturing maize hybrids for sub-Saharan Africa.

## Supporting information

S1 Table List of inbreds that constitute the study population and their pedigree. (DOCX)

S2 Table Population stratification based on the Bayesian statistics approach implemented in STRUCTURE revealed two genetic groups with 5% level of admixt. (DOCX)

S3 Table Population stratification based on the discriminant analysis of principal components approach revealed two genetic groups. (DOCX)

## References

[1] Zhang X, Zhang H, Li L, Lan H, Ren Z, Liu D, et al. Characterizing the population structure and genetic diversity of maize breeding germplasm in Southwest China using genome-wide SNP markers. BMC Genomics. 2016;17(1):697. doi: 10.1186/s12864-016-3041-3 27581193 PMC5007717

[2] Badu-Apraku B, Obisesan O, Olumide OB, Toyinbo J. Gene action, heterotic patterns, and inter-trait relationships of early maturing pro-vitamin a maize inbred lines and performance of testcrosses under contrasting environments. Agronomy. 2021;11(7):1371. doi: 10.3390/agronomy11071371

[3] Bonkoungou TO, Badu-Apraku B, Adetimirin VO, Nanema KR, Adejumobi II. Genetic analysis of grain yield and related traits of extra-early orange maize inbred lines and their hybrids under drought and rain-fed conditions. Front Plant Sci. 2024;15:1463924. doi: 10.3389/fpls.2024.1463924 39678008 PMC11637839

[4] Akinwale RO, Badu-Apraku B, Fakorede MAB, Vroh-Bi I. “Heterotic grouping of tropical early-maturing maize inbred lines based on combining ability in Striga-infested and Striga-free environments and the use of SSR markers for genotyping,”. F. Crop. Res. 2014;156.doi: 10.1016/j.fcr.2013.10.015

[5] Badu-Apraku B, Abubakar AM, Adu GB, Yacoubou A-M, Adewale S, Adejumobi II. Enhancing Genetic Gains in Grain Yield and Efficiency of Testing Sites of Early-Maturing Maize Hybrids under Contrasting Environments. Genes (Basel). 2023;14(10):1900. doi: 10.3390/genes14101900 37895251 PMC10606723

[6] Meseka SK, Menkir A, Ibrahim AES, Ajala SO. “Genetic analysis of maize inbred lines for tolerance to drought and low nitrogen,”. Jonares. 2013;1:29–36.

[7] Badu-Apraku B, Fakorede MAB. Advances in genetic enhancement of early and extra-early maize for sub-Saharan Africa. 2017. doi: 10.1007/978-3-319-64852-1

[8] Adu GB, Badu-Apraku B, Akromah R, Garcia-Oliveira AL, Awuku FJ, Gedil M, et al. “Genetic diversity and population structure of early-maturing tropical maize inbred lines using SNP markers,” PLoS One, vol. 14, no. 4, 2019, doi: 10.1371/journal.pone.0214810 PMC6456193 30964890

[9] Botstein D, White RL, Skolnick M, Davis RW. “Construction of a genetic linkage map in man using restriction fragment length polymorphisms”. 1980. PMC1686077 6247908

[10] Vos P, Hogers R, Bleeker M, Reijans M, van de Lee T, Hornes M, et al. AFLP: a new technique for DNA fingerprinting. Nucleic Acids Res. 1995;23(21):4407–14. doi: 10.1093/nar/23.21.4407 7501463 PMC307397

[11] Mueller UG, Wolfenbarger LLR. “AFLP genotyping and fingerprinting,”. 1999. doi: 10.1016/S0169-5347(99)01659-6 10481200

[12] Zhu J, Weir B. “Mixed Model Approaches for Genetic Analysis of Quantitative Traits,”. 1998;973:321–30. Available: https://www.researchgate.net/publication/265369341

[13] Silva DM, Siqueira MVBM, Carrasco NF, Mantello CC, Nascimento WF, Veasey EA. Genetic diversity among air yam (Dioscorea bulbifera) varieties based on single sequence repeat markers. Genet Mol Res. 2016;15(2):10.4238/gmr.15027929. doi: 10.4238/gmr.15027929 27323077

[14] Barata C, Carena MJ. Classification of North Dakota maize inbred lines into heterotic groups based on molecular and testcross data. Euphytica. 2006;151(3):339–49. doi: 10.1007/s10681-006-9155-y

[15] Badu-Apraku B, Garcia-Oliveira AL, Petroli CD, Hearne S, Adewale SA, Gedil M. “Genetic diversity and population structure of early and extra-early maturing maize germplasm adapted to sub-Saharan Africa,” BMC Plant Biol. 2021;21(1).doi: 10.1186/s12870-021-02829-6 PMC7888073 33596835

[16] Mengesha WA, Menkir A, Unakchukwu N, Meseka S, Farinola A, Girma G, Gedil M, et al.“Genetic diversity of tropical maize inbred lines combining resistance to Striga hermonthica with drought tolerance using SNP markers,” Plant Breed., vol. 136, no. 3, 2017, doi: 10.1111/pbr.12479

[17] Kilian A, Sanewski G, Ko L. The application of DArTseq technology to pineapple. Acta Hortic. 2016;(1111):181–8. doi: 10.17660/actahortic.2016.1111.27

[18] Semagn K, Beyene Y, Makumbi D, Mugo S, Prasanna BM, Magorokosho C, et al. Quality control genotyping for assessment of genetic identity and purity in diverse tropical maize inbred lines. Theor Appl Genet. 2012;125(7):1487–501. doi: 10.1007/s00122-012-1928-1 22801872

[19] Frichot E, François O.“LEA: An R package for landscape and ecological association studies,”. Methods Ecol. Evol. 2015;6(8).doi: 10.1111/2041-210X.12382

[20] Lê S, Josse J, Husson F. “FactoMineR: An R package for multivariate analysis”. J. Stat. Softw. 2008;25(1). 2008, doi: 10.18637/jss.v025.i01

[21] Kassambara A, Mundt F. “Package ‘factoextra’: Extract and visualize the results of multivariate data analyses”. CRAN- R Packag. 2020.

[22] Jombart T. “Adegenet: A R package for the multivariate analysis of genetic markers”. Bioinformatics. 2008;24(11). 2008, doi: 10.1093/bioinformatics/btn129 18397895

[23] Chen ZL, et al. “A high-speed search engine pLink 2 with systematic evaluation for proteome-scale identification of cross-linked peptides”. Nat. Commun. 2019;10(1). 2019, doi: 10.1038/s41467-019-11337-z PMC6667459 31363125

[24] Wang J, Zhang Z. “GAPIT Version 3: Boosting Power and Accuracy for Genomic Association and Prediction,” Genomics, Proteomics Bioinforma. 2021;19(4).doi: 10.1016/j.gpb.2021.08.005 PMC9121400 34492338

[25] RC. Team. “A language and environment for statistical computing. R Foundation for Statistical Computing,”. Vienna, Austria; 2017.

[26] Purcell S, Neale B, Todd-Brown K, Thomas L, Ferreira MAR, Bender D, et al. PLINK: a tool set for whole-genome association and population-based linkage analyses. Am J Hum Genet. 2007;81(3):559–75. doi: 10.1086/519795 17701901 PMC1950838

[27] Bradbury PJ, Zhang Z, Kroon DE, Casstevens TM, Ramdoss Y, Buckler ES. TASSEL: software for association mapping of complex traits in diverse samples. Bioinformatics. 2007;23(19):2633–5. doi: 10.1093/bioinformatics/btm308 17586829

[28] Gómez-Rubio V. “ggplot2 - Elegant Graphics for Data Analysis (2nd Edition),” J. Stat. Softw. 2017;vol. 77, no. Book Review 2, pp. 160–7, 2017, doi: 10.18637/jss.v077.b02

[29] Remington DL, Thornsberry JM, Matsuoka Y, Wilson LM, Whitt SR, Doebley J, et al. Structure of linkage disequilibrium and phenotypic associations in the maize genome. Proc Natl Acad Sci U S A. 2001;98(20):11479–84. doi: 10.1073/pnas.201394398 11562485 PMC58755

[30] Yan J, Shah T, Warburton ML, Buckler ES, McMullen MD, Crouch J. “Genetic characterization and linkage disequilibrium estimation of a global maize collection using SNP markers,” PLoS One. 2009;4(12).doi: 10.1371/journal.pone.0008451 PMC2795174 20041112

[31] Jombart T, Devillard S, Balloux F. Discriminant analysis of principal components: a new method for the analysis of genetically structured populations. BMC Genet. 2010;11:94. doi: 10.1186/1471-2156-11-94 20950446 PMC2973851

[32] Paradis E, Schliep K. “Ape 5.0: An environment for modern phylogenetics and evolutionary analyses in R,” Bioinformatics, vol. 35, no. 3, 2019, doi: 10.1093/bioinformatics/bty633 30016406

[33] Yu G, Smith DK, Zhu H, Guan Y, Lam TTY. “ggtree: an r package for visualization and annotation of phylogenetic trees with their covariates and other associated data,” Methods Ecol. Evol. 2017;8(1): 2017, doi: 10.1111/2041-210X.12628

[34] Peakall R, Smouse P. “GenAlEx 6.5: genetic analysis in Excel. Population genetic software for teaching and research-an update.,” Bioinformatics. 2012;28:2537–9. 10.1093/bioinformatics/bts460 PMC3463245 22820204

[35] De Beukelaer H, Davenport G. “Corehunter: multi-purpose core subset selection,” *CRAN*: R package. 2018.doi: 10.32614/CRAN.package.corehunter

[36] Brown AHD. “Core collections: A practical approach to genetic resources management,” in *Genome*. 1989. doi: 10.1139/g89-144

[37] Upadhyaya HD, Ortiz R. “A mini core subset for capturing diversity and promoting utilization of chickpea genetic resources in crop improvement”. Theor Appl Genet. 2001;102(8): 2001.doi: 10.1007/s00122-001-0556-y

[38] R. C. Team. “R Core Team 2023 R: A language and environment for statistical computing. R foundation for statistical computing". R Found Stat Comput. 2023. https://www.R-project.org/

[39] Beyene Y, Semagn K, Mugo S, Tarekegne A, Babu R, Meisel B, et al. “Genetic Gains in Grain Yield Through Genomic Selection in Eight Bi-parental Maize Populations under Drought Stress,”. 2015.doi: 10.2135/cropsci2014.07.0460

[40] Crossa J, Pérez-Rodríguez P, Cuevas J, Montesinos-López O, Jarquín D, De Los Campos G, et al. “Genomic Selection in Plant Breeding: Methods, Models, and Perspectives”. 2017. doi: 10.1016/j.tplants.2017.08.011 28965742

[41] Wu Y, San Vicente F, Huang K, Dhliwayo T, Costich DE, Semagn K, et al. “Molecular characterization of CIMMYT maize inbred lines with genotyping-by-sequencing SNPs”. Theor Appl Genet. 2016;129(4).doi: 10.1007/s00122-016-2664-8 PMC4799255 26849239

[42] Semagn K, Magorokosho C, Vivek BS, Makumbi D, Beyene Y, Mugo S, et al. Molecular characterization of diverse CIMMYT maize inbred lines from eastern and southern Africa using single nucleotide polymorphic markers. BMC Genomics. 2012;13:113. doi: 10.1186/1471-2164-13-113 22443094 PMC3362765

[43] Wang X, Luo G, Yang W, Li Y, Sun J, Zhan K, et al. “Genetic diversity, population structure and marker-trait associations for agronomic and grain traits in wild diploid wheat Triticum urartu,”. BMC Plant Biol. 2017;17(1).doi: 10.1186/s12870-017-1058-7 PMC5494140 28668082

[44] Aguirre-Liguori JA, Ramírez-Barahona S, Tiffin P, Eguiarte LE. Climate change is predicted to disrupt patterns of local adaptation in wild and cultivated maize. Proc Biol Sci. 2019;286(1906):20190486. doi: 10.1098/rspb.2019.0486 31290364 PMC6650710

[45] Bhadmus OA, Badu‐apraku B, Adeyemo OA, Ogunkanmi AL. “Genetic analysis of early white quality protein maize inbreds and derived hybrids under low‐nitrogen and combined drought and heat stress environments,” Plants, vol. 10, no. 12, 2021, doi: 10.3390/plants10122596 PMC8706249 34961067

[46] Lu Y, Yan J, Guimarães CT, Taba S, Hao Z, Gao S, et al. Molecular characterization of global maize breeding germplasm based on genome-wide single nucleotide polymorphisms. Theor Appl Genet. 2009;120(1):93–115. doi: 10.1007/s00122-009-1162-7 19823800

[47] Thachuk C, Crossa J, Franco J, Dreisigacker S, Warburton M, Davenport GF. Core Hunter: an algorithm for sampling genetic resources based on multiple genetic measures. BMC Bioinformatics. 2009;10:243. doi: 10.1186/1471-2105-10-243 19660135 PMC2734557

[48] Odong TL, Jansen J, van Eeuwijk FA, van Hintum TJL. “Quality of core collections for effective utilisation of genetic resources review, discussion and interpretation”. 2013. doi: 10.1007/s00122-012-1971-y PMC3555244 22983567

[49] Isidro J, Jannink J-L, Akdemir D, Poland J, Heslot N, Sorrells ME. Training set optimization under population structure in genomic selection. Theor Appl Genet. 2015;128(1):145–58. doi: 10.1007/s00122-014-2418-4 25367380 PMC4282691

[50] Akdemir D, Sanchez JI, Jannink J-L. Optimization of genomic selection training populations with a genetic algorithm. Genet Sel Evol. 2015;47(1):38. doi: 10.1186/s12711-015-0116-6 25943105 PMC4422310

[51] Akdemir D, Sánchez JI. “Efficient breeding by genomic mating”. Front Genet. 2016;7.doi: 10.3389/fgene.2016.00210 PMC5126051 27965707

[52] Gouesnard B, Bataillon TM, Decoux G, Rozale C, Schoen DJ, David JL. MSTRAT: an algorithm for building germ plasm core collections by maximizing allelic or phenotypic richness. J Hered. 2001;92(1):93–4. doi: 10.1093/jhered/92.1.93 11336240

[53] Bänziger M, Edmeades GO, Beck D, Bellon M. *Breeding for Drought and Nitrogen Stress Tolerance in Maize: From Theory to Practice*. Mexico: CIMMYT, Mexico: 2000.

[54] Cairns JE, Hellin J, Sonder K, Araus JL, MacRobert JF, Thierfelder C, et al. “Adapting maize production to climate change in sub-Saharan Africa,”. 2013. doi: 10.1007/s12571-013-0256-x

[55] Prasanna BM, Cairns JE, Zaidi PH, Beyene Y, Makumbi D, Gowda M, et al. “Beat the stress: breeding for climate resilience in maize for the tropical rainfed environments,”. 2021. doi: 10.1007/s00122-021-03773-7 PMC7885763 33594449

