# Supplementary material for: Genetic diversity and population structure of early-maturing yellow and orange maize inbred lines for improved breeding applications
Source: PLoS One. 2026 Sep 25;21(9):e0358751. doi: 10.1371/journal.pone.0358751 (PMC13614566; doi:10.1371/journal.pone.0358751)

REVIEWER REPORT - Sancar BULUT **22.06.2026**

Manuscript Title: Genetic Diversity and Population Structure of Early-Maturing Yellow and Orange Maize Inbred Lines for Improved Breeding Applications
Manuscript Number: PONE-D-25-49435R2
Authors: Neo Jeremiah Mahula, Idris Ishola Adejumobi, Julius Akinyemi Fagbayide, Baffour Badu-Apraku, John Derera
Journal: PLOS ONE
Reviewer: Sancar BULUT
Round of Review: Second (Re-review)

General Assessment

The authors have provided a thorough, point-by-point response to all reviewer comments and have substantially revised the manuscript accordingly. The revisions are comprehensive and have markedly improved the scientific rigor, methodological transparency, and interpretative caution of the study.

Key improvements include:

- Replacement of overinterpreted claims (e.g., "confirmed heterotic groups") with more cautious terminology such as "putative heterotic groups" and "molecular clusters."
- Significant expansion of the Materials and Methods section, with detailed descriptions of the DArTag genotyping platform, quality control thresholds, population structure analyses (STRUCTURE, DAPC), LD decay estimation, and core set selection algorithms (CoreHunter and greedy stepwise).
- Addition of new analyses for core set validation (allelic richness, He, Ho, Shannon diversity index, mean genetic distance, and PCA-based diversity space coverage), now presented in Table 6 and Figures 8–9.
- Moderation of the LD discussion to avoid unsupported implications regarding QTL detection and GWAS performance, focusing instead on historical recombination patterns.
- Alignment of molecular clustering with pedigree backgrounds without claiming biological validation.

These revisions have addressed the major concerns raised in the first round. The manuscript now meets PLOS ONE’s standards for methodological transparency, statistical reporting, and evidence-based interpretation.

Status of Major Points from the Previous Review of Mine

Criterion	Initial Assessment	Revisions Made	Current Status
Methodological transparency	Insufficient detail for reproducibility	Methods expanded extensively;	**Satisfactory**

Criterion	Initial Assessment	Revisions Made	Current Status
		genotyping platform, filtering thresholds, software parameters, and references added	
Statistical reporting	Inconsistent variability measures; unclear multiple-testing corrections	He, Ho, PIC, MAF clearly reported; AMOVA and FST values provided with significance levels	**Satisfactory** (multiple correction not explicitly stated, but not central to the study's primary objectives)
Results and interpretation	Overstated conclusions; speculation beyond data	Terminology moderated ("putative"); LD and core set discussions now more cautious and data-grounded	**Satisfactory**
Literature context	Insufficient recent references	Recent literature (2021–2024) incorporated	**Satisfactory**
Core set validation	Preservation of diversity not adequately demonstrated	New metrics (allelic richness, He, Ho, Shannon, genetic distance, PCA coverage) and Table 6 + Figures 8–9 added	**Satisfactory**

Specific Minor Issues Identified (Requiring Correction)

While the manuscript is now scientifically sound and largely compliant with PLOS ONE policies, a few minor inconsistencies and typographical errors remain. These should be addressed before final acceptance.

1. **Core set percentage inconsistency:**
    - In the **Abstract**, the authors state: *"A core set representing **15%** of the collection was identified"*.

- However, in the **Materials and Methods** and **Results** sections, the target core size is clearly defined as **20%** of the total panel (76 lines out of 376). Table 6 also confirms that 76 lines were selected.
- **Recommendation:** Correct the Abstract to state **20%** instead of 15%, or alternatively, adjust the selection to 56 lines if 15% was truly intended. Given that all analyses were performed on the 76-line core, the Abstract should be revised to read *"20% of the collection"*.

2. **Missing content in Table 2:**
   - In the provided PDF, Table 2 appears only with its title and column headers; the actual numerical data (He, Ho, MAF, PIC by chromosome) are not visible. I assume the complete table is present in the full submission, but the authors should verify that Table 2 is correctly formatted and fully populated in the final version.
3. **Missing Figure 1:**
   - The text refers to *"Fig. 1 and Table 2"* for the distribution of SNPs across chromosomes, but Figure 1 is not included in the PDF provided for review. This is likely a file upload issue, but the authors should ensure that Figure 1 is properly submitted and referenced.
4. **Typographical errors:**
   - Page 18 (PDF page 65): *"inbreds with < 80 were designated as **admixt**."* Should be corrected to **"admixed"**.
   - Page 16 (PDF page 62): *"International Institute of Tropical Agriculture (IITA). Ibadan."* — The period after "IITA" should be a comma (", Ibadan.") for correct punctuation.
5. **Data Availability Statement wording:**
   - The current statement reads: *"Part of the data on which the conclusions in this article was based have been uploaded as extended data. Others (SNP data) are available in the data repository..."*
   - This phrasing is somewhat vague. PLOS ONE requires all data underlying the findings to be fully and unrestricted available. Since the SNP data are already publicly accessible via Figshare with a DOI, the statement should be simplified and clarified to state directly that all SNP data are available at the Figshare repository, with the DOI provided.

Overall Recommendation

The authors have successfully addressed all substantive concerns raised during the initial review. The manuscript now presents a robust, well-documented genomic characterization of early-maturing maize inbred lines, with appropriate methodological detail, cautious interpretation, and practical relevance for tropical maize breeding programs.

The remaining issues are minor and easily correctable. I therefore recommend: **Minor Revision**

The manuscript should be accepted for publication after the authors correct the core-set percentage inconsistency in the Abstract, fix the typographical errors, clarify the Data Availability Statement, and verify that all tables and figures are complete in the final submission.

Confidential Comments to the Editor

The authors have responded thoroughly and appropriately to all reviewer critiques. The revised manuscript is substantially improved in terms of methodological clarity, statistical transparency, and interpretational balance. The core set validation analyses are particularly well-executed and address the key concern regarding diversity preservation.

In my view, the manuscript now meets PLOS ONE's criteria for scientific validity and reproducibility. The remaining issues are purely minor (a percentage inconsistency in the Abstract, a couple of typos, and a slightly vague data availability statement). I strongly recommend acceptance after minor revisions. No further external review is necessary; the editorial team can verify these corrections.

Comments to the Authors

Dear Authors,

Thank you for your careful and comprehensive responses to the previous round of reviews. Your revisions have significantly strengthened the manuscript, and I appreciate the additional analyses performed for the core set validation.

Before final publication, please address the following minor points:

1. **Abstract correction:** Revise the statement regarding the core set size from "15%" to "20%" to match the Methods, Results, and Table 6, where a 76-line (20%) core is clearly described.
2. **Typographical fixes:**
    - Change "admixt" to "admixed" in the population structure methodology.
    - Correct punctuation: "IITA. Ibadan" → "IITA, Ibadan."
3. **Data Availability Statement:** Please rephrase the statement to state clearly and directly that all SNP data underlying the findings are publicly available at the Figshare repository with the provided DOI, rather than using vague terms like "part of the data" or "extended data."
4. **Verify completeness:** Please confirm that Table 2 and Figure 1 are complete and correctly formatted in your final submission files, as they were not fully visible in the review PDF.

With these minor corrections, I believe your manuscript will be a valuable contribution to the field and suitable for publication in PLOS ONE.

Sincerely,
Prof. Dr. Sancar BULUT
Reviewer, 22.06.2026

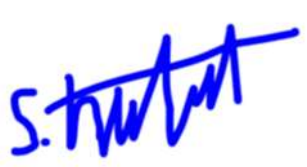

Supplement: Supplementary file 1 [file pone.0358751.s005.pdf]
