# Supplementary material for: Genetic diversity and population structure of early-maturing yellow and orange maize inbred lines for improved breeding applications
Source: PLoS One. 2026 Sep 25;21(9):e0358751. doi: 10.1371/journal.pone.0358751 (PMC13614566; doi:10.1371/journal.pone.0358751)
Supplement: Supplementary file 2 [file pone.0358751.s001.docx]

Supplementary Table S1: List of inbreds that constitute the study population and their pedigree

| Pedigree | Inbreds | Endosperm color |
| --- | --- | --- |
| TZE-Y Pop DT C5 STR C5 | TZEI 2876 | Yellow |
| TZE-Y Pop DT C5 STR C5 | TZEI 2877 | Yellow |
| TZE-Y Pop DT C5 STR C5 | TZEI 2878 | Yellow |
| TZE-Y Pop DT C5 STR C5 | TZEI 2879 | Yellow |
| TZE-Y Pop DT C5 STR C5 | TZEI 2880 | Yellow |
| TZE-Y Pop DT C5 STR C5 | TZEI 2881 | Yellow |
| TZE-Y Pop DT C5 STR C5 | TZEI 2882 | Yellow |
| TZE-Y Pop DT C5 STR C5 | TZEI 2883 | Yellow |
| TZE-Y Pop DT C5 STR C5 | TZEI 2884 | Yellow |
| TZE-Y Pop DT C5 STR C5 | TZEI 2885 | Yellow |
| TZE-Y Pop DT C5 STR C5 | TZEI 2886 | Yellow |
| TZE-Y Pop DT C5 STR C5 | TZEI 2887 | Yellow |
| TZE-Y Pop DT C5 STR C5 | TZEI 2888 | Yellow |
| TZE-Y Pop DT C5 STR C5 | TZEI 2889 | Yellow |
| TZE-Y Pop DT C5 STR C5 | TZEI 2890 | Yellow |
| TZE-Y Pop DT C5 STR C5 | TZEI 2891 | Yellow |
| TZE-Y Pop DT C5 STR C5 | TZEI 2892 | Yellow |
| TZE-Y Pop DT C5 STR C5 | TZEI 2893 | Yellow |
| TZE-Y Pop DT C5 STR C5 | TZEI 2894 | Yellow |
| TZE-Y Pop DT C5 STR C5 | TZEI 2895 | Yellow |
| TZE-Y Pop DT C5 STR C5 | TZEI 2896 | Yellow |
| TZE-Y Pop DT C5 STR C5 | TZEI 2897 | Yellow |
| TZE-Y Pop DT C5 STR C5 | TZEI 2898 | Yellow |
| TZE-Y Pop DT C5 STR C5 | TZEI 2899 | Yellow |
| TZE-Y Pop DT C5 STR C5 | TZEI 2900 | Yellow |
| TZE-Y Pop DT C5 STR C5 | TZEI 2901 | Yellow |
| TZE-Y Pop DT C5 STR C5 | TZEI 2902 | Yellow |
| TZE-Y Pop DT C5 STR C5 | TZEI 2903 | Yellow |
| TZE-Y Pop DT C5 STR C5 | TZEI 2904 | Yellow |
| TZE-Y Pop DT C5 STR C5 | TZEI 2905 | Yellow |
| TZE-Y Pop DT C5 STR C5 | TZEI 2906 | Yellow |
| TZE-Y Pop DT C5 STR C5 | TZEI 2907 | Yellow |
| TZE-Y Pop DT C5 STR C5 | TZEI 2908 | Yellow |
| TZE-Y Pop DT C5 STR C5 | TZEI 2909 | Yellow |
| TZE-Y Pop DT C5 STR C5 | TZEI 2910 | Yellow |
| TZE-Y Pop DT C5 STR C5 | TZEI 2911 | Yellow |
| TZE-Y Pop DT C5 STR C5 | TZEI 2912 | Yellow |
| TZE-Y Pop DT C5 STR C5 | TZEI 2913 | Yellow |
| TZE-Y Pop DT C5 STR C5 | TZEI 2914 | Yellow |
| TZE-Y Pop DT C5 STR C5 | TZEI 2915 | Yellow |
| TZE-Y Pop DT C5 STR C5 | TZEI 2916 | Yellow |
| TZE-Y Pop DT C5 STR C5 | TZEI 2917 | Yellow |
| TZE-Y Pop DT C5 STR C5 | TZEI 2918 | Yellow |
| TZE-Y Pop DT C5 STR C5 | TZEI 2919 | Yellow |
| TZE-Y Pop DT C5 STR C5 | TZEI 2920 | Yellow |
| TZE-Y Pop DT C5 STR C5 | TZEI 2921 | Yellow |
| TZE-Y Pop DT C5 STR C5 | TZEI 2923 | Yellow |
| TZE-Y Pop DT C5 STR C5 | TZEI 2924 | Yellow |
| TZE-Y Pop DT C5 STR C5 | TZEI 2925 | Yellow |
| TZE-Y Pop DT C5 STR C5 | TZEI 2926 | Yellow |
| TZE-Y Pop DT C5 STR C5 | TZEI 2927 | Yellow |
| TZE-Y Pop DT C5 STR C5 | TZEI 2928 | Yellow |
| TZE-Y Pop DT C5 STR C5 | TZEI 2929 | Yellow |
| TZE-Y Pop DT C5 STR C5 | TZEI 2930 | Yellow |
| TZE-Y Pop DT C5 STR C5 | TZEI 2931 | Yellow |
| TZE-Y Pop DT C5 STR C5 | TZEI 2932 | Yellow |
| TZE-Y Pop DT C5 STR C5 | TZEI 2933 | Yellow |
| TZE-Y Pop DT C5 STR C5 | TZEI 2934 | Yellow |
| TZE-Y Pop DT C5 STR C5 | TZEI 2935 | Yellow |
| DTE STR-Y Syn Pop C4 | TZEI 2936 | Yellow |
| DTE STR-Y Syn Pop C4 | TZEI 2937 | Yellow |
| DTE STR-Y Syn Pop C4 | TZEI 2938 | Yellow |
| DTE STR-Y Syn Pop C4 | TZEI 2939 | Yellow |
| DTE STR-Y Syn Pop C4 | TZEI 2940 | Yellow |
| DTE STR-Y Syn Pop C4 | TZEI 2941 | Yellow |
| DTE STR-Y Syn Pop C4 | TZEI 2942 | Yellow |
| DTE STR-Y Syn Pop C4 | TZEI 2943 | Yellow |
| DTE STR-Y Syn Pop C4 | TZEI 2944 | Yellow |
| DTE STR-Y Syn Pop C4 | TZEI 2945 | Yellow |
| DTE STR-Y Syn Pop C4 | TZEI 2946 | Yellow |
| DTE STR-Y Syn Pop C4 | TZEI 2947 | Yellow |
| DTE STR-Y Syn Pop C4 | TZEI 2948 | Yellow |
| DTE STR-Y Syn Pop C4 | TZEI 2949 | Yellow |
| DTE STR-Y Syn Pop C4 | TZEI 2950 | Yellow |
| DTE STR-Y Syn Pop C4 | TZEI 2951 | Yellow |
| DTE STR-Y Syn Pop C4 | TZEI 2952 | Yellow |
| DTE STR-Y Syn Pop C4 | TZEI 2953 | Yellow |
| DTE STR-Y Syn Pop C4 | TZEI 2954 | Yellow |
| DTE STR-Y Syn Pop C4 | TZEI 2955 | Yellow |
| DTE STR-Y Syn Pop C4 | TZEI 2956 | Yellow |
| DTE STR-Y Syn Pop C4 | TZEI 2957 | Yellow |
| DTE STR-Y Syn Pop C4 | TZEI 2958 | Yellow |
| DTE STR-Y Syn Pop C4 | TZEI 2959 | Yellow |
| DTE STR-Y Syn Pop C4 | TZEI 2960 | Yellow |
| DTE STR-Y Syn Pop C4 | TZEI 2961 | Yellow |
| DTE STR-Y Syn Pop C4 | TZEI 2962 | Yellow |
| DTE STR-Y Syn Pop C4 | TZEI 2963 | Yellow |
| DTE STR-Y Syn Pop C4 | TZEI 2964 | Yellow |
| DTE STR-Y Syn Pop C4 | TZEI 2965 | Yellow |
| DTE STR-Y Syn Pop C4 | TZEI 2966 | Yellow |
| DTE STR-Y Syn Pop C4 | TZEI 2967 | Yellow |
| DTE STR-Y Syn Pop C4 | TZEI 2968 | Yellow |
| DTE STR-Y Syn Pop C4 | TZEI 2969 | Yellow |
| DTE STR-Y Syn Pop C4 | TZEI 2970 | Yellow |
| DTE STR-Y Syn Pop C4 | TZEI 2971 | Yellow |
| DTE STR-Y Syn Pop C4 | TZEI 2972 | Yellow |
| DTE STR-Y Syn Pop C4 | TZEI 2973 | Yellow |
| DTE STR-Y Syn Pop C4 | TZEI 2974 | Yellow |
| DTE STR-Y Syn Pop C4 | TZEI 2975 | Yellow |
| DTE STR-Y Syn Pop C4 | TZEI 2977 | Yellow |
| DTE STR-Y Syn Pop C4 | TZEI 2978 | Yellow |
| DTE STR-Y Syn Pop C4 | TZEI 2979 | Yellow |
| DTE STR-Y Syn Pop C4 | TZEI 2980 | Yellow |
| DTE STR-Y Syn Pop C4 | TZEI 2981 | Yellow |
| DTE STR-Y Syn Pop C4 | TZEI 2982 | Yellow |
| DTE STR-Y Syn Pop C4 | TZEI 2983 | Yellow |
| DTE STR-Y Syn Pop C4 | TZEI 2984 | Yellow |
| DTE STR-Y Syn Pop C4 | TZEI 2985 | Yellow |
| DTE STR-Y Syn Pop C4 | TZEI 2986 | Yellow |
| DTE STR-Y Syn Pop C4 | TZEI 2987 | Yellow |
| DTE STR-Y Syn Pop C4 | TZEI 2988 | Yellow |
| DTE STR-Y Syn Pop C4 | TZEI 2989 | Yellow |
| DTE STR-Y Syn Pop C4 | TZEI 2990 | Yellow |
| DTE STR-Y Syn Pop C4 | TZEI 2991 | Yellow |
| DTE STR-Y Syn Pop C4 | TZEI 2992 | Yellow |
| DTE STR-Y Syn Pop C4 | TZEI 2993 | Yellow |
| DTE STR-Y Syn Pop C4 | TZEI 2994 | Yellow |
| DTE STR-Y Syn Pop C4 | TZEI 2995 | Yellow |
| DTE STR-Y Syn Pop C4 | TZEI 2996 | Yellow |
| DTE STR-Y Syn Pop C4 | TZEI 2997 | Yellow |
| DTE STR-Y Syn Pop C4 | TZEI 2998 | Yellow |
| DTE STR-Y Syn Pop C4 | TZEI 2999 | Yellow |
| DTE STR-Y Syn Pop C4 | TZEI 3000 | Yellow |
| DTE STR-Y Syn Pop C4 | TZEI 3001 | Yellow |
| DTE STR-Y Syn Pop C4 | TZEI 3002 | Yellow |
| DTE STR-Y Syn Pop C4 | TZEI 3003 | Yellow |
| DTE STR-Y Syn Pop C4 | TZEI 3004 | Yellow |
| DTE STR-Y Syn Pop C4 | TZEI 3005 | Yellow |
| TZE-Y Pop DT C5 STR C5 | TZEIOR 204 | Orange |
| TZE-Y Pop DT C5 STR C5 | TZEIOR 205 | Orange |
| TZE-Y Pop DT C5 STR C5 | TZEIOR 206 | Orange |
| TZE-Y Pop DT C5 STR C5 | TZEIOR 207 | Orange |
| TZE-Y Pop DT C5 STR C5 | TZEIOR 208 | Orange |
| TZE-Y Pop DT C5 STR C5 | TZEIOR 209 | Orange |
| TZE-Y Pop DT C5 STR C5 | TZEIOR 210 | Orange |
| TZE-Y Pop DT C5 STR C5 | TZEIOR 211 | Orange |
| TZE-Y Pop DT C5 STR C5 | TZEIOR 212 | Orange |
| TZE-Y Pop DT C5 STR C5 | TZEIOR 213 | Orange |
| TZE-Y Pop DT C5 STR C5 | TZEIOR 214 | Orange |
| TZE-Y Pop DT C5 STR C5 | TZEIOR 215 | Orange |
| TZE-Y Pop DT C5 STR C5 | TZEIOR 216 | Orange |
| TZE-Y Pop DT C5 STR C5 | TZEIOR 217 | Orange |
| TZE-Y Pop DT C5 STR C5 | TZEIOR 218 | Orange |
| TZE-Y Pop DT C5 STR C5 | TZEIOR 219 | Orange |
| TZE-Y Pop DT C5 STR C5 | TZEIOR 220 | Orange |
| TZE-Y Pop DT C5 STR C5 | TZEIOR 221 | Orange |
| TZE-Y Pop DT C5 STR C5 | TZEIOR 222 | Orange |
| TZE-Y Pop DT C5 STR C5 | TZEIOR 223 | Orange |
| TZE-Y Pop DT C5 STR C5 | TZEIOR 224 | Orange |
| TZE-Y Pop DT C5 STR C5 | TZEIOR 225 | Orange |
| TZE-Y Pop DT C5 STR C5 | TZEIOR 226 | Orange |
| TZE-Y Pop DT C5 STR C5 | TZEIOR 227 | Orange |
| TZE-Y Pop DT C5 STR C5 | TZEIOR 228 | Orange |
| TZE-Y Pop DT C5 STR C5 | TZEIOR 229 | Orange |
| TZE-Y Pop DT C5 STR C5 | TZEIOR 230 | Orange |
| TZE-Y Pop DT C5 STR C5 | TZEIOR 231 | Orange |
| TZE-Y Pop DT C5 STR C5 | TZEIOR 232 | Orange |
| TZE-Y Pop DT C5 STR C5 | TZEIOR 233 | Orange |
| TZE-Y Pop DT C5 STR C5 | TZEIOR 234 | Orange |
| TZE-Y Pop DT C5 STR C5 | TZEIOR 235 | Orange |
| TZE-Y Pop DT C5 STR C5 | TZEIOR 236 | Orange |
| TZE-Y Pop DT C5 STR C5 | TZEIOR 237 | Orange |
| TZE-Y Pop DT C5 STR C5 | TZEIOR 238 | Orange |
| TZE-Y Pop DT C5 STR C5 | TZEIOR 240 | Orange |
| TZE-Y Pop DT C5 STR C5 | TZEIOR 241 | Orange |
| TZE-Y Pop DT C5 STR C5 | TZEIOR 242 | Orange |
| TZE-Y Pop DT C5 STR C5 | TZEIOR 243 | Orange |
| TZE-Y Pop DT C5 STR C5 | TZEIOR 244 | Orange |
| TZE-Y Pop DT C5 STR C5 | TZEIOR 245 | Orange |
| TZE-Y Pop DT C5 STR C5 | TZEIOR 246 | Orange |
| TZE-Y Pop DT C5 STR C5 | TZEIOR 247 | Orange |
| TZE-Y Pop DT C5 STR C5 | TZEIOR 248 | Orange |
| TZE-Y Pop DT C5 STR C5 | TZEIOR 249 | Orange |
| TZE-Y Pop DT C5 STR C5 | TZEIOR 250 | Orange |
| TZE-Y Pop DT C5 STR C5 | TZEIOR 251 | Orange |
| TZE-Y Pop DT C5 STR C5 | TZEIOR 252 | Orange |
| TZE-Y Pop DT C5 STR C5 | TZEIOR 253 | Orange |
| TZE-Y Pop DT C5 STR C5 | TZEIOR 254 | Orange |
| TZE-Y Pop DT C5 STR C5 | TZEIOR 255 | Orange |
| TZE-Y Pop DT C5 STR C5 | TZEIOR 256 | Orange |
| TZE-Y Pop DT C5 STR C5 | TZEIOR 257 | Orange |
| TZE-Y Pop DT C5 STR C5 | TZEIOR 258 | Orange |
| TZE-Y Pop DT C5 STR C5 | TZEIOR 259 | Orange |
| TZE-Y Pop DT C5 STR C5 | TZEIOR 260 | Orange |
| TZE-Y Pop DT C5 STR C5 | TZEIOR 261 | Orange |
| TZE-Y Pop DT C5 STR C5 | TZEIOR 262 | Orange |
| TZE-Y Pop DT C5 STR C5 | TZEIOR 263 | Orange |
| TZE-Y Pop DT C5 STR C5 | TZEIOR 264 | Orange |
| TZE-Y Pop DT C5 STR C5 | TZEIOR 265 | Orange |
| TZE-Y Pop DT C5 STR C5 | TZEIOR 266 | Orange |
| TZE-Y Pop DT C5 STR C5 | TZEIOR 267 | Orange |
| TZE-Y Pop DT C5 STR C5 | TZEIOR 268 | Orange |
| TZE-Y Pop DT C5 STR C5 | TZEIOR 269 | Orange |
| TZE-Y Pop DT C5 STR C5 | TZEIOR 270 | Orange |
| TZE-Y Pop DT C5 STR C5 | TZEIOR 271 | Orange |
| TZE-Y Pop DT C5 STR C5 | TZEIOR 272 | Orange |
| TZE-Y Pop DT C5 STR C5 | TZEIOR 273 | Orange |
| TZE-Y Pop DT C5 STR C5 | TZEIOR 274 | Orange |
| TZE-Y Pop DT C5 STR C5 | TZEIOR 275 | Orange |
| TZE-Y Pop DT C5 STR C5 | TZEIOR 276 | Orange |
| TZE-Y Pop DT C5 STR C5 | TZEIOR 277 | Orange |
| TZE-Y Pop DT C5 STR C5 | TZEIOR 278 | Orange |
| TZE-Y Pop DT C5 STR C5 | TZEIOR 279 | Orange |
| TZE-Y Pop DT C5 STR C5 | TZEIOR 280 | Orange |
| TZE-Y Pop DT C5 STR C5 | TZEIOR 281 | Orange |
| DTE STR-Y Syn Pop C4 | TZEIOR 364 | Orange |
| DTE STR-Y Syn Pop C4 | TZEIOR 367 | Orange |
| DTE STR-Y Syn Pop C4 | TZEIOR 368 | Orange |
| DTE STR-Y Syn Pop C4 | TZEIOR 369 | Orange |
| DTE STR-Y Syn Pop C4 | TZEIOR 370 | Orange |
| DTE STR-Y Syn Pop C4 | TZEIOR 371 | Orange |
| DTE STR-Y Syn Pop C4 | TZEIOR 373 | Orange |
| DTE STR-Y Syn Pop C4 | TZEIOR 374 | Orange |
| DTE STR-Y Syn Pop C4 | TZEIOR 376 | Orange |
| DTE STR-Y Syn Pop C4 | TZEIOR 377 | Orange |
| DTE STR-Y Syn Pop C4 | TZEIOR 378 | Orange |
| DTE STR-Y Syn Pop C4 | TZEIOR 379 | Orange |
| DTE STR-Y Syn Pop C4 | TZEIOR 380 | Orange |
| DTE STR-Y Syn Pop C4 | TZEIOR 381 | Orange |
| DTE STR-Y Syn Pop C4 | TZEIOR 382 | Orange |
| DTE STR-Y Syn Pop C4 | TZEIOR 384 | Orange |
| DTE STR-Y Syn Pop C4 | TZEIOR 385 | Orange |
| DTE STR-Y Syn Pop C4 | TZEIOR 386 | Orange |
| DTE STR-Y Syn Pop C4 | TZEIOR 387 | Orange |
| DTE STR-Y Syn Pop C4 | TZEIOR 388 | Orange |
| DTE STR-Y Syn Pop C4 | TZEIOR 389 | Orange |
| DTE STR-Y Syn Pop C4 | TZEIOR 390 | Orange |
| DTE STR-Y Syn Pop C4 | TZEIOR 391 | Orange |
| DTE STR-Y Syn Pop C4 | TZEIOR 392 | Orange |
| DTE STR-Y Syn Pop C4 | TZEIOR 393 | Orange |
| DTE STR-Y Syn Pop C4 | TZEIOR 395 | Orange |
| DTE STR-Y Syn Pop C4 | TZEIOR 396 | Orange |
| DTE STR-Y Syn Pop C4 | TZEIOR 397 | Orange |
| DTE STR-Y Syn Pop C4 | TZEIOR 398 | Orange |
| DTE STR-Y Syn Pop C4 | TZEIOR 399 | Orange |
| DTE STR-Y Syn Pop C4 | TZEIOR 400 | Orange |
| DTE STR-Y Syn Pop C4 | TZEIOR 401 | Orange |
| DTE STR-Y Syn Pop C4 | TZEIOR 402 | Orange |
| DTE STR-Y Syn Pop C4 | TZEIOR 403 | Orange |
| DTE STR-Y Syn Pop C4 | TZEIOR 405 | Orange |
| DTE STR-Y Syn Pop C4 | TZEIOR 406 | Orange |
| DTE STR-Y Syn Pop C4 | TZEIOR 407 | Orange |
| DTE STR-Y Syn Pop C4 | TZEIOR 408 | Orange |
| DTE STR-Y Syn Pop C4 | TZEIOR 409 | Orange |
| DTE STR-Y Syn Pop C4 | TZEIOR 410 | Orange |
| DTE STR-Y Syn Pop C4 | TZEIOR 411 | Orange |
| DTE STR-Y Syn Pop C4 | TZEIOR 412 | Orange |
| DTE STR-Y Syn Pop C4 | TZEIOR 413 | Orange |
| DTE STR-Y Syn Pop C4 | TZEIOR 414 | Orange |
| DTE STR-Y Syn Pop C4 | TZEIOR 415 | Orange |
| DTE STR-Y Syn Pop C4 | TZEIOR 416 | Orange |
| DTE STR-Y Syn Pop C4 | TZEIOR 417 | Orange |
| DTE STR-Y Syn Pop C4 | TZEIOR 418 | Orange |
| DTE STR-Y Syn Pop C4 | TZEIOR 419 | Orange |
| DTE STR-Y Syn Pop C4 | TZEIOR 420 | Orange |
| DTE STR-Y Syn Pop C4 | TZEIOR 421 | Orange |
| DTE STR-Y Syn Pop C4 | TZEIOR 422 | Orange |
| DTE STR-Y Syn Pop C4 | TZEIOR 423 | Orange |
| TZEIOR 9 x TZEIOR 56 | TZEIOR 468 | Orange |
| TZEIOR 9 x TZEIOR 56 | TZEIOR 469 | Orange |
| TZEIOR 9 x TZEIOR 56 | TZEIOR 470 | Orange |
| TZEIOR 9 x TZEIOR 56 | TZEIOR 471 | Orange |
| TZEIOR 9 x TZEIOR 56 | TZEIOR 472 | Orange |
| TZEIOR 9 x TZEIOR 56 | TZEIOR 473 | Orange |
| TZEIOR 9 x TZEIOR 56 | TZEIOR 474 | Orange |
| TZEIOR 9 x TZEIOR 56 | TZEIOR 475 | Orange |
| TZEIOR 9 x TZEIOR 56 | TZEIOR 476 | Orange |
| TZEIOR 9 x TZEIOR 56 | TZEIOR 477 | Orange |
| TZEIOR 9 x TZEIOR 56 | TZEIOR 478 | Orange |
| TZEIOR 9 x TZEIOR 56 | TZEIOR 479 | Orange |
| TZEIOR 9 x TZEIOR 56 | TZEIOR 480 | Orange |
| TZEIOR 9 x TZEIOR 56 | TZEIOR 481 | Orange |
| TZEIOR 9 x TZEIOR 56 | TZEIOR 482 | Orange |
| TZEIOR 9 x TZEIOR 56 | TZEIOR 483 | Orange |
| TZEIOR 9 x TZEIOR 56 | TZEIOR 484 | Orange |
| TZEIOR 9 x TZEIOR 56 | TZEIOR 485 | Orange |
| TZEIOR 9 x TZEIOR 56 | TZEIOR 486 | Orange |
| TZEIOR 9 x TZEIOR 56 | TZEIOR 487 | Orange |
| TZEIOR 9 x TZEIOR 56 | TZEIOR 488 | Orange |
| TZEIOR 9 x TZEIOR 56 | TZEIOR 489 | Orange |
| TZEIOR 9 x TZEIOR 56 | TZEIOR 490 | Orange |
| TZEIOR 9 x TZEIOR 56 | TZEIOR 491 | Orange |
| TZEIOR 9 x TZEIOR 56 | TZEIOR 492 | Orange |
| TZEIOR 9 x TZEIOR 56 | TZEIOR 493 | Orange |
| TZEIOR 9 x TZEIOR 56 | TZEIOR 494 | Orange |
| TZEIOR 9 x TZEIOR 56 | TZEIOR 495 | Orange |
| TZEIOR 9 x TZEIOR 56 | TZEIOR 496 | Orange |
| TZEIOR 9 x TZEIOR 56 | TZEIOR 497 | Orange |
| TZEIOR 9 x TZEIOR 56 | TZEIOR 498 | Orange |
| TZEIOR 9 x TZEIOR 56 | TZEIOR 499 | Orange |
| TZEIOR 9 x TZEIOR 56 | TZEIOR 500 | Orange |
| TZEIOR 9 x TZEIOR 56 | TZEIOR 501 | Orange |
| TZEIOR 9 x TZEIOR 56 | TZEIOR 503 | Orange |
| TZEIOR 9 x TZEIOR 56 | TZEIOR 506 | Orange |
| TZEIOR 9 x TZEIOR 56 | TZEIOR 507 | Orange |
| TZEIOR 9 x TZEIOR 56 | TZEIOR 508 | Orange |
| TZEIOR 9 x TZEIOR 56 | TZEIOR 509 | Orange |
| TZEIOR 9 x TZEIOR 56 | TZEIOR 510 | Orange |
| TZEIOR 9 x TZEIOR 56 | TZEIOR 511 | Orange |
| TZEIOR 9 x TZEIOR 56 | TZEIOR 512 | Orange |
| TZEIOR 9 x TZEIOR 56 | TZEIOR 513 | Orange |
| TZEIOR 9 x TZEIOR 56 | TZEIOR 514 | Orange |
| TZEIOR 9 x TZEIOR 56 | TZEIOR 515 | Orange |
| TZEIOR 9 x TZEIOR 56 | TZEIOR 516 | Orange |
| TZEIOR 9 x TZEIOR 56 | TZEIOR 517 | Orange |
| TZEIOR 9 x TZEIOR 56 | TZEIOR 518 | Orange |
| TZEIOR 9 x TZEIOR 56 | TZEIOR 519 | Orange |
| TZEIOR 9 x TZEIOR 56 | TZEIOR 520 | Orange |
| TZEIOR 9 x TZEIOR 56 | TZEIOR 521 | Orange |
| TZEIOR 9 x TZEIOR 56 | TZEIOR 522 | Orange |
| TZEIOR 9 x TZEIOR 56 | TZEIOR 523 | Orange |
| TZEIOR 9 x TZEIOR 56 | TZEIOR 524 | Orange |
| TZEIOR 9 x TZEIOR 56 | TZEIOR 525 | Orange |
| TZEIOR 9 x TZEIOR 56 | TZEIOR 526 | Orange |
| TZEIOR 9 x TZEIOR 56 | TZEIOR 527 | Orange |
| TZEIOR 9 x TZEIOR 56 | TZEIOR 528 | Orange |
| TZEIOR 9 x TZEIOR 56 | TZEIOR 529 | Orange |
| TZEIOR 9 x TZEIOR 56 | TZEIOR 530 | Orange |
| TZEIOR 9 x TZEIOR 56 | TZEIOR 531 | Orange |
| TZEIOR 9 x TZEIOR 56 | TZEIOR 532 | Orange |
| TZEIOR 9 x TZEIOR 108 | TZEIOR 640 | Orange |
| TZEIOR 9 x TZEIOR 108 | TZEIOR 641 | Orange |
| TZEIOR 9 x TZEIOR 108 | TZEIOR 642 | Orange |
| TZEIOR 9 x TZEIOR 108 | TZEIOR 643 | Orange |
| TZEIOR 9 x TZEIOR 108 | TZEIOR 644 | Orange |
| TZEIOR 9 x TZEIOR 108 | TZEIOR 645 | Orange |
| TZEIOR 9 x TZEIOR 108 | TZEIOR 646 | Orange |
| TZEIOR 9 x TZEIOR 108 | TZEIOR 647 | Orange |
| TZEIOR 9 x TZEIOR 108 | TZEIOR 648 | Orange |
| TZEIOR 9 x TZEIOR 108 | TZEIOR 649 | Orange |
| TZEIOR 9 x TZEIOR 108 | TZEIOR 650 | Orange |
| TZEIOR 9 x TZEIOR 108 | TZEIOR 651 | Orange |
| TZEIOR 9 x TZEIOR 108 | TZEIOR 653 | Orange |
| TZEIOR 9 x TZEIOR 108 | TZEIOR 656 | Orange |
| TZEIOR 9 x TZEIOR 108 | TZEIOR 657 | Orange |
| TZEIOR 9 x TZEIOR 108 | TZEIOR 658 | Orange |
| TZEIOR 9 x TZEIOR 108 | TZEIOR 659 | Orange |
| TZEIOR 9 x TZEIOR 108 | TZEIOR 660 | Orange |
| TZEIOR 9 x TZEIOR 108 | TZEIOR 661 | Orange |
| TZEIOR 9 x TZEIOR 108 | TZEIOR 662 | Orange |
| TZEIOR 9 x TZEIOR 108 | TZEIOR 663 | Orange |
| TZEIOR 9 x TZEIOR 108 | TZEIOR 664 | Orange |
| TZEIOR 9 x TZEIOR 108 | TZEIOR 665 | Orange |
| TZEIOR 9 x TZEIOR 108 | TZEIOR 666 | Orange |
| TZEIOR 9 x TZEIOR 108 | TZEIOR 667 | Orange |
| TZEIOR 9 x TZEIOR 108 | TZEIOR 668 | Orange |
| TZEIOR 9 x TZEIOR 108 | TZEIOR 669 | Orange |
| TZEIOR 9 x TZEIOR 108 | TZEIOR 670 | Orange |
| TZEIOR 9 x TZEIOR 108 | TZEIOR 671 | Orange |
| TZEIOR 9 x TZEIOR 108 | TZEIOR 672 | Orange |
| TZEIOR 9 x TZEIOR 108 | TZEIOR 673 | Orange |
| TZEIOR 9 x TZEIOR 108 | TZEIOR 674 | Orange |
| TZEIOR 9 x TZEIOR 108 | TZEIOR 675 | Orange |
| TZEIOR 9 x TZEIOR 108 | TZEIOR 676 | Orange |
| TZEIOR 9 x TZEIOR 108 | TZEIOR 677 | Orange |
| TZEIOR 9 x TZEIOR 108 | TZEIOR 678 | Orange |
| TZEIOR 9 x TZEIOR 108 | TZEIOR 679 | Orange |
| TZEIOR 9 x TZEIOR 108 | TZEIOR 680 | Orange |
| TZEIOR 9 x TZEIOR 108 | TZEIOR 681 | Orange |
| TZEIOR 9 x TZEIOR 108 | TZEIOR 682 | Orange |
| TZEIOR 9 x TZEIOR 108 | TZEIOR 683 | Orange |
| TZEIOR 9 x TZEIOR 108 | TZEIOR 684 | Orange |
| TZEIOR 9 x TZEIOR 108 | TZEIOR 685 | Orange |
| TZEIOR 9 x TZEIOR 108 | TZEIOR 686 | Orange |
| TZEIOR 9 x TZEIOR 108 | TZEIOR 687 | Orange |
| TZEIOR 9 x TZEIOR 108 | TZEIOR 688 | Orange |
| TZEIOR 9 x TZEIOR 108 | TZEIOR 689 | Orange |
