# Supplementary material for: Genetic diversity and population structure of early-maturing yellow and orange maize inbred lines for improved breeding applications
Source: PLoS One. 2026 Sep 25;21(9):e0358751. doi: 10.1371/journal.pone.0358751 (PMC13614566; doi:10.1371/journal.pone.0358751)
Supplement: Supplementary file 3 [file pone.0358751.s002.docx]

Supplementary Table S2: Population stratification based on the Bayesian statistics approach implemented in STRUCTURE revealed two genetic groups with 5% level of admixt.

| Geno | Q1 | Q2 | Cluster |
| --- | --- | --- | --- |
| TZEI 2972 | 0 795 | 0 205 | 1 |
| TZEI 2880 | 0 799 | 0 201 | 1 |
| TZEIOR 403 | 0 8 | 0 2 | 1 |
| TZEI 2930 | 0 801 | 0 199 | 1 |
| TZEI 2923 | 0 802 | 0 198 | 1 |
| TZEI 2905 | 0 805 | 0 195 | 1 |
| TZEIOR 380 | 0 805 | 0 195 | 1 |
| TZEIOR 479 | 0 89 | 0 191 | 1 |
| TZEIOR 221 | 0 81 | 0 19 | 1 |
| TZEI 2968 | 0 814 | 0 186 | 1 |
| TZEI 2933 | 0 816 | 0 184 | 1 |
| TZEI 2935 | 0 816 | 0 184 | 1 |
| TZEIOR 232 | 0 816 | 0 184 | 1 |
| TZEI 2995 | 0 819 | 0 181 | 1 |
| TZEIOR 265 | 0 823 | 0 177 | 1 |
| TZEI 2918 | 0 825 | 0 175 | 1 |
| TZEI 2963 | 0 827 | 0 173 | 1 |
| TZEI 2980 | 0 827 | 0 173 | 1 |
| TZEIOR 248 | 0 828 | 0 172 | 1 |
| TZEI 2954 | 0 828 | 0 172 | 1 |
| TZEI 2966 | 0 829 | 0 171 | 1 |
| TZEIOR 222 | 0 83 | 0 17 | 1 |
| TZEIOR 480 | 0 83 | 0 17 | 1 |
| TZEIOR 251 | 0 831 | 0 169 | 1 |
| TZEI 3001 | 0 831 | 0 169 | 1 |
| TZEIOR 227 | 0 832 | 0 168 | 1 |
| TZEI 2950 | 0 832 | 0 168 | 1 |
| TZEIOR 415 | 0 837 | 0 163 | 1 |
| TZEIOR 215 | 0 839 | 0 161 | 1 |
| TZEIOR 370 | 0 84 | 0 16 | 1 |
| TZEI 2917 | 0 841 | 0 159 | 1 |
| TZEIOR 386 | 0 841 | 0 159 | 1 |
| TZEI 2883 | 0 843 | 0 157 | 1 |
| TZEIOR 469 | 0 843 | 0 157 | 1 |
| TZEI 2906 | 0 845 | 0 155 | 1 |
| TZEIOR 392 | 0 847 | 0 153 | 1 |
| TZEI 2978 | 0 848 | 0 152 | 1 |
| TZEI 2932 | 0 851 | 0 149 | 1 |
| TZEIOR 390 | 0 852 | 0 148 | 1 |
| TZEI 2908 | 0 853 | 0 147 | 1 |
| TZEIOR 406 | 0 853 | 0 147 | 1 |
| TZEI 2894 | 0 854 | 0 146 | 1 |
| TZEI 2920 | 0 856 | 0 144 | 1 |
| TZEIOR 379 | 0 856 | 0 144 | 1 |
| TZEIOR 212 | 0 857 | 0 143 | 1 |
| TZEIOR 364 | 0 857 | 0 143 | 1 |
| TZEIOR 238 | 0 858 | 0 142 | 1 |
| TZEIOR 255 | 0 858 | 0 142 | 1 |
| TZEIOR 420 | 0 858 | 0 142 | 1 |
| TZEI 2892 | 0 859 | 0 141 | 1 |
| TZEI 2886 | 0 86 | 0 14 | 1 |
| TZEIOR 397 | 0 861 | 0 139 | 1 |
| TZEIOR 483 | 0 862 | 0 138 | 1 |
| TZEI 2909 | 0 863 | 0 137 | 1 |
| TZEIOR 219 | 0 863 | 0 137 | 1 |
| TZEI 2938 | 0 863 | 0 137 | 1 |
| TZEI 2961 | 0 865 | 0 135 | 1 |
| TZEIOR 277 | 0 867 | 0 133 | 1 |
| TZEI 2979 | 0 867 | 0 133 | 1 |
| TZEIOR 409 | 0 868 | 0 132 | 1 |
| TZEIOR 272 | 0 872 | 0 128 | 1 |
| TZEIOR 241 | 0 873 | 0 127 | 1 |
| TZEIOR 278 | 0 873 | 0 127 | 1 |
| TZEIOR 475 | 0 874 | 0 126 | 1 |
| TZEIOR 207 | 0 875 | 0 125 | 1 |
| TZEIOR 281 | 0 876 | 0 124 | 1 |
| TZEIOR 484 | 0 876 | 0 124 | 1 |
| TZEIOR 242 | 0 877 | 0 123 | 1 |
| TZEIOR 267 | 0 877 | 0 123 | 1 |
| TZEI 2915 | 0 878 | 0 122 | 1 |
| TZEIOR 237 | 0 878 | 0 122 | 1 |
| TZEI 2952 | 0 878 | 0 122 | 1 |
| TZEIOR 393 | 0 878 | 0 122 | 1 |
| TZEI 2887 | 0 88 | 0 12 | 1 |
| TZEIOR 231 | 0 88 | 0 12 | 1 |
| TZEI 2903 | 0 881 | 0 119 | 1 |
| TZEIOR 236 | 0 881 | 0 119 | 1 |
| TZEIOR 204 | 0 882 | 0 118 | 1 |
| TZEIOR 250 | 0 882 | 0 118 | 1 |
| TZEIOR 266 | 0 882 | 0 118 | 1 |
| TZEI 2951 | 0 882 | 0 118 | 1 |
| TZEI 2944 | 0 883 | 0 117 | 1 |
| TZEI 2891 | 0 887 | 0 113 | 1 |
| TZEI 2945 | 0 887 | 0 113 | 1 |
| TZEI 2989 | 0 887 | 0 113 | 1 |
| TZEIOR 240 | 0 888 | 0 112 | 1 |
| TZEI 2999 | 0 888 | 0 112 | 1 |
| TZEIOR 422 | 0 888 | 0 112 | 1 |
| TZEI 2956 | 0 889 | 0 111 | 1 |
| TZEIOR 216 | 0 89 | 0 11 | 1 |
| TZEIOR 274 | 0 89 | 0 11 | 1 |
| TZEI 2893 | 0 891 | 0 109 | 1 |
| TZEI 2939 | 0 891 | 0 109 | 1 |
| TZEIOR 371 | 0 891 | 0 109 | 1 |
| TZEIOR 261 | 0 892 | 0 108 | 1 |
| TZEIOR 276 | 0 892 | 0 108 | 1 |
| TZEI 2974 | 0 893 | 0 107 | 1 |
| TZEI 2975 | 0 893 | 0 107 | 1 |
| TZEI 2895 | 0 895 | 0 105 | 1 |
| TZEI 2928 | 0 898 | 0 102 | 1 |
| TZEI 2882 | 0 899 | 0 101 | 1 |
| TZEIOR 254 | 0 899 | 0 101 | 1 |
| TZEI 2924 | 0 9 | 0 1 | 1 |
| TZEIOR 205 | 0 901 | 0 099 | 1 |
| TZEIOR 217 | 0 901 | 0 099 | 1 |
| TZEI 2993 | 0 901 | 0 099 | 1 |
| TZEIOR 480 | 0 92 | 0 098 | 1 |
| TZEIOR 208 | 0 902 | 0 098 | 1 |
| TZEIOR 220 | 0 902 | 0 098 | 1 |
| TZEIOR 209 | 0 903 | 0 097 | 1 |
| TZEIOR 367 | 0 903 | 0 097 | 1 |
| TZEI 2985 | 0 904 | 0 096 | 1 |
| TZEI 2986 | 0 904 | 0 096 | 1 |
| TZEI 2884 | 0 905 | 0 095 | 1 |
| TZEIOR 275 | 0 905 | 0 095 | 1 |
| TZEIOR 252 | 0 906 | 0 094 | 1 |
| TZEI 2896 | 0 907 | 0 093 | 1 |
| TZEIOR 234 | 0 908 | 0 092 | 1 |
| TZEIOR 262 | 0 908 | 0 092 | 1 |
| TZEIOR 381 | 0 908 | 0 092 | 1 |
| TZEI 2955 | 0 909 | 0 091 | 1 |
| TZEIOR 273 | 0 911 | 0 089 | 1 |
| TZEIOR 279 | 0 911 | 0 089 | 1 |
| TZEI 2964 | 0 911 | 0 089 | 1 |
| TZEIOR 405 | 0 913 | 0 087 | 1 |
| TZEIOR 470 | 0 913 | 0 087 | 1 |
| TZEI 2898 | 0 917 | 0 083 | 1 |
| TZEI 2904 | 0 917 | 0 083 | 1 |
| TZEIOR 213 | 0 917 | 0 083 | 1 |
| TZEIOR 224 | 0 917 | 0 083 | 1 |
| TZEI 2973 | 0 917 | 0 083 | 1 |
| TZEIOR 410 | 0 918 | 0 082 | 1 |
| TZEIOR 223 | 0 919 | 0 081 | 1 |
| TZEI 2942 | 0 92 | 0 08 | 1 |
| TZEI 2899 | 0 921 | 0 079 | 1 |
| TZEI 2919 | 0 921 | 0 079 | 1 |
| TZEI 2988 | 0 921 | 0 079 | 1 |
| TZEI 2967 | 0 922 | 0 078 | 1 |
| TZEIOR 416 | 0 922 | 0 078 | 1 |
| TZEIOR 249 | 0 928 | 0 072 | 1 |
| TZEIOR 256 | 0 928 | 0 072 | 1 |
| TZEI 3000 | 0 928 | 0 072 | 1 |
| TZEIOR 421 | 0 928 | 0 072 | 1 |
| TZEIOR 269 | 0 929 | 0 071 | 1 |
| TZEI 2984B | 0 929 | 0 071 | 1 |
| TZEIOR 235 | 0 93 | 0 07 | 1 |
| TZEI 2992 | 0 931 | 0 069 | 1 |
| TZEIOR 378 | 0 934 | 0 066 | 1 |
| TZEI 2881 | 0 935 | 0 065 | 1 |
| TZEIOR 244 | 0 936 | 0 064 | 1 |
| TZEI 2965 | 0 937 | 0 063 | 1 |
| TZEIOR 476 | 0 937 | 0 063 | 1 |
| TZEI 2984 | 0 938 | 0 062 | 1 |
| TZEIOR 391 | 0 938 | 0 062 | 1 |
| TZEI 2949 | 0 939 | 0 061 | 1 |
| TZEIOR 399 | 0 939 | 0 061 | 1 |
| TZEI 2911 | 0 94 | 0 06 | 1 |
| TZEIOR 230 | 0 94 | 0 06 | 1 |
| TZEI 2941 | 0 94 | 0 06 | 1 |
| TZEIOR 280 | 0 941 | 0 059 | 1 |
| TZEIOR 407 | 0 941 | 0 059 | 1 |
| TZEI 2907 | 0 942 | 0 058 | 1 |
| TZEI 2946 | 0 942 | 0 058 | 1 |
| TZEI 2929 | 0 943 | 0 057 | 1 |
| TZEI 2994 | 0 945 | 0 055 | 1 |
| TZEI 2876 | 0 946 | 0 054 | 1 |
| TZEI 2900 | 0 946 | 0 054 | 1 |
| TZEIOR 387 | 0 948 | 0 052 | 1 |
| TZEIOR 373 | 0 949 | 0 051 | 1 |
| TZEI 2885 | 0 95 | 0 05 | 1 |
| TZEIOR 398 | 0 952 | 0 048 | 1 |
| TZEI 2959 | 0 953 | 0 047 | 1 |
| TZEI 3004 | 0 953 | 0 047 | 1 |
| TZEIOR 210 | 0 954 | 0 046 | 1 |
| TZEIOR 257 | 0 954 | 0 046 | 1 |
| TZEI 2981 | 0 956 | 0 044 | 1 |
| TZEIOR 268 | 0 957 | 0 043 | 1 |
| TZEI 2910 | 0 958 | 0 042 | 1 |
| TZEIOR 260 | 0 96 | 0 04 | 1 |
| TZEI 2937 | 0 962 | 0 038 | 1 |
| TZEI 2940 | 0 962 | 0 038 | 1 |
| TZEI 2916 | 0 966 | 0 034 | 1 |
| TZEI 2969 | 0 967 | 0 033 | 1 |
| TZEI 2953 | 0 969 | 0 031 | 1 |
| TZEI 2987 | 0 969 | 0 031 | 1 |
| TZEI 2977 | 0 97 | 0 03 | 1 |
| TZEI 2982 | 0 97 | 0 03 | 1 |
| TZEIOR 477 | 0 972 | 0 028 | 1 |
| TZEIOR 253 | 0 973 | 0 027 | 1 |
| TZEIOR 246 | 0 974 | 0 026 | 1 |
| TZEIOR 263 | 0 974 | 0 026 | 1 |
| TZEIOR 417 | 0 977 | 0 023 | 1 |
| TZEI 2958 | 0 978 | 0 022 | 1 |
| TZEI 2878 | 0 979 | 0 021 | 1 |
| TZEIOR 471 | 0 979 | 0 021 | 1 |
| TZEI 2925 | 0 98 | 0 02 | 1 |
| TZEI 2888 | 0 981 | 0 019 | 1 |
| TZEIOR 270 | 0 983 | 0 017 | 1 |
| TZEI 2902 | 0 984 | 0 016 | 1 |
| TZEIOR 411 | 0 984 | 0 016 | 1 |
| TZEIOR 245 | 0 985 | 0 015 | 1 |
| TZEI 2926 | 0 988 | 0 012 | 1 |
| TZEIOR 218 | 0 988 | 0 012 | 1 |
| TZEIOR 229 | 0 988 | 0 012 | 1 |
| TZEIOR 206 | 0 989 | 0 011 | 1 |
| TZEI 2948 | 0 989 | 0 011 | 1 |
| TZEIOR 271 | 0 99 | 0 01 | 1 |
| TZEI 2960 | 0 99 | 0 01 | 1 |
| TZEI 2983 | 0 99 | 0 01 | 1 |
| TZEI 2877 | 0 991 | 0 009 | 1 |
| TZEI 2912 | 0 991 | 0 009 | 1 |
| TZEI 2970 | 0 991 | 0 009 | 1 |
| TZEIOR 408 | 0 991 | 0 009 | 1 |
| TZEI 2889 | 0 992 | 0 008 | 1 |
| TZEI 2901 | 0 992 | 0 008 | 1 |
| TZEIOR 258 | 0 992 | 0 008 | 1 |
| TZEI 2936 | 0 992 | 0 008 | 1 |
| TZEI 2971 | 0 992 | 0 008 | 1 |
| TZEIOR 247 | 0 993 | 0 007 | 1 |
| TZEI 2890 | 0 994 | 0 006 | 1 |
| TZEI 2927 | 0 994 | 0 006 | 1 |
| TZEI 2931 | 0 994 | 0 006 | 1 |
| TZEIOR 419 | 0 994 | 0 006 | 1 |
| TZEI 2897 | 0 995 | 0 005 | 1 |
| TZEI 2914 | 0 996 | 0 004 | 1 |
| TZEIOR 474 | 0 996 | 0 004 | 1 |
| TZEI 2913 | 0 997 | 0 003 | 1 |
| TZEIOR 259 | 0 997 | 0 003 | 1 |
| TZEI 2998 | 0 997 | 0 003 | 1 |
| TZEIOR 419 | 0 997 | 0 003 | 1 |
| TZEI 2996 | 0 998 | 0 002 | 1 |
| TZEIOR 382 | 0 998 | 0 002 | 1 |
| TZEIOR 396 | 0 998 | 0 002 | 1 |
| TZEIOR 407 | 0 998 | 0 002 | 1 |
| TZEIOR 483 | 0 998 | 0 002 | 1 |
| TZEI 2997 | 0 999 | 0 001 | 1 |
| TZEIOR 368 | 0 999 | 0 001 | 1 |
| TZEIOR 369 | 0 999 | 0 001 | 1 |
| TZEIOR 384 | 0 999 | 0 001 | 1 |
| TZEIOR 395 | 0 999 | 0 001 | 1 |
| TZEIOR 418 | 0 999 | 0 001 | 1 |
| TZEIOR 472 | 0 999 | 0 001 | 1 |
| TZEIOR 473 | 0 999 | 0 001 | 1 |
| TZEIOR 481 | 0 999 | 0 001 | 1 |
| TZEIOR 482 | 0 999 | 0 001 | 1 |
| TZEIOR 390 | 0 1 | 0 999 | 2 |
| TZEIOR 479 | 0 1 | 0 999 | 2 |
| TZEIOR 507 | 0 1 | 0 999 | 2 |
| TZEIOR 507 | 0 1 | 0 999 | 2 |
| TZEIOR 520 | 0 1 | 0 999 | 2 |
| TZEI 2990 | 0 001 | 0 999 | 2 |
| TZEI 2991 | 0 001 | 0 999 | 2 |
| TZEI 3002 | 0 001 | 0 999 | 2 |
| TZEI 3003 | 0 001 | 0 999 | 2 |
| TZEIOR 374 | 0 001 | 0 999 | 2 |
| TZEIOR 376 | 0 001 | 0 999 | 2 |
| TZEIOR 377 | 0 001 | 0 999 | 2 |
| TZEIOR 388 | 0 001 | 0 999 | 2 |
| TZEIOR 389 | 0 001 | 0 999 | 2 |
| TZEIOR 400 | 0 001 | 0 999 | 2 |
| TZEIOR 401 | 0 001 | 0 999 | 2 |
| TZEIOR 412 | 0 001 | 0 999 | 2 |
| TZEIOR 413 | 0 001 | 0 999 | 2 |
| TZEIOR 414 | 0 001 | 0 999 | 2 |
| TZEIOR 423 | 0 001 | 0 999 | 2 |
| TZEIOR 468 | 0 001 | 0 999 | 2 |
| TZEIOR 478 | 0 001 | 0 999 | 2 |
| TZEIOR 489 | 0 001 | 0 999 | 2 |
| TZEIOR 491 | 0 001 | 0 999 | 2 |
| TZEIOR 492 | 0 001 | 0 999 | 2 |
| TZEIOR 493 | 0 001 | 0 999 | 2 |
| TZEIOR 494 | 0 001 | 0 999 | 2 |
| TZEIOR 496 | 0 001 | 0 999 | 2 |
| TZEIOR 497 | 0 001 | 0 999 | 2 |
| TZEIOR 499 | 0 001 | 0 999 | 2 |
| TZEIOR 501 | 0 001 | 0 999 | 2 |
| TZEIOR 503 | 0 001 | 0 999 | 2 |
| TZEIOR 506 | 0 001 | 0 999 | 2 |
| TZEIOR 510 | 0 001 | 0 999 | 2 |
| TZEIOR 515 | 0 001 | 0 999 | 2 |
| TZEIOR 516 | 0 001 | 0 999 | 2 |
| TZEIOR 517 | 0 001 | 0 999 | 2 |
| TZEIOR 518 | 0 001 | 0 999 | 2 |
| TZEIOR 519 | 0 001 | 0 999 | 2 |
| TZEIOR 521 | 0 001 | 0 999 | 2 |
| TZEIOR 522 | 0 001 | 0 999 | 2 |
| TZEIOR 526 | 0 001 | 0 999 | 2 |
| TZEIOR 527 | 0 001 | 0 999 | 2 |
| TZEIOR 528 | 0 001 | 0 999 | 2 |
| TZEIOR 529 | 0 001 | 0 999 | 2 |
| TZEIOR 645 | 0 001 | 0 999 | 2 |
| TZEIOR 646 | 0 001 | 0 999 | 2 |
| TZEIOR 647 | 0 001 | 0 999 | 2 |
| TZEIOR 648 | 0 001 | 0 999 | 2 |
| TZEIOR 649 | 0 001 | 0 999 | 2 |
| TZEIOR 650 | 0 001 | 0 999 | 2 |
| TZEIOR 656 | 0 001 | 0 999 | 2 |
| TZEIOR 660 | 0 001 | 0 999 | 2 |
| TZEIOR 661 | 0 001 | 0 999 | 2 |
| TZEIOR 662 | 0 001 | 0 999 | 2 |
| TZEIOR 663 | 0 001 | 0 999 | 2 |
| TZEIOR 664 | 0 001 | 0 999 | 2 |
| TZEIOR 666 | 0 001 | 0 999 | 2 |
| TZEIOR 668 | 0 001 | 0 999 | 2 |
| TZEIOR 671 | 0 001 | 0 999 | 2 |
| TZEIOR 672 | 0 001 | 0 999 | 2 |
| TZEIOR 673 | 0 001 | 0 999 | 2 |
| TZEIOR 674 | 0 001 | 0 999 | 2 |
| TZEIOR 675 | 0 001 | 0 999 | 2 |
| TZEIOR 676 | 0 001 | 0 999 | 2 |
| TZEIOR 677 | 0 001 | 0 999 | 2 |
| TZEIOR 679 | 0 001 | 0 999 | 2 |
| TZEIOR 682 | 0 001 | 0 999 | 2 |
| TZEIOR 683 | 0 001 | 0 999 | 2 |
| TZEIOR 684 | 0 001 | 0 999 | 2 |
| TZEIOR 685 | 0 001 | 0 999 | 2 |
| TZEIOR 686 | 0 001 | 0 999 | 2 |
| TZEIOR 688 | 0 001 | 0 999 | 2 |
| TZEIOR 402 | 0 002 | 0 998 | 2 |
| TZEIOR 485 | 0 002 | 0 998 | 2 |
| TZEIOR 488 | 0 002 | 0 998 | 2 |
| TZEIOR 490 | 0 002 | 0 998 | 2 |
| TZEIOR 511 | 0 002 | 0 998 | 2 |
| TZEIOR 530 | 0 002 | 0 998 | 2 |
| TZEIOR 641 | 0 002 | 0 998 | 2 |
| TZEIOR 644 | 0 002 | 0 998 | 2 |
| TZEIOR 487 | 0 004 | 0 996 | 2 |
| TZEIOR 498 | 0 004 | 0 996 | 2 |
| TZEIOR 508 | 0 004 | 0 996 | 2 |
| TZEIOR 640 | 0 005 | 0 995 | 2 |
| TZEIOR 667 | 0 011 | 0 989 | 2 |
| TZEIOR 689 | 0 012 | 0 988 | 2 |
| TZEIOR 678 | 0 015 | 0 985 | 2 |
| TZEIOR 658 | 0 016 | 0 984 | 2 |
| TZEIOR 512 | 0 019 | 0 981 | 2 |
| TZEIOR 659 | 0 019 | 0 981 | 2 |
| TZEIOR 486 | 0 026 | 0 974 | 2 |
| TZEIOR 681 | 0 028 | 0 972 | 2 |
| TZEIOR 653 | 0 032 | 0 968 | 2 |
| TZEIOR 500 | 0 043 | 0 957 | 2 |
| TZEIOR 524 | 0 048 | 0 952 | 2 |
| TZEIOR 531 | 0 05 | 0 95 | 2 |
| TZEIOR 509 | 0 055 | 0 945 | 2 |
| TZEIOR 525 | 0 057 | 0 943 | 2 |
| TZEIOR 687 | 0 067 | 0 933 | 2 |
| TZEIOR 651 | 0 069 | 0 931 | 2 |
| TZEIOR 523 | 0 071 | 0 929 | 2 |
| TZEIOR 520 | 0 075 | 0 925 | 2 |
| TZEIOR 643 | 0 075 | 0 925 | 2 |
| TZEIOR 514 | 0 08 | 0 92 | 2 |
| TZEIOR 665 | 0 083 | 0 917 | 2 |
| TZEIOR 495 | 0 125 | 0 875 | 2 |
| TZEIOR 669 | 0 131 | 0 869 | 2 |
| TZEIOR 680 | 0 141 | 0 859 | 2 |
| TZEIOR 642 | 0 162 | 0 838 | 2 |
| TZEIOR 657 | 0 163 | 0 837 | 2 |
| TZEIOR 513 | 0 164 | 0 836 | 2 |
| TZEIOR 532 | 0 317 | 0 683 | admix |
| TZEIOR 228 | 0 678 | 0 322 | admix |
| TZEI 2879 | 0 71 | 0 29 | admix |
| TZEIOR 670 | 0 712 | 0 288 | admix |
| TZEIOR 385 | 0 714 | 0 286 | admix |
| TZEI 2921 | 0 719 | 0 281 | admix |
| TZEIOR 233 | 0 726 | 0 274 | admix |
| TZEIOR 225 | 0 729 | 0 271 | admix |
| TZEIOR 226 | 0 73 | 0 27 | admix |
| TZEI 2934 | 0 737 | 0 263 | admix |
| TZEIOR 211 | 0 751 | 0 249 | admix |
| TZEI 3005 | 0 756 | 0 244 | admix |
| TZEIOR 264 | 0 763 | 0 237 | admix |
| TZEIOR 214 | 0 767 | 0 233 | admix |
| TZEI 2957 | 0 776 | 0 224 | admix |
| TZEI 2947 | 0 782 | 0 218 | admix |
| TZEIOR 243 | 0 787 | 0 213 | admix |
| TZEI 2943 | 0 789 | 0 211 | admix |
| TZEI 2962 | 0 791 | 0 209 | admix |
