# Supplementary material for: Genetic diversity and population structure of early-maturing yellow and orange maize inbred lines for improved breeding applications
Source: PLoS One. 2026 Sep 25;21(9):e0358751. doi: 10.1371/journal.pone.0358751 (PMC13614566; doi:10.1371/journal.pone.0358751)
Supplement: Supplementary file 4 [file pone.0358751.s003.docx]

Supplementary Table S3: Population stratification based on the discriminant analysis of principal components approach revealed two genetic groups

| Inbred | K1 | K2 | Cluster |
| --- | --- | --- | --- |
| TZEI 2876 | 1 | 5 23E-120 | 1 |
| TZEI 2877 | 1 | 2 08E-118 | 1 |
| TZEI 2878 | 1 | 3 12E-121 | 1 |
| TZEI 2879 | 1 | 2 38E-118 | 1 |
| TZEI 2880 | 1 | 2 58E-126 | 1 |
| TZEI 2881 | 1 | 4 19E-134 | 1 |
| TZEI 2882 | 1 | 1 87E-142 | 1 |
| TZEI 2883 | 1 | 5 68E-117 | 1 |
| TZEI 2884 | 1 | 2 04E-125 | 1 |
| TZEI 2885 | 1 | 1 52E-133 | 1 |
| TZEI 2886 | 1 | 1 07E-127 | 1 |
| TZEI 2887 | 1 | 5 71E-128 | 1 |
| TZEI 2888 | 1 | 1 44E-125 | 1 |
| TZEI 2889 | 1 | 2 41E-121 | 1 |
| TZEI 2890 | 1 | 6 40E-135 | 1 |
| TZEI 2891 | 1 | 3 41E-124 | 1 |
| TZEI 2892 | 1 | 3 86E-121 | 1 |
| TZEI 2893 | 1 | 2 45E-129 | 1 |
| TZEI 2894 | 1 | 5 22E-137 | 1 |
| TZEI 2895 | 1 | 4 24E-117 | 1 |
| TZEI 2896 | 1 | 3 58E-129 | 1 |
| TZEI 2897 | 1 | 1 09E-144 | 1 |
| TZEI 2898 | 1 | 3 47E-138 | 1 |
| TZEI 2899 | 1 | 1 33E-128 | 1 |
| TZEI 2900 | 1 | 3 29E-106 | 1 |
| TZEI 2901 | 1 | 3 43E-131 | 1 |
| TZEI 2902 | 1 | 2 74E-132 | 1 |
| TZEI 2903 | 1 | 7 23E-130 | 1 |
| TZEI 2904 | 1 | 7 10E-132 | 1 |
| TZEI 2905 | 1 | 2 02E-118 | 1 |
| TZEI 2906 | 1 | 3 56E-135 | 1 |
| TZEI 2907 | 1 | 1 74E-153 | 1 |
| TZEI 2908 | 1 | 1 65E-120 | 1 |
| TZEI 2909 | 1 | 8 22E-127 | 1 |
| TZEI 2910 | 1 | 4 01E-126 | 1 |
| TZEI 2911 | 1 | 6 64E-139 | 1 |
| TZEI 2912 | 1 | 6 27E-116 | 1 |
| TZEI 2913 | 1 | 2 15E-130 | 1 |
| TZEI 2914 | 1 | 1 66E-134 | 1 |
| TZEI 2915 | 1 | 2 86E-119 | 1 |
| TZEI 2916 | 1 | 1 23E-127 | 1 |
| TZEI 2917 | 1 | 1 00E-117 | 1 |
| TZEI 2918 | 1 | 4 91E-122 | 1 |
| TZEI 2919 | 1 | 3 64E-143 | 1 |
| TZEI 2920 | 1 | 3 77E-124 | 1 |
| TZEI 2921 | 1 | 5 52E-119 | 1 |
| TZEI 2923 | 1 | 5 98E-115 | 1 |
| TZEI 2924 | 1 | 3 85E-118 | 1 |
| TZEI 2925 | 1 | 1 18E-108 | 1 |
| TZEI 2926 | 1 | 5 64E-122 | 1 |
| TZEI 2927 | 1 | 1 17E-147 | 1 |
| TZEI 2928 | 1 | 1 61E-128 | 1 |
| TZEI 2929 | 1 | 4 25E-125 | 1 |
| TZEI 2930 | 1 | 7 27E-101 | 1 |
| TZEI 2931 | 1 | 3 67E-149 | 1 |
| TZEI 2932 | 1 | 1 53E-125 | 1 |
| TZEI 2933 | 1 | 7 47E-116 | 1 |
| TZEI 2934 | 1 | 1 22E-124 | 1 |
| TZEI 2935 | 1 | 5 61E-127 | 1 |
| TZEI 2936 | 1 | 2 60E-125 | 1 |
| TZEI 2937 | 1 | 6 31E-117 | 1 |
| TZEI 2938 | 1 | 1 59E-128 | 1 |
| TZEI 2939 | 1 | 5 07E-123 | 1 |
| TZEI 2940 | 1 | 9 92E-125 | 1 |
| TZEI 2941 | 1 | 1 54E-120 | 1 |
| TZEI 2942 | 1 | 2 03E-132 | 1 |
| TZEI 2943 | 1 | 7 54E-100 | 1 |
| TZEI 2944 | 1 | 1 77E-121 | 1 |
| TZEI 2945 | 1 | 1 34E-126 | 1 |
| TZEI 2946 | 1 | 3 93E-122 | 1 |
| TZEI 2947 | 1 | 6 82E-123 | 1 |
| TZEI 2948 | 1 | 8 83E-124 | 1 |
| TZEI 2949 | 1 | 4 85E-116 | 1 |
| TZEI 2950 | 1 | 4 64E-123 | 1 |
| TZEI 2951 | 1 | 5 48E-141 | 1 |
| TZEI 2952 | 1 | 2 32E-128 | 1 |
| TZEI 2953 | 1 | 1 37E-133 | 1 |
| TZEI 2954 | 1 | 1 46E-122 | 1 |
| TZEI 2955 | 1 | 1 77E-132 | 1 |
| TZEI 2956 | 1 | 3 48E-130 | 1 |
| TZEI 2957 | 1 | 1 79E-102 | 1 |
| TZEI 2958 | 1 | 3 72E-128 | 1 |
| TZEI 2959 | 1 | 3 49E-120 | 1 |
| TZEI 2960 | 1 | 3 56E-122 | 1 |
| TZEI 2961 | 1 | 1 38E-131 | 1 |
| TZEI 2962 | 1 | 1 80E-102 | 1 |
| TZEI 2963 | 1 | 1 08E-114 | 1 |
| TZEI 2964 | 1 | 2 00E-136 | 1 |
| TZEI 2965 | 1 | 8 73E-128 | 1 |
| TZEI 2966 | 1 | 5 49E-121 | 1 |
| TZEI 2967 | 1 | 8 22E-135 | 1 |
| TZEI 2968 | 1 | 3 68E-131 | 1 |
| TZEI 2969 | 1 | 4 36E-128 | 1 |
| TZEI 2970 | 1 | 7 37E-129 | 1 |
| TZEI 2971 | 1 | 1 70E-124 | 1 |
| TZEI 2972 | 1 | 4 85E-125 | 1 |
| TZEI 2973 | 1 | 1 68E-128 | 1 |
| TZEI 2974 | 1 | 4 43E-130 | 1 |
| TZEI 2975 | 1 | 1 43E-139 | 1 |
| TZEI 2977 | 1 | 9 03E-129 | 1 |
| TZEI 2978 | 1 | 5 13E-128 | 1 |
| TZEI 2979 | 1 | 8 21E-128 | 1 |
| TZEI 2980 | 1 | 4 71E-136 | 1 |
| TZEI 2981 | 1 | 7 54E-126 | 1 |
| TZEI 2982 | 1 | 3 60E-123 | 1 |
| TZEI 2983 | 1 | 9 26E-131 | 1 |
| TZEI 2984 | 1 | 5 74E-134 | 1 |
| TZEI 2984 | 1 | 1 08E-135 | 1 |
| TZEI 2985 | 1 | 1 28E-132 | 1 |
| TZEI 2986 | 1 | 2 35E-138 | 1 |
| TZEI 2987 | 1 | 9 52E-133 | 1 |
| TZEI 2988 | 1 | 7 69E-119 | 1 |
| TZEI 2989 | 1 | 2 26E-116 | 1 |
| TZEI 2992 | 1 | 1 22E-129 | 1 |
| TZEI 2993 | 1 | 2 61E-133 | 1 |
| TZEI 2994 | 1 | 1 12E-133 | 1 |
| TZEI 2995 | 1 | 6 17E-118 | 1 |
| TZEI 2996 | 1 | 7 74E-119 | 1 |
| TZEI 2997 | 1 | 5 90E-123 | 1 |
| TZEI 2998 | 1 | 4 23E-127 | 1 |
| TZEI 2999 | 1 | 5 90E-130 | 1 |
| TZEI 3000 | 1 | 1 74E-115 | 1 |
| TZEI 3001 | 1 | 2 21E-103 | 1 |
| TZEI 3004 | 1 | 1 57E-131 | 1 |
| TZEI 3005 | 1 | 3 50E-99 | 1 |
| TZEIOR 204 | 1 | 1 48E-115 | 1 |
| TZEIOR 205 | 1 | 1 15E-125 | 1 |
| TZEIOR 206 | 1 | 3 29E-127 | 1 |
| TZEIOR 207 | 1 | 1 03E-134 | 1 |
| TZEIOR 208 | 1 | 1 20E-128 | 1 |
| TZEIOR 209 | 1 | 1 24E-119 | 1 |
| TZEIOR 210 | 1 | 4 68E-143 | 1 |
| TZEIOR 211 | 1 | 3 56E-104 | 1 |
| TZEIOR 212 | 1 | 1 90E-116 | 1 |
| TZEIOR 213 | 1 | 3 01E-128 | 1 |
| TZEIOR 214 | 1 | 5 35E-133 | 1 |
| TZEIOR 215 | 1 | 1 96E-129 | 1 |
| TZEIOR 216 | 1 | 2 74E-113 | 1 |
| TZEIOR 217 | 1 | 4 74E-133 | 1 |
| TZEIOR 218 | 1 | 2 73E-122 | 1 |
| TZEIOR 219 | 1 | 8 22E-136 | 1 |
| TZEIOR 220 | 1 | 2 33E-121 | 1 |
| TZEIOR 221 | 1 | 1 25E-114 | 1 |
| TZEIOR 222 | 1 | 6 72E-117 | 1 |
| TZEIOR 223 | 1 | 1 85E-134 | 1 |
| TZEIOR 224 | 1 | 6 23E-137 | 1 |
| TZEIOR 225 | 1 | 1 72E-127 | 1 |
| TZEIOR 226 | 1 | 1 94E-125 | 1 |
| TZEIOR 227 | 1 | 3 20E-117 | 1 |
| TZEIOR 228 | 1 | 6 57E-84 | 1 |
| TZEIOR 229 | 1 | 6 10E-123 | 1 |
| TZEIOR 230 | 1 | 8 97E-142 | 1 |
| TZEIOR 231 | 1 | 1 39E-128 | 1 |
| TZEIOR 232 | 1 | 5 07E-119 | 1 |
| TZEIOR 233 | 1 | 8 17E-119 | 1 |
| TZEIOR 234 | 1 | 8 40E-137 | 1 |
| TZEIOR 235 | 1 | 2 31E-146 | 1 |
| TZEIOR 236 | 1 | 2 09E-128 | 1 |
| TZEIOR 237 | 1 | 1 66E-128 | 1 |
| TZEIOR 238 | 1 | 1 39E-122 | 1 |
| TZEIOR 240 | 1 | 1 01E-128 | 1 |
| TZEIOR 241 | 1 | 8 48E-132 | 1 |
| TZEIOR 242 | 1 | 6 78E-122 | 1 |
| TZEIOR 243 | 1 | 2 64E-126 | 1 |
| TZEIOR 244 | 1 | 3 93E-127 | 1 |
| TZEIOR 245 | 1 | 2 42E-132 | 1 |
| TZEIOR 246 | 1 | 7 76E-126 | 1 |
| TZEIOR 247 | 1 | 3 72E-134 | 1 |
| TZEIOR 248 | 1 | 6 83E-118 | 1 |
| TZEIOR 249 | 1 | 8 04E-131 | 1 |
| TZEIOR 250 | 1 | 6 48E-129 | 1 |
| TZEIOR 251 | 1 | 3 71E-121 | 1 |
| TZEIOR 252 | 1 | 3 67E-133 | 1 |
| TZEIOR 253 | 1 | 8 59E-152 | 1 |
| TZEIOR 254 | 1 | 1 12E-131 | 1 |
| TZEIOR 255 | 1 | 9 58E-128 | 1 |
| TZEIOR 256 | 1 | 4 35E-127 | 1 |
| TZEIOR 257 | 1 | 1 14E-124 | 1 |
| TZEIOR 258 | 1 | 1 85E-127 | 1 |
| TZEIOR 259 | 1 | 5 25E-132 | 1 |
| TZEIOR 260 | 1 | 5 18E-128 | 1 |
| TZEIOR 261 | 1 | 8 69E-124 | 1 |
| TZEIOR 262 | 1 | 1 72E-133 | 1 |
| TZEIOR 263 | 1 | 1 06E-132 | 1 |
| TZEIOR 264 | 1 | 1 01E-112 | 1 |
| TZEIOR 265 | 1 | 4 56E-126 | 1 |
| TZEIOR 266 | 1 | 4 44E-131 | 1 |
| TZEIOR 267 | 1 | 7 62E-133 | 1 |
| TZEIOR 268 | 1 | 2 62E-120 | 1 |
| TZEIOR 269 | 1 | 2 30E-128 | 1 |
| TZEIOR 270 | 1 | 5 01E-123 | 1 |
| TZEIOR 271 | 1 | 5 77E-128 | 1 |
| TZEIOR 272 | 1 | 2 70E-137 | 1 |
| TZEIOR 273 | 1 | 1 53E-128 | 1 |
| TZEIOR 274 | 1 | 9 00E-136 | 1 |
| TZEIOR 275 | 1 | 1 14E-121 | 1 |
| TZEIOR 276 | 1 | 1 06E-126 | 1 |
| TZEIOR 277 | 1 | 2 57E-125 | 1 |
| TZEIOR 278 | 1 | 3 20E-120 | 1 |
| TZEIOR 279 | 1 | 4 56E-112 | 1 |
| TZEIOR 280 | 1 | 2 66E-125 | 1 |
| TZEIOR 281 | 1 | 7 99E-127 | 1 |
| TZEIOR 364 | 1 | 7 50E-102 | 1 |
| TZEIOR 367 | 1 | 1 42E-135 | 1 |
| TZEIOR 368 | 1 | 5 78E-136 | 1 |
| TZEIOR 369 | 1 | 2 07E-125 | 1 |
| TZEIOR 370 | 1 | 1 26E-110 | 1 |
| TZEIOR 371 | 1 | 1 47E-120 | 1 |
| TZEIOR 373 | 1 | 9 52E-130 | 1 |
| TZEIOR 378 | 1 | 1 34E-124 | 1 |
| TZEIOR 379 | 1 | 4 28E-116 | 1 |
| TZEIOR 380 | 1 | 1 61E-116 | 1 |
| TZEIOR 381 | 1 | 1 74E-126 | 1 |
| TZEIOR 382 | 1 | 2 03E-134 | 1 |
| TZEIOR 384 | 1 | 9 12E-121 | 1 |
| TZEIOR 385 | 1 | 4 68E-111 | 1 |
| TZEIOR 386 | 1 | 1 60E-118 | 1 |
| TZEIOR 387 | 1 | 1 30E-130 | 1 |
| TZEIOR 390 | 1 | 2 48E-132 | 1 |
| TZEIOR 391 | 1 | 3 25E-119 | 1 |
| TZEIOR 392 | 1 | 3 26E-123 | 1 |
| TZEIOR 393 | 1 | 6 87E-129 | 1 |
| TZEIOR 395 | 1 | 1 17E-119 | 1 |
| TZEIOR 396 | 1 | 4 19E-120 | 1 |
| TZEIOR 397 | 1 | 1 81E-121 | 1 |
| TZEIOR 398 | 1 | 1 22E-131 | 1 |
| TZEIOR 399 | 1 | 4 20E-135 | 1 |
| TZEIOR 403 | 1 | 7 22E-124 | 1 |
| TZEIOR 405 | 1 | 1 87E-120 | 1 |
| TZEIOR 406 | 1 | 7 39E-128 | 1 |
| TZEIOR 407 | 1 | 3 51E-122 | 1 |
| TZEIOR 407 | 1 | 3 06E-127 | 1 |
| TZEIOR 408 | 1 | 3 23E-114 | 1 |
| TZEIOR 409 | 1 | 6 23E-116 | 1 |
| TZEIOR 410 | 1 | 2 76E-135 | 1 |
| TZEIOR 411 | 1 | 1 31E-136 | 1 |
| TZEIOR 415 | 1 | 1 19E-125 | 1 |
| TZEIOR 416 | 1 | 2 76E-132 | 1 |
| TZEIOR 417 | 1 | 7 08E-121 | 1 |
| TZEIOR 418 | 1 | 5 01E-135 | 1 |
| TZEIOR 419 | 1 | 1 49E-125 | 1 |
| TZEIOR 419 | 1 | 2 18E-127 | 1 |
| TZEIOR 420 | 1 | 8 77E-120 | 1 |
| TZEIOR 421 | 1 | 2 58E-136 | 1 |
| TZEIOR 422 | 1 | 1 06E-122 | 1 |
| TZEIOR 469 | 1 | 1 71E-128 | 1 |
| TZEIOR 470 | 1 | 8 78E-129 | 1 |
| TZEIOR 471 | 1 | 3 72E-123 | 1 |
| TZEIOR 472 | 1 | 1 61E-129 | 1 |
| TZEIOR 473 | 1 | 7 13E-127 | 1 |
| TZEIOR 474 | 1 | 1 33E-125 | 1 |
| TZEIOR 475 | 1 | 1 29E-124 | 1 |
| TZEIOR 476 | 1 | 5 78E-122 | 1 |
| TZEIOR 477 | 1 | 1 05E-131 | 1 |
| TZEIOR 479 | 1 | 2 83E-115 | 1 |
| TZEIOR 480 | 1 | 1 18E-130 | 1 |
| TZEIOR 480 | 1 | 6 72E-117 | 1 |
| TZEIOR 481 | 1 | 4 12E-120 | 1 |
| TZEIOR 482 | 1 | 1 36E-126 | 1 |
| TZEIOR 483 | 1 | 2 75E-131 | 1 |
| TZEIOR 483 | 1 | 2 14E-127 | 1 |
| TZEIOR 484 | 1 | 1 59E-114 | 1 |
| TZEIOR 670 | 1 | 1 38E-67 | 1 |
| TZEI 2990 | 1 71E-128 | 1 | 2 |
| TZEI 2991 | 1 15E-123 | 1 | 2 |
| TZEI 3002 | 2 06E-124 | 1 | 2 |
| TZEI 3003 | 6 18E-135 | 1 | 2 |
| TZEIOR 374 | 1 70E-123 | 1 | 2 |
| TZEIOR 376 | 9 75E-136 | 1 | 2 |
| TZEIOR 377 | 4 89E-137 | 1 | 2 |
| TZEIOR 388 | 6 63E-127 | 1 | 2 |
| TZEIOR 389 | 3 61E-129 | 1 | 2 |
| TZEIOR 390 | 2 82E-117 | 1 | 2 |
| TZEIOR 400 | 1 03E-126 | 1 | 2 |
| TZEIOR 401 | 2 24E-132 | 1 | 2 |
| TZEIOR 402 | 8 83E-114 | 1 | 2 |
| TZEIOR 412 | 6 12E-125 | 1 | 2 |
| TZEIOR 413 | 3 97E-121 | 1 | 2 |
| TZEIOR 414 | 6 85E-120 | 1 | 2 |
| TZEIOR 423 | 5 07E-131 | 1 | 2 |
| TZEIOR 468 | 9 79E-138 | 1 | 2 |
| TZEIOR 478 | 4 20E-132 | 1 | 2 |
| TZEIOR 479 | 2 59E-124 | 1 | 2 |
| TZEIOR 485 | 1 63E-112 | 1 | 2 |
| TZEIOR 486 | 3 66E-135 | 1 | 2 |
| TZEIOR 487 | 7 38E-135 | 1 | 2 |
| TZEIOR 488 | 1 65E-128 | 1 | 2 |
| TZEIOR 489 | 2 87E-133 | 1 | 2 |
| TZEIOR 490 | 1 08E-117 | 1 | 2 |
| TZEIOR 491 | 8 06E-119 | 1 | 2 |
| TZEIOR 492 | 6 00E-130 | 1 | 2 |
| TZEIOR 493 | 7 50E-132 | 1 | 2 |
| TZEIOR 494 | 3 09E-136 | 1 | 2 |
| TZEIOR 495 | 2 03E-110 | 1 | 2 |
| TZEIOR 496 | 4 33E-134 | 1 | 2 |
| TZEIOR 497 | 9 49E-128 | 1 | 2 |
| TZEIOR 498 | 1 74E-139 | 1 | 2 |
| TZEIOR 499 | 9 70E-125 | 1 | 2 |
| TZEIOR 500 | 4 46E-114 | 1 | 2 |
| TZEIOR 501 | 5 70E-127 | 1 | 2 |
| TZEIOR 503 | 2 37E-116 | 1 | 2 |
| TZEIOR 506 | 2 79E-126 | 1 | 2 |
| TZEIOR 507 | 3 73E-119 | 1 | 2 |
| TZEIOR 50B | 5 14E-117 | 1 | 2 |
| TZEIOR 508 | 7 74E-130 | 1 | 2 |
| TZEIOR 509 | 4 47E-119 | 1 | 2 |
| TZEIOR 510 | 6 56E-130 | 1 | 2 |
| TZEIOR 511 | 8 28E-122 | 1 | 2 |
| TZEIOR 512 | 2 15E-132 | 1 | 2 |
| TZEIOR 513 | 1 76E-103 | 1 | 2 |
| TZEIOR 514 | 2 50E-105 | 1 | 2 |
| TZEIOR 515 | 2 12E-129 | 1 | 2 |
| TZEIOR 516 | 6 46E-125 | 1 | 2 |
| TZEIOR 517 | 1 23E-129 | 1 | 2 |
| TZEIOR 518 | 7 65E-118 | 1 | 2 |
| TZEIOR 519 | 1 70E-122 | 1 | 2 |
| TZEIOR 520 | 7 65E-132 | 1 | 2 |
| TZEIOR 520 | 1 98E-122 | 1 | 2 |
| TZEIOR 521 | 1 36E-131 | 1 | 2 |
| TZEIOR 522 | 2 72E-120 | 1 | 2 |
| TZEIOR 523 | 4 62E-129 | 1 | 2 |
| TZEIOR 524 | 1 22E-127 | 1 | 2 |
| TZEIOR 525 | 7 17E-112 | 1 | 2 |
| TZEIOR 526 | 1 23E-127 | 1 | 2 |
| TZEIOR 527 | 4 79E-132 | 1 | 2 |
| TZEIOR 528 | 1 05E-132 | 1 | 2 |
| TZEIOR 529 | 6 02E-120 | 1 | 2 |
| TZEIOR 530 | 1 44E-109 | 1 | 2 |
| TZEIOR 531 | 2 76E-110 | 1 | 2 |
| TZEIOR 532 | 9 33E-34 | 1 | 2 |
| TZEIOR 640 | 4 15E-140 | 1 | 2 |
| TZEIOR 641 | 7 74E-107 | 1 | 2 |
| TZEIOR 642 | 4 06E-115 | 1 | 2 |
| TZEIOR 643 | 2 32E-129 | 1 | 2 |
| TZEIOR 644 | 1 84E-135 | 1 | 2 |
| TZEIOR 645 | 1 40E-125 | 1 | 2 |
| TZEIOR 646 | 9 98E-130 | 1 | 2 |
| TZEIOR 647 | 1 89E-121 | 1 | 2 |
| TZEIOR 648 | 1 26E-120 | 1 | 2 |
| TZEIOR 649 | 6 79E-119 | 1 | 2 |
| TZEIOR 650 | 9 60E-135 | 1 | 2 |
| TZEIOR 651 | 4 82E-126 | 1 | 2 |
| TZEIOR 653 | 1 24E-128 | 1 | 2 |
| TZEIOR 656 | 3 36E-115 | 1 | 2 |
| TZEIOR 657 | 3 70E-116 | 1 | 2 |
| TZEIOR 658 | 2 53E-130 | 1 | 2 |
| TZEIOR 659 | 4 75E-128 | 1 | 2 |
| TZEIOR 660 | 6 68E-129 | 1 | 2 |
| TZEIOR 661 | 2 45E-127 | 1 | 2 |
| TZEIOR 662 | 3 50E-132 | 1 | 2 |
| TZEIOR 663 | 1 31E-121 | 1 | 2 |
| TZEIOR 664 | 6 01E-118 | 1 | 2 |
| TZEIOR 665 | 3 81E-103 | 1 | 2 |
| TZEIOR 666 | 2 71E-129 | 1 | 2 |
| TZEIOR 667 | 4 17E-134 | 1 | 2 |
| TZEIOR 668 | 1 44E-139 | 1 | 2 |
| TZEIOR 669 | 4 52E-124 | 1 | 2 |
| TZEIOR 671 | 1 42E-124 | 1 | 2 |
| TZEIOR 672 | 7 10E-129 | 1 | 2 |
| TZEIOR 673 | 3 78E-126 | 1 | 2 |
| TZEIOR 674 | 3 23E-126 | 1 | 2 |
| TZEIOR 675 | 1 37E-125 | 1 | 2 |
| TZEIOR 676 | 2 85E-132 | 1 | 2 |
| TZEIOR 677 | 2 03E-136 | 1 | 2 |
| TZEIOR 678 | 2 67E-133 | 1 | 2 |
| TZEIOR 679 | 6 37E-134 | 1 | 2 |
| TZEIOR 680 | 1 02E-121 | 1 | 2 |
| TZEIOR 681 | 1 10E-126 | 1 | 2 |
| TZEIOR 682 | 5 46E-122 | 1 | 2 |
| TZEIOR 683 | 2 33E-124 | 1 | 2 |
| TZEIOR 684 | 2 67E-130 | 1 | 2 |
| TZEIOR 685 | 5 30E-134 | 1 | 2 |
| TZEIOR 686 | 2 41E-138 | 1 | 2 |
| TZEIOR 687 | 9 60E-120 | 1 | 2 |
| TZEIOR 688 | 9 25E-130 | 1 | 2 |
| TZEIOR 689 | 6 14E-130 | 1 | 2 |
