# Supplementary material for: Genetic diversity and population structure of early-maturing yellow and orange maize inbred lines for improved breeding applications
Source: PLoS One. 2026 Sep 25;21(9):e0358751. doi: 10.1371/journal.pone.0358751 (PMC13614566; doi:10.1371/journal.pone.0358751)
Supplement: Supplementary file 5 [file pone.0358751.s004.docx]

**Response to Reviewers**

Manuscript Title: Genetic Diversity and Population Structure of Early-Maturing Yellow and Orange Maize Inbred Lines for Improved Breeding Applications

Dear Editor and Reviewers,

We sincerely thank the Editor and the Reviewers for their thorough evaluation of our manuscript and for the constructive comments and suggestions. We have carefully revised the manuscript and believe that the changes have substantially improved its scientific rigor, clarity, and reproducibility.

All reviewer comments have been addressed point-by-point below. Reviewer comments are shown in italics, followed by our responses.

**REVIEWER 3**

Comment 1:
"The manuscript lacks sufficient information on grain yield performance and quality characteristics of the germplasm."

Response:
We thank the reviewer for this important observation. The primary objective of the study was genomic characterization of the inbred lines rather than phenotypic evaluation. Nevertheless, we have clarified in the Discussion section that the evaluated inbred lines were previously developed and selected within the IITA Maize Improvement Program under recurrent screening for grain yield performance, stress tolerance, and kernel quality traits. This addition emphasizes the breeding relevance of the germplasm analyzed in the present study.

Revision made: A new paragraph was added in the Breeding Implications and Future Directions section describing the agronomic relevance and breeding history of the evaluated germplasm.

**REVIEWER 4**

Comment 1:
"The manuscript overinterprets molecular clustering as evidence of validated heterotic groups."

Response:
We appreciate this observation and agree that molecular clustering alone cannot conclusively validate heterotic groups. Throughout the manuscript, terminology was revised to avoid overstatement. References to "confirmed heterotic groups" were replaced with "putative heterotic groups," "molecular clusters," or "putative molecular heterotic groups" where appropriate.

Revision made: Changes were implemented throughout the Abstract, Results, Discussion, and Conclusions.

Comment 2:
"Claims regarding hybrid prediction and tester selection are not sufficiently supported."

Response:
We agree and have moderated these statements throughout the manuscript. Previous wording implying direct hybrid prediction or validated tester assignment has been replaced with more cautious language indicating potential utility for future hybrid evaluation and tester development studies.

Revision made: Relevant sections in the Results, Discussion, and Breeding Implications sections were revised accordingly.

Comment 3:
"Biological validation of the identified groups is not demonstrated."

Response:
We thank the reviewer for this important clarification. Statements referring to "biological validation" were removed. These were replaced with wording indicating consistency between pedigree background and molecular clustering patterns rather than direct biological validation.

Revision made: The Relationship Between Source Populations and Putative Heterotic Groups section and associated discussion were revised.

Comment 4:
"The discussion of linkage disequilibrium extends beyond the evidence presented."

Response:
We agree. The LD discussion has been revised to avoid unsupported implications regarding QTL detection efficiency, GWAS performance, and genomic selection effectiveness. The revised discussion now focuses on historical recombination patterns and potential utility for future genetic studies.

Revision made: The Linkage Disequilibrium and Recombination History section was substantially revised.

Comment 5:
"The core set does not adequately demonstrate preservation of diversity."

Response:
We thank the reviewer for this suggestion. Additional analyses were conducted to compare the full collection and the candidate core subsets using allelic richness, expected heterozygosity, observed heterozygosity, Shannon diversity index, mean genetic distance, and PCA-based diversity space coverage. These analyses demonstrate the effectiveness of the proposed core subset in preserving genetic diversity.

Revision made: A new section entitled "Core Subset Selection and Diversity Characterization" was added, together with Table 6 and Figures 8–9.

**REVIEWER 5**

Comment 1:
"The methodology lacks sufficient detail for reproducibility."

Response:
We appreciate this observation and have substantially expanded the Materials and Methods section. Additional details regarding marker quality control, population structure analysis, LD analysis, phylogenetic analysis, and core set selection have been incorporated.

Revision made: Methods section expanded throughout.

Comment 2:
"Clarify the procedures used for population structure analysis."

Response:
Additional details regarding STRUCTURE, DAPC, cluster assignment criteria, Evanno method implementation, and phylogenetic reconstruction were added.

Revision made: Population structure methodology was revised and expanded.

**REVIEWER 6**

Comment 1:
"Provide a clearer description of the genotyping platform."

Response:
We thank the reviewer for identifying this important issue. The genotyping procedure was completely revised to clearly describe the DArTag platform, distinguish it from DArTseq, explain the targeted SNP approach, and specify the maize reference genome (B73 RefGen_v5) used during SNP calling.

Revision made: The Leaf Sample Collection and Genotyping Procedure section was extensively revised.

Comment 2:
"Provide references supporting filtering thresholds and analytical procedures."

Response:
Relevant references supporting marker quality control thresholds, LD analysis, population structure analysis, and core collection development were added throughout the Methods section.

Revision made: Additional citations have been incorporated throughout the Materials and Methods section.

Comment 3:
"Clarify the software and algorithms used for core set identification."

Response:
Additional details regarding CoreHunter 3, the simulated annealing optimization procedure, the stratified CoreHunter approach, and the greedy stepwise heuristic algorithm were added. Evaluation metrics used to compare core sets were also clearly described.

Revision made: The core collection methodology was rewritten and expanded substantially.

Comment 4:
"Provide information regarding marker numbers before and after filtering."

Response:
The manuscript now clearly reports the initial DArTag dataset of 3,305 SNP markers and the final filtered dataset of 1,954 high-quality SNP markers used for downstream analyses.

Revision made: Clarified in both the Methods and Results sections.

**EDITORIAL CHANGES**

In addition to the reviewer-specific revisions, we have:

i. Standardized terminology throughout the manuscript.
ii. Replaced overinterpreted statements with more cautious and evidence-based language.
iii. Corrected typographical and grammatical errors.
iv. Harmonized SNP numbers across all sections.
v. Updated figure captions and table descriptions.
vi. Improved consistency between Results and Discussion sections.
vii. Expanded the discussion of the practical implications of the identified core subset for tropical maize breeding.

We sincerely thank the Editor and Reviewers for their valuable comments and suggestions. We believe the revised manuscript has been substantially improved and hope it is now suitable for publication.

Sincerely,

Idris Ishola Adejumobi (On behalf of all Authors)
