# Supplementary material for: Genetic diversity and population structure of early-maturing yellow and orange maize inbred lines for improved breeding applications
Source: PLoS One. 2026 Sep 25;21(9):e0358751. doi: 10.1371/journal.pone.0358751 (PMC13614566; doi:10.1371/journal.pone.0358751)
Supplement: Supplementary file 6 [file pone.0358751.s006.docx]

**Response to the Editor**

We sincerely thank the Editor for the careful evaluation of our revised manuscript and for the encouraging comments. We greatly appreciate your positive assessment that the revisions have strengthened the manuscript and that the additional analyses performed for the core set validation have improved the study. We have carefully addressed all the remaining minor comments as outlined below.

**Comment 1:**

**Abstract correction:** Revise the statement regarding the core set size from "15%" to "20%" to match the Methods, Results, and Table 6, where a 76-line (20%) core is clearly described.

**Response**
Thank you for identifying this inconsistency. The Abstract has been revised to correctly state that the core collection comprised **76 lines (20%)**, ensuring consistency with the Methods, Results, and Table 6.

**Comment 2:**

**Typographical fixes:**

- Change "admixt" to "admixed" in the population structure methodology.
- Correct punctuation: "IITA. Ibadan" → "IITA, Ibadan."

**Response**
Thank you for pointing out these typographical errors. Both corrections have been made in the revised manuscript. Specifically, "admixt" has been corrected to "admixed," and the punctuation has been revised from "IITA. Ibadan" to "IITA, Ibadan."

**Comment 3:**

**Data Availability Statement:** Please rephrase the statement to state clearly and directly that all SNP data underlying the findings are publicly available at the Figshare repository with the provided DOI, rather than using vague terms like "part of the data" or "extended data."

**Response**
We appreciate this suggestion. The Data Availability Statement has been revised to clearly state that **all SNP data underlying the findings of this study are publicly available in the Figshare repository through the provided DOI**, in accordance with the journal's data availability requirements.

**Comment 4:**

**Verify completeness:** Please confirm that Table 2 and Figure 1 are complete and correctly formatted in your final submission files, as they were not fully visible in the review PDF.

**Response**
Thank you for bringing this to our attention. We have carefully verified the final submission files and confirm that Table 2 and Figure 1 are complete, correctly formatted, and fully visible in the revised manuscript submitted for publication.

Once again, we sincerely thank the Editor for the constructive comments and careful handling of our manuscript. We appreciate the opportunity to revise the manuscript and believe that the changes made have further improved its clarity, consistency, and overall quality. We hope that the revised version is now suitable for publication in PLOS ONE.
