# Supplementary material for: Genetic diversity and population structure of early-maturing yellow and orange maize inbred lines for improved breeding applications
Source: PLoS One. 2026 Sep 25;21(9):e0358751. doi: 10.1371/journal.pone.0358751 (PMC13614566; doi:10.1371/journal.pone.0358751)
Supplement: Supplementary file 7 [file pone.0358751.s007.docx]

**Response to the Editor**

We sincerely thank the Editor for the careful review of our manuscript and for the constructive comments. We have carefully considered all the points raised and have made the corresponding revisions to improve the clarity, consistency, and methodological presentation of the manuscript. Our point-by-point responses are provided below.

**Comment 1**

*Please check space between [5,8] and [19,20].*

**Response**
Thank you for pointing this out. We carefully checked the in-text citations throughout the manuscript and corrected the spacing and citation formatting for **[5,8]** and **[19,20]** to ensure consistency with the journal's citation style.

**Comment 2**

*Table 1, legend – "Geographic origin", please check and revise.*

**Response**
Thank you for this observation. We reviewed the caption which included "Geographic origin" in Table 1 and removed this description because the relevant information was already adequately captured in-text where the germplasm is described as “tropical germplasm”. We therefore considered the information in the table caption redundant and removed it to improve the clarity and conciseness of Table 1.

**Comment 3**

*Burn in period – 20,000 not acceptable; please do it with at least 100,000.*

**Response**
We thank the Editor for this recommendation. The original population-structure analysis was performed using STRUCTURE with a burn-in period of 20,000 iterations. However, the original STRUCTURE/STRUCTURE HARVESTER analysis environment is no longer accessible to us, and therefore we were unable to rerun the original STRUCTURE analysis using the recommended 100,000-iteration burn-in period.

To address this concern and independently reassess the population structure, we reanalyzed the genotype dataset using the sparse non-negative matrix factorization (sNMF) algorithm implemented in the LEA package in R. The analysis evaluated K values from 1 to 10, with five independent repetitions for each K, and cross-entropy was used to evaluate the fit of the alternative K values. The resulting ancestry estimates and inferred population structure were consistent with the pattern obtained from the original analysis. The Materials and Methods section has therefore been revised to describe the LEA/sNMF analysis, and the corresponding population-structure results have been updated accordingly.

**Comment 4**

*MCMC – 20,000 not acceptable; please do it with at least 100,000.*

**Response**
We appreciate the Editor's recommendation. Because the original STRUCTURE analysis environment is no longer accessible, we did not report a new STRUCTURE analysis with 100,000 MCMC iterations without being able to reproduce and verify the original analysis. Instead, we performed an independent population-structure analysis using the sNMF algorithm implemented in the LEA package. The sNMF analysis was conducted for K = 1–10, with five independent repetitions per K, and cross-entropy was used to assess the alternative population-structure models. The resulting ancestry coefficients and population-structure pattern were highly consistent with those obtained from the original analysis. We have therefore replaced the original STRUCTURE-based description with the reproducible LEA/sNMF analysis in the revised manuscript.

**Comment 5**

*What is "Harvester"?*

**Response**
Thank you for raising this point. STRUCTURE HARVESTER is a post-processing tool used to summarize and visualize STRUCTURE results and facilitate the evaluation of alternative numbers of clusters (K). Because the revised population-structure analysis was performed using the sNMF algorithm implemented in the LEA package and no longer relies on STRUCTURE HARVESTER, we have removed the term **"**Harvester**"** from the revised manuscript and replaced the corresponding description with the LEA/sNMF methodology.

**Comment 6**

*Table legends (bold).*

**Response**
Thank you. We have reformatted all table titles/legends in accordance with the requested journal formatting and ensured consistency throughout the manuscript. We also checked the placement and formatting of table titles and legends. PLOS ONE specifies that table titles are placed above the tables, while legends and footnotes are placed below them.

**Comment 7**

*Format references as per journal style.*

**Response**
Thank you for this comment. We carefully checked and reformatted the entire reference list according to the PLOS ONE reference style. We also checked the in-text citation numbering and the formatting of author names, journal titles, volume and page/article information, and DOI details where applicable.

We appreciate the Editor's constructive comments and recommendations, which have helped us improve the methodological clarity, consistency, and presentation of the manuscript.
