# Supplementary material for: Genetic diversity and population structure of early-maturing yellow and orange maize inbred lines for improved breeding applications
Source: PLoS One. 2026 Sep 25;21(9):e0358751. doi: 10.1371/journal.pone.0358751 (PMC13614566; doi:10.1371/journal.pone.0358751)
Supplement: Peer review history

# Peer review: Decision Letter 0

**DOI:** 10.1371/journal.pone.0358751.r001

**Authors:** Muhammad Abdul Rehman Rashid

28 Oct 2025

Dear Dr. Adejumobi,

Thank you for submitting your manuscript to PLOS ONE. After careful consideration, we feel that it has merit but does not fully meet PLOS ONE’s publication criteria as it currently stands. Therefore, we invite you to submit a revised version of the manuscript that addresses the points raised during the review process.

Please submit your revised manuscript by Dec 12 2025 11:59PM. If you will need more time than this to complete your revisions, please reply to this message or contact the journal office at plosone@plos.org. When you're ready to submit your revision, log on to https://www.editorialmanager.com/pone/ and select the 'Submissions Needing Revision' folder to locate your manuscript file.

- A rebuttal letter that responds to each point raised by the academic editor and reviewer(s). You should upload this letter as a separate file labeled 'Response to Reviewers'.
- A marked-up copy of your manuscript that highlights changes made to the original version. You should upload this as a separate file labeled 'Revised Manuscript with Track Changes'.
- An unmarked version of your revised paper without tracked changes. You should upload this as a separate file labeled 'Manuscript'.

If you would like to make changes to your financial disclosure, please include your updated statement in your cover letter. Guidelines for resubmitting your figure files are available below the reviewer comments at the end of this letter.

If applicable, we recommend that you deposit your laboratory protocols in protocols.io to enhance the reproducibility of your results. Protocols.io assigns your protocol its own identifier (DOI) so that it can be cited independently in the future. For instructions see: https://journals.plos.org/plosone/s/submission-guidelines#loc-laboratory-protocols. Additionally, PLOS ONE offers an option for publishing peer-reviewed Lab Protocol articles, which describe protocols hosted on protocols.io. Read more information on sharing protocols at https://plos.org/protocols?utm_medium=editorial-email&utm_source=authorletters&utm_campaign=protocols.

We look forward to receiving your revised manuscript.

Kind regards,

Muhammad Abdul Rehman Rashid, PhD

Academic Editor

PLOS ONE

Journal Requirements:

When submitting your revision, we need you to address these additional requirements.

1. Please ensure that your manuscript meets PLOS ONE's style requirements, including those for file naming. The PLOS ONE style templates can be found at

https://journals.plos.org/plosone/s/file?id=wjVg/PLOSOne_formatting_sample_main_body.pdf and

https://journals.plos.org/plosone/s/file?id=ba62/PLOSOne_formatting_sample_title_authors_affiliations.pdf

2. Please note that PLOS One has specific guidelines on code sharing for submissions in which author-generated code underpins the findings in the manuscript. In these cases, we expect all author-generated code to be made available without restrictions upon publication of the work. Please review our guidelines at https://journals.plos.org/plosone/s/materials-and-software-sharing#loc-sharing-code and ensure that your code is shared in a way that follows best practice and facilitates reproducibility and reuse.

3. Thank you for stating the following financial disclosure:

“Bill and Melinda Gates Foundation through the Accelerated Genetic Gain in Maize and Wheat Project [OPP1134248]”

Please state what role the funders took in the study. If the funders had no role, please state: "The funders had no role in study design, data collection and analysis, decision to publish, or preparation of the manuscript."

If this statement is not correct you must amend it as needed.

Please include this amended Role of Funder statement in your cover letter; we will change the online submission form on your behalf.

4. When completing the data availability statement of the submission form, you indicated that you will make your data available on acceptance. We strongly recommend all authors decide on a data sharing plan before acceptance, as the process can be lengthy and hold up publication timelines. Please note that, though access restrictions are acceptable now, your entire data will need to be made freely accessible if your manuscript is accepted for publication. This policy applies to all data except where public deposition would breach compliance with the protocol approved by your research ethics board. If you are unable to adhere to our open data policy, please kindly revise your statement to explain your reasoning and we will seek the editor's input on an exemption. Please be assured that, once you have provided your new statement, the assessment of your exemption will not hold up the peer review process.

5. Please include captions for your Supporting Information files at the end of your manuscript, and update any in-text citations to match accordingly. Please see our Supporting Information guidelines for more information: http://journals.plos.org/plosone/s/supporting-information.

6. If the reviewer comments include a recommendation to cite specific previously published works, please review and evaluate these publications to determine whether they are relevant and should be cited. There is no requirement to cite these works unless the editor has indicated otherwise.

[Note: HTML markup is below. Please do not edit.]

Reviewers' comments:

Reviewer's Responses to Questions

**Comments to the Author**

1. Is the manuscript technically sound, and do the data support the conclusions?

Reviewer #1: Yes

Reviewer #2: No

**********

2. Has the statistical analysis been performed appropriately and rigorously?

Reviewer #1: Yes

Reviewer #2: No

**********

3. Have the authors made all data underlying the findings in their manuscript fully available?

The PLOS Data policy

Reviewer #1: Yes

Reviewer #2: Yes

**********

4. Is the manuscript presented in an intelligible fashion and written in standard English?

Reviewer #1: Yes

Reviewer #2: No

**********

Reviewer #1: - Unlocking Climate-Resilient and Nutritive Maize Production in Africa: is it necessary to include this in the title? “Genetic Diversity and Population Structure of Early-Maturing Yellow and Orange Inbred Lines” captures well, what this study is about. I will suggest you delete the first part of the title.

- Second paragraph: IITA should be written in full- first time appearing in the manuscript.

- Several studies have employed SNP markers to assess genetic diversity and to understand the population structure in the IITA maize germplasm but cited only two studies….. Please provide at least three references.

The objectives of this study are to assess the genetic diversity and patterns of the population structure of recently developed early carotenoid maize inbred lines of the IITA maize improvement program and classify the lines into distinct heterotic groups

- Replace ‘are” with were

- You suddenly introduced “carotenoids” to describe the lines you are examining. At no point in the introduction did you mention that the yellow and orange inbred lines contain carotenoids. This may be confusing to readers who are not maize breeders. Please indicate this in the introduction.

At the S4 stage of inbreeding, 250–300 selected lines were crossed to broad-based testers to estimate the GCA of the lines.

- First use of GCA, please write in full.

- Previous studies (Adu et al., 2019) have reported similar findings using DArT-derived SNP markers in tropical maize populations. Only one study cited here, recast the sentence to reflect one study e.g. ‘An earlier study reported similar findings….”

Reference :

Baffour Badu-Apraku, Morakinyo A.B. Fakorede, Benjamin Annor, Gloria B. Adu, Ebenezer Obeng-Bio,

Pearl Abu, Olatunde A. Bhadmus, Charles Nelimor. (2023). Genetic Enhancement of Early and Extra-

Early Maturing Maize for Tolerance to Low-Soil Nitrogen in Sub-Saharan Africa. Crop Breeding,

Genetics and Genomics. https://doi.org/10.20900/cbgg20230001 . this is inconsistent with other references. It should be ‘Badu-Apraku, B., Fakorade, M.A.B, Annor, B., Adu G.B., Obeng-Bio, E., Abu, P.,Bhadmus O.A.,& Nelimor, C. (2023)………….

Reviewer #2: 1. General Comments

The manuscript explores genetic diversity and population structure of 376 maize inbred lines using 1,954 DArTseq SNPs. However, the study is largely descriptive and does not justify its broader claims of “unlocking climate resilience” or “nutritive maize production.” No phenotypic, nutritional, or climate-related data were included to support these assertions.

Moreover, the analyses presented (STRUCTURE, PCA, AMOVA) are standard population genetic tools but are not novel or sufficiently interpreted to offer biological or breeding insights. Figures are of poor visual quality, and the discussion section lacks critical comparison with the broader maize diversity literature (e.g., CIMMYT or USDA datasets).

The manuscript would require substantial restructuring, analytical improvement, and data-supported interpretation before being reconsidered for publication.

2. Major Comments

2.1. Misalignment Between Title, Objectives, and Data

The title emphasizes “climate resilience” and “nutritive maize,” but the study only includes molecular marker data.

No environmental, stress tolerance, or carotenoid content data are presented.

Conclusions on climate resilience and nutrition are speculative and unsupported.

Recommendation:

Reframe the title to reflect the true scope (“Genetic Diversity and Population Structure of Early-Maturing Yellow and Orange Maize Inbred Lines in West Africa”) or integrate phenotypic and biochemical data that substantiate these claims.

2.2. Lack of Clear Hypothesis or Research Questions

The introduction provides background but fails to define a clear scientific hypothesis or rationale.

The study’s objective should specify why genetic diversity of these particular lines matters for breeding or adaptation.

Recommendation:

Formulate explicit research questions (“Do yellow and orange early-maturing maize lines form distinct heterotic groups relevant to provitamin-A breeding?”).

2.3. Weak Methodological Description

There is insufficient detail on the origin, grouping, and phenotypic characteristics of the 376 inbreds.

Marker filtering criteria (MAF thresholds, missing data limits) are presented without justification.

No reference to population validation, such as comparison to pedigree or combining ability data.

Recommendation:

Include a detailed table summarizing the genetic material (population source, kernel color, geographic origin). Justify marker selection parameters and provide evidence of quality control.

2.4. Poor Statistical and Biological Interpretation

STRUCTURE and DAPC results are presented without discussing biological meaning (whether clusters relate to endosperm color, breeding history, or regional adaptation).

AMOVA and FST values are interpreted superficially. No tests for significance or bootstrapping are reported.

Claims of heterotic grouping are not supported by hybrid testing or field validation.

Recommendation:

Include clearer interpretation linking clusters to biological or breeding context. Validate results against known heterotic groups or agronomic performance data.

2.5. Discussion is Superficial

The discussion repeats results rather than analyzing them in the context of previous studies.

No meaningful linkage is made to breeding for resilience, nutrition, or early maturity.

References are overly narrow (mostly from IITA-based studies).

Recommendation:

Substantially revise the discussion to:

Compare results to other African maize diversity studies (IMMYT, WEMA).

Highlight implications for breeding strategies, not just descriptive diversity.

Remove generalized statements on food security or poverty alleviation unless supported by data.

2.6. Figures and Tables

Figures are low resolution and poorly formatted. Axes labels, legends, and color schemes are unclear.

Some plots (PCA, STRUCTURE barplots) appear pixelated or compressed.

Supplementary figures are referenced inconsistently in the text.

Recommendation:

Recreate figures using high-quality plotting tools (R/ggplot2). Ensure all labels and legends are legible and that supplementary materials are properly cross-referenced.

2.7. Language and Style

The manuscript contains grammatical errors and awkward phrasing throughout.

Verb tense and article use are inconsistent.

Several sentences are unscientific or overly promotional (“unlocking resilience,” “revolutionizing maize production”).

Recommendation:

Seek professional English editing to improve readability and scientific tone.

3. Minor Comments

Define all abbreviations at first mention (e.g., AMOVA, PCA, SNP, DArTseq).

Ensure consistent citation style (author–year format).

Clarify whether marker data and accession metadata are publicly available.

Review and correct reference formatting errors.

Revise abstract for conciseness and alignment with objectives.

**********

what does this mean?). If published, this will include your full peer review and any attached files.

If you choose “no”, your identity will remain anonymous but your review may still be made public.

**Do you want your identity to be public for this peer review?**  For information about this choice, including consent withdrawal, please see our Privacy Policy

Reviewer #1: No

Reviewer #2: No

**********

[NOTE: If reviewer comments were submitted as an attachment file, they will be attached to this email and accessible via the submission site. Please log into your account, locate the manuscript record, and check for the action link "View Attachments". If this link does not appear, there are no attachment files.]

To ensure your figures meet our technical requirements, please review our figure guidelines: https://journals.plos.org/plosone/s/figures

You may also use PLOS’s free figure tool, NAAS, to help you prepare publication quality figures: https://journals.plos.org/plosone/s/figures#loc-tools-for-figure-preparation.

NAAS will assess whether your figures meet our technical requirements by comparing each figure against our figure specifications.

# Author comment: Author response to Decision Letter 1

**DOI:** 10.1371/journal.pone.0358751.r002

4 Dec 2025

Response to Reviewers’ comments on the manuscript PONE-D-25-49435

Reviewer #1: - Unlocking Climate-Resilient and Nutritive Maize Production in Africa: is it necessary to include this in the title? “Genetic Diversity and Population Structure of Early-Maturing Yellow and Orange Inbred Lines” captures well, what this study is about. I will suggest you delete the first part of the title.

Response:

Thank you very much for this valuable comment. We fully agree that the title should accurately reflect the scope of the study. Following this suggestion and consistent with comments from another reviewer we have revised the title to focus more clearly on the scientific content of the manuscript. However, instead of completely removing the broader breeding relevance, we opted for a concise and balanced title that aligns with the specific analyses presented while still acknowledging the intended application of the findings. The revised title is now “Genetic Diversity and Population Structure of Early-Maturing Yellow and Orange Maize Inbred Lines for Improved Breeding Applications.” We believe this version appropriately centers the study’s technical focus while remaining scientifically accurate and acceptable to all reviewer concerns.

- Second paragraph: IITA should be written in full- first time appearing in the manuscript.

Response

Thank you for the observation. IITA has been written in full as requested.

- Several studies have employed SNP markers to assess genetic diversity and to understand the population structure in the IITA maize germplasm but cited only two studies….. Please provide at least three references.

Response

We have included a third reference to the two originally presented to complete three references as requested.

The objectives of this study are to assess the genetic diversity and patterns of the population structure of recently developed early carotenoid maize inbred lines of the IITA maize improvement program and classify the lines into distinct heterotic groups

- Replace ‘are” with were

- You suddenly introduced “carotenoids” to describe the lines you are examining. At no point in the introduction did you mention that the yellow and orange inbred lines contain carotenoids. This may be confusing to readers who are not maize breeders. Please indicate this in the introduction.

Response

Thank you for the observation. We have changed “are” to “were” as requested. Regarding the term “carotenoid,” we agree that its introduction without prior context could cause confusion. We have therefore replaced “carotenoid” with “yellow and orange kernel,” since only about one-third of the 376 inbred lines have been phenotyped for carotenoid content. This adjustment ensures consistency with the information presented in the Introduction and avoids implying that all lines had confirmed carotenoid measurements.

At the S4 stage of inbreeding, 250–300 selected lines were crossed to broad-based testers to estimate the GCA of the lines.

- First use of GCA, please write in full.

- Previous studies (Adu et al., 2019) have reported similar findings using DArT-derived SNP markers in tropical maize populations. Only one study cited here, recast the sentence to reflect one study e.g. ‘An earlier study reported similar findings….”

Response

Thank you for the observation. We have GCA in full as requested. In addition, we have modified the statement to be ‘An earlier study reported similar findings….” as suggested.

Reference :

Baffour Badu-Apraku, Morakinyo A.B. Fakorede, Benjamin Annor, Gloria B. Adu, Ebenezer Obeng-Bio,

Pearl Abu, Olatunde A. Bhadmus, Charles Nelimor. (2023). Genetic Enhancement of Early and Extra-

Early Maturing Maize for Tolerance to Low-Soil Nitrogen in Sub-Saharan Africa. Crop Breeding,

Genetics and Genomics. https://doi.org/10.20900/cbgg20230001 . this is inconsistent with other references. It should be ‘Badu-Apraku, B., Fakorade, M.A.B, Annor, B., Adu G.B., Obeng-Bio, E., Abu, P.,Bhadmus O.A.,& Nelimor, C. (2023)………….

Response

Thank you for the observation. We have corrected the citation as suggested in the revised manuscript.

Reviewer #2: 1. General Comments

The manuscript explores genetic diversity and population structure of 376 maize inbred lines using 1,954 DArTseq SNPs. However, the study is largely descriptive and does not justify its broader claims of “unlocking climate resilience” or “nutritive maize production.” No phenotypic, nutritional, or climate-related data were included to support these assertions.

Moreover, the analyses presented (STRUCTURE, PCA, AMOVA) are standard population genetic tools but are not novel or sufficiently interpreted to offer biological or breeding insights. Figures are of poor visual quality, and the discussion section lacks critical comparison with the broader maize diversity literature (e.g., CIMMYT or USDA datasets). The manuscript would require substantial restructuring, analytical improvement, and data-supported interpretation before being reconsidered for publication.

Response:

We sincerely appreciate the reviewer’s thoughtful observations concerning the scope and interpretation of our study. Our intention in referring to “unlocking climate-resilient and nutritive maize production” was not to imply that this single study directly evaluates climate- or nutrition-related phenotypes. Rather, the phrase was meant to contextualize the importance of developing early-maturing yellow and orange inbred lines, which form a key foundation for breeding climate-resilient and biofortified hybrids targeted to stress-prone environments. Nevertheless, we agree that such wording may overextend what the current dataset directly supports. To address this concern, we have revised the manuscript topic to clarify the contribution of our work, emphasizing that the study provides genetic diversity and population structure insights, which are essential inputs for future breeding pipelines targeting climate resilience and nutritional improvement.

Regarding the point that the analyses are descriptive, we have strengthened the biological and breeding relevance of our findings by expanding the interpretation of STRUCTURE, PCA, AMOVA, and phylogenetic results to explain how the observed patterns can guide heterotic grouping and parental selection in breeding programs. Compare our diversity levels with those reported in CIMMYT, USDA, and other global maize panels, thereby situating our findings within the broader context of maize germplasm diversity. We have equally provided the improved version of the figures in TIFF format to enhance clarity and readability.

While the analytical tools used (STRUCTURE, PCA, AMOVA) are standard in population genetic studies, they remain essential and widely validated methods for initial characterization of breeding materials. Our contribution lies in applying these tools to a newly developed set of early-maturing yellow and orange inbred lines, which have not previously been characterized and represent an important resource for maize improvement efforts.

2. Major Comments

2.1. Misalignment Between Title, Objectives, and Data

The title emphasizes “climate resilience” and “nutritive maize,” but the study only includes molecular marker data.

No environmental, stress tolerance, or carotenoid content data are presented.

Conclusions on climate resilience and nutrition are speculative and unsupported.

Recommendation:

Reframe the title to reflect the true scope (“Genetic Diversity and Population Structure of Early-Maturing Yellow and Orange Maize Inbred Lines in West Africa”) or integrate phenotypic and biochemical data that substantiate these claims.

Response

We agree with the reviewer that the original title and conclusions maybe speculative, as the manuscript focused on molecular marker data (Diversity, Population Structure, and Heterotic groupings) and lacked the necessary phenotypic data (stress tolerance, yield, and carotenoid content) to support claims of "climate resilience" and "nutritive maize." To achieve alignment and maintain the current scope of the study, we have implemented the following changes throughout the manuscript. The title has been revised to "Genetic Diversity and Population Structure of Early-Maturing Yellow and Orange Maize Inbred Lines for Improved Breeding Applications." This title accurately reflects the use of molecular markers to characterize the population without making claims about performance or end-product quality. All objectives related to quantifying 'climate resilience' or 'nutritive value' have been modified to focus on the genetic characterization of the germplasm. The Discussion and Conclusion sections have been thoroughly revised to focus only on inferences for breeding strategy (e.g., selection of diverse parents, management of sub-populations, and marker-assisted selection potential), which are directly supported by the SNP marker data presented.

2.2. Lack of Clear Hypothesis or Research Questions

The introduction provides background but fails to define a clear scientific hypothesis or rationale.

The study’s objective should specify why genetic diversity of these particular lines matters for breeding or adaptation.

Recommendation:

Formulate explicit research questions (“Do yellow and orange early-maturing maize lines form distinct heterotic groups relevant to provitamin-A breeding?”).

Response

Thank you for this helpful comment. We agree that the introduction required a clearer rationale and framing of the scientific questions guiding the study. In the revised manuscript, we have substantially rewritten the introduction to better articulate why assessing genetic diversity and population structure in these recently developed early-maturing yellow and orange maize inbred lines is important for strengthening hybrid development in West and Central Africa. Specifically, the revised introduction now clarifies the breeding context that these lines represent new early-maturing donor materials derived from IITA breeding pipelines targeting stress-prone savannas. Also, explains the relevance of genetic diversity and structure for parental selection, hybrid formation, and maintenance of heterotic patterns within early-maturing maize programs. Finally, introduces explicit research questions that directly reflect the focus of this study. These include whether the yellow and orange early-maturing lines show clear genetic clustering, whether such clustering corresponds to usable heterotic groups for hybrid development, and how molecular diversity patterns can inform future line development and tester allocation.

2.3. Weak Methodological Description

There is insufficient detail on the origin, grouping, and phenotypic characteristics of the 376 inbreds.

Marker filtering criteria (MAF thresholds, missing data limits) are presented without justification.

No reference to population validation, such as comparison to pedigree or combining ability data.

Recommendation:

Include a detailed table summarizing the genetic material (population source, kernel color, geographic origin). Justify marker selection parameters and provide evidence of quality control.

Response

Thank you for pointing this out. We have included the recommended table on population source, kernel color, and geographic origin. Additionally, we have now added justification for each filtering threshold in the revised manuscript. The criteria we followed were established quality-control practices for DArTseq and GBS-based diversity studies in maize and other crops, and they ensure that the final marker set is both informative and reliable for downstream analyses.

2.4. Poor Statistical and Biological Interpretation

STRUCTURE and DAPC results are presented without discussing biological meaning (whether clusters relate to endosperm color, breeding history, or regional adaptation).

AMOVA and FST values are interpreted superficially. No tests for significance or bootstrapping are reported.

Claims of heterotic grouping are not supported by hybrid testing or field validation.

Recommendation:

Include clearer interpretation linking clusters to biological or breeding context. Validate results against known heterotic groups or agronomic performance data.

Response

We appreciate the reviewer’s thoughtful comments. In the revised manuscript, we have substantially improved the interpretation of the STRUCTURE, DAPC, PCA, and AMOVA results by linking the clustering patterns to the biological and breeding context of the IITA early-maturing maize program. We now clarify that the two genetic clusters largely reflect differences in breeding history and germplasm sources, rather than kernel color or ecological adaptation. The AMOVA results have been reanalyzed and now include significance tests of variance components, and pairwise FST values are interpreted more comprehensively. We have also added a clear explanation that the genetic clusters represent preliminary heterotic grouping patterns and not confirmed heterotic pools, given the absence of hybrid testcross data.

2.5. Discussion is Superficial

The discussion repeats results rather than analyzing them in the context of previous studies.

No meaningful linkage is made to breeding for resilience, nutrition, or early maturity.

References are overly narrow (mostly from IITA-based studies).

Recommendation:

Substantially revise the discussion to:

Compare results to other African maize diversity studies (CIMMYT, WEMA).

Highlight implications for breeding strategies, not just descriptive diversity.

Remove generalized statements on food security or poverty alleviation unless supported by data.

Response

Thank you for the comment. We have made substantial revision to the Discussion section to enhance its scholarly and practical merit. The core changes involved removing repetitive restatements of results and replacing them with analytical interpretation. To better contextualize the findings, the discussion now incorporates comparisons with a broader range of African maize studies (including CIMMYT and WEMA research) and utilizes a diversified reference base. Furthermore, the revised section strengthens its relevance by explicitly linking the observed genetic clusters and diversity patterns to key breeding priorities, such as developing drought resilience, improving nutritional quality (QPM/provitamin-A), and facilitating early-maturity adaptation. Finally, the authors ensured scientific focus by removing generalized socio-economic claims and added a dedicated subsection outlining how the findings can directly guide future parental selection and hybrid development strategies.

2.6. Figures and Tables

Figures are low resolution and poorly formatted. Axes labels, legends, and color schemes are unclear.

Some plots (PCA, STRUCTURE barplots) appear pixelated or compressed.

Supplementary figures are referenced inconsistently in the text.

Recommendation:

Recreate figures using high-quality plotting tools (R/ggplot2). Ensure all labels and legends are legible and that supplementary materials are properly cross-referenced.

Response

We have recreated the Figures and uploaded the TIFF format alongside the revised version of the manuscript. Supplementary materials have been cross-referenced as requested.

2.7. Language and Style

The manuscript contains grammatical errors and awkward phrasing throughout.

Verb tense and article use are inconsistent.

Several sentences are unscientific or overly promotional (“unlocking resilience,” “revolutionizing maize production”).

Recommendation:

Seek professional English editing to improve readability and scientific tone.

Response

Thank you for the comment. We have revised the manuscript for grammatical correctness and sentence structure as requested.

3. Minor Comments

Define all abbreviations at first mention (e.g., AMOVA, PCA, SNP, DArTseq).

Ensure consistent citation style (author–year format).

Clarify whether marker data and accession metadata are publicly available.

Review and correct reference formatting errors.

Revise abstract for conciseness and alignment with objectives.

Response

All abbreviations have been interpreted, citations corrected with Mendeley software, data availability clarified, and abstract revised as suggested.

# Peer review: Decision Letter 1

**DOI:** 10.1371/journal.pone.0358751.r003

**Authors:** Sarah Jose

20 Apr 2026

Dear Dr. Adejumobi,

Please submit your revised manuscript by Jun 04 2026 11:59PM. If you will need more time than this to complete your revisions, please reply to this message or contact the journal office at plosone@plos.org. When you're ready to submit your revision, log on to https://www.editorialmanager.com/pone/ and select the 'Submissions Needing Revision' folder to locate your manuscript file.

- A letter that responds to each point raised by the academic editor and reviewer(s). You should upload this letter as a separate file labeled 'Response to Reviewers'.
- A marked-up copy of your manuscript that highlights changes made to the original version. You should upload this as a separate file labeled 'Revised Manuscript with Track Changes'.
- An unmarked version of your revised paper without tracked changes. You should upload this as a separate file labeled 'Manuscript'.

If you would like to make changes to your financial disclosure, please include your updated statement in your cover letter. Guidelines for resubmitting your figure files are available below the reviewer comments at the end of this letter.

If applicable, we recommend that you deposit your laboratory protocols in protocols.io to enhance the reproducibility of your results. Protocols.io assigns your protocol its own identifier (DOI) so that it can be cited independently in the future. For instructions see: https://journals.plos.org/plosone/s/submission-guidelines#loc-laboratory-protocols. Additionally, PLOS ONE offers an option for publishing peer-reviewed Lab Protocol articles, which describe protocols hosted on protocols.io. Read more information on sharing protocols at https://plos.org/protocols?utm_medium=editorial-email&utm_source=authorletters&utm_campaign=protocols.

We look forward to receiving your revised manuscript.

Kind regards,

Sarah Jose

Staff Editor

PLOS One

Journal Requirements:

If the reviewer comments include a recommendation to cite specific previously published works, please review and evaluate these publications to determine whether they are relevant and should be cited. There is no requirement to cite these works unless the editor has indicated otherwise.

[Note: HTML markup is below. Please do not edit.]

Reviewers' comments:

Reviewer's Responses to Questions

**Comments to the Author**

Reviewer #3: All comments have been addressed

Reviewer #4: (No Response)

Reviewer #5: (No Response)

Reviewer #6: (No Response)

**********

2. Is the manuscript technically sound, and do the data support the conclusions?

Reviewer #3: Yes

Reviewer #4: No

Reviewer #5: Partly

Reviewer #6: Yes

**********

3. Has the statistical analysis been performed appropriately and rigorously?

Reviewer #3: Yes

Reviewer #4: Yes

Reviewer #5: Yes

Reviewer #6: Yes

**********

4. Have the authors made all data underlying the findings in their manuscript fully available?

The PLOS Data policy

Reviewer #3: Yes

Reviewer #4: Yes

Reviewer #5: Yes

Reviewer #6: Yes

**********

5. Is the manuscript presented in an intelligible fashion and written in standard English?

Reviewer #3: Yes

Reviewer #4: Yes

Reviewer #5: Yes

Reviewer #6: Yes

**********

Reviewer #3: This investigation must show a brief notice about grain yield and quality for chosen these lines and not only early maturity because sometimes early maturity line have poor yield and quality so please i need short note on this point to complete the idea of this research. Also, to reveal if this lines have economic values or not to plant them

Reviewer #4: Journal: PLOS One

Manuscript ID: PONE-D-25-49435R1

Manuscript Title: Genetic Diversity and Population Structure of Early-Maturing Yellow and Orange Maize Inbred Lines for Improved Breeding Applications

Authors: Neo et al.,

Date: 2/6/2026

The authors have presented the genetic diversity, population structure, and inferred heterotic grouping of early-maturing yellow and orange maize inbred lines developed within the IITA Maize Improvement Program. They have genotype 376 elite inbred lines using DArTseq markers and applies standard population genetic approaches. The work addresses an important objective for tropical maize breeding by providing genomic characterization of germplasm intended for drought-prone environments in sub-Saharan Africa, with clear relevance to hybrid development and germplasm management.

Overall, the manuscript presents a substantial dataset and applies appropriate analytical approaches for germplasm characterization. The results are generally consistent with expectations for elite tropical maize populations subjected to recurrent selection, and the study has potential value for breeding programs seeking to optimize parental selection and maintain genetic diversity. However, several aspects of interpretation and presentation require revision before the manuscript is suitable for publication. The authors should rewrite certain sections and restrict to the molecular findings instead of broader applications.

Major concerns

The authors interpreting the molecular clusters derived from SNP data as functional heterotic groups and implies direct applicability for hybrid prediction. However, heterotic grouping in maize breeding is typically validated through combining ability or hybrid performance data. In the absence of testcross or phenotypic validation, the identified groups should be described as putative or molecularly inferred clusters rather than confirmed heterotic groups. The conclusions should be moderated accordingly throughout the manuscript.

The LD analysis by the authors has merit, but the discussion extends beyond the data by making claims regarding suitability for association mapping and genomic selection efficiency. These implications are speculative without accompanying phenotypic or prediction analyses and should be reframed as potential applications rather than demonstrated outcomes.

The authors should rewrite some sections in the manuscript. The authors refer to drought tolerance, Striga resistance, and nutritional improvement, no phenotypic analyses are included. As a result, several conclusions linking genetic diversity to adaptive or agronomic value are inferential. The manuscript would benefit from clearer separation between genomic characterization results and breeding implications that remain hypothetical.

The greedy algorithm approach for core set identification is acceptable, but the manuscript does not sufficiently demonstrate that the selected subset adequately preserves diversity relative to the full panel. Quantitative comparisons of diversity parameters and explicit allele-retention metrics should be provided to support claims of representativeness.

The alignment between molecular clustering and pedigree history is expected given the breeding origins of the materials and should not be presented as independent biological validation of heterotic patterns. This argument should be moderated to avoid circular reasoning.

Thank you,

Reviewer #5: This manuscript presents data that may be of interest to the field and fits within the scope of PLOS ONE. However, the current version requires major revisions before it can be considered for publication.

In particular, greater clarity is needed in the description of the methodology and statistical analyses to ensure reproducibility. Additionally, some interpretations in the Discussion should be more closely aligned with the presented results. Addressing these issues will substantially improve the scientific rigor and transparency of the manuscript.

Reviewer #6: The manuscript “Genetic Diversity and Population Structure of Early-Maturing Yellow and Orange Maize Inbred Lines for Improved Breeding Applications” reports on genetic diversity and population structure analyses in a collection of 376 African maize inbreed lines based on DArTag genotyping.

The manuscript is fairly well written, the objectives are clear and the data analysis was carried out using widely accepted approaches there are several issues which should be addressed before publication

1. Genotyping is not described in sufficient detail. Author report that DArTag was used.

Please provide a more detailed description of the method and, if possible, a reference.

At the moment it is not clear if it is a NGS- based method, and array-based method or a different genotyping approach. .

Also: I assume a maize reference genome was used in data analysis, please describe precisely, which genome, where it is available.

2. Experimental location: please provide country name (after “IITA-Ibadan”)

3. Table1. The last column is not informative, and can be removed. This information can be given in the title of the Table 1

4. Supplementary Tables 1 to 3 should be combined into one table to facilitate comparisons

5. Please provide references for several generalised statements, such as:

“A maximum missing data rate of 20% was used to retain informative loci while avoiding excessive

imputation, consistent with thresholds commonly applied in maize DArTseq and GBS datasets”

and

“This approach is widely used for maximizing allelic diversity because it is computationally

efficient and approximates the optimal solution for allelic coverage”

also

“Following common practice in germplasm optimization, the target core size was set

to 15% of the total collection”

and

“Using the commonly applied threshold of r² = 0.1”

Another example:

“Similar alignment between population structure and pedigree has been observed in CIMMYT early-drought panels and WEMA drought-tolerant hybrids, where genetic clustering often reflects long-term tester usage and recurrent selection pipelines”

6. What tool/algorithm was used to select core set entries? Please provide a reference or a link

7. Where is the list of inbreed lines constituting the core set

8. Please provide the reference for the Harvester tool

9. What tool was used to perform DAPC

10. As far as I can understand the heterotic groups were identified based on the results of phylogenetic analysis. Why? How do the results of this analysis correspond to Structure and DAPC outcomes? Please indicate this in the Figures (for example by colours – use in the DAPC plot colours that reflect the population assignment of inbreds to subpopulations resulting from Structure analysis, and use a similar approach to show how clusters in the dendrograms correspond to the subpopulations identified by Structure). If it was already done, please provide a clearer and more detailed description. Please describe the comparison of the results of these three approaches in the Results section, including a comparison to the pedigree information, I could only notice one sentence on this subject (comparison of the outcomes of different approaches used) in the Conclusions

11. What is the order of the inbreds in the Structure plot.

**********

what does this mean?). If published, this will include your full peer review and any attached files.

If you choose “no”, your identity will remain anonymous but your review may still be made public.

**Do you want your identity to be public for this peer review?**  For information about this choice, including consent withdrawal, please see our Privacy Policy

Reviewer #3: No

Reviewer #4: **Yes:** Venkatesh Bollina

Reviewer #5: No

Reviewer #6: No

**********

[NOTE: If reviewer comments were submitted as an attachment file, they will be attached to this email and accessible via the submission site. Please log into your account, locate the manuscript record, and check for the action link "View Attachments". If this link does not appear, there are no attachment files.]

To ensure your figures meet our technical requirements, please review our figure guidelines: https://journals.plos.org/plosone/s/figures

You may also use PLOS’s free figure tool, NAAS, to help you prepare publication quality figures: https://journals.plos.org/plosone/s/figures#loc-tools-for-figure-preparation.

NAAS will assess whether your figures meet our technical requirements by comparing each figure against our figure specifications.

# Author comment: Author response to Decision Letter 2

**DOI:** 10.1371/journal.pone.0358751.r004

11 Jun 2026

Response to Reviewers

Manuscript Title: Genetic Diversity and Population Structure of Early-Maturing Yellow and Orange Maize Inbred Lines for Improved Breeding Applications

Dear Editor and Reviewers,

We sincerely thank the Editor and the Reviewers for their thorough evaluation of our manuscript and for the constructive comments and suggestions. We have carefully revised the manuscript and believe that the changes have substantially improved its scientific rigor, clarity, and reproducibility.

All reviewer comments have been addressed point-by-point below. Reviewer comments are shown in italics, followed by our responses.

REVIEWER 3

Comment 1:

"The manuscript lacks sufficient information on grain yield performance and quality characteristics of the germplasm."

Response:

We thank the reviewer for this important observation. The primary objective of the study was genomic characterization of the inbred lines rather than phenotypic evaluation. Nevertheless, we have clarified in the Discussion section that the evaluated inbred lines were previously developed and selected within the IITA Maize Improvement Program under recurrent screening for grain yield performance, stress tolerance, and kernel quality traits. This addition emphasizes the breeding relevance of the germplasm analyzed in the present study.

Revision made: A new paragraph was added in the Breeding Implications and Future Directions section describing the agronomic relevance and breeding history of the evaluated germplasm.

REVIEWER 4

Comment 1:

"The manuscript overinterprets molecular clustering as evidence of validated heterotic groups."

Response:

We appreciate this observation and agree that molecular clustering alone cannot conclusively validate heterotic groups. Throughout the manuscript, terminology was revised to avoid overstatement. References to "confirmed heterotic groups" were replaced with "putative heterotic groups," "molecular clusters," or "putative molecular heterotic groups" where appropriate.

Revision made: Changes were implemented throughout the Abstract, Results, Discussion, and Conclusions.

Comment 2:

"Claims regarding hybrid prediction and tester selection are not sufficiently supported."

Response:

We agree and have moderated these statements throughout the manuscript. Previous wording implying direct hybrid prediction or validated tester assignment has been replaced with more cautious language indicating potential utility for future hybrid evaluation and tester development studies.

Revision made: Relevant sections in the Results, Discussion, and Breeding Implications sections were revised accordingly.

Comment 3:

"Biological validation of the identified groups is not demonstrated."

Response:

We thank the reviewer for this important clarification. Statements referring to "biological validation" were removed. These were replaced with wording indicating consistency between pedigree background and molecular clustering patterns rather than direct biological validation.

Revision made: The Relationship Between Source Populations and Putative Heterotic Groups section and associated discussion were revised.

Comment 4:

"The discussion of linkage disequilibrium extends beyond the evidence presented."

Response:

We agree. The LD discussion has been revised to avoid unsupported implications regarding QTL detection efficiency, GWAS performance, and genomic selection effectiveness. The revised discussion now focuses on historical recombination patterns and potential utility for future genetic studies.

Revision made: The Linkage Disequilibrium and Recombination History section was substantially revised.

Comment 5:

"The core set does not adequately demonstrate preservation of diversity."

Response:

We thank the reviewer for this suggestion. Additional analyses were conducted to compare the full collection and the candidate core subsets using allelic richness, expected heterozygosity, observed heterozygosity, Shannon diversity index, mean genetic distance, and PCA-based diversity space coverage. These analyses demonstrate the effectiveness of the proposed core subset in preserving genetic diversity.

Revision made: A new section entitled "Core Subset Selection and Diversity Characterization" was added, together with Table 6 and Figures 8–9.

REVIEWER 5

Comment 1:

"The methodology lacks sufficient detail for reproducibility."

Response:

We appreciate this observation and have substantially expanded the Materials and Methods section. Additional details regarding marker quality control, population structure analysis, LD analysis, phylogenetic analysis, and core set selection have been incorporated.

Revision made: Methods section expanded throughout.

Comment 2:

"Clarify the procedures used for population structure analysis."

Response:

Additional details regarding STRUCTURE, DAPC, cluster assignment criteria, Evanno method implementation, and phylogenetic reconstruction were added.

Revision made: Population structure methodology was revised and expanded.

REVIEWER 6

Comment 1:

"Provide a clearer description of the genotyping platform."

Response:

We thank the reviewer for identifying this important issue. The genotyping procedure was completely revised to clearly describe the DArTag platform, distinguish it from DArTseq, explain the targeted SNP approach, and specify the maize reference genome (B73 RefGen_v5) used during SNP calling.

Revision made: The Leaf Sample Collection and Genotyping Procedure section was extensively revised.

Comment 2:

"Provide references supporting filtering thresholds and analytical procedures."

Response:

Relevant references supporting marker quality control thresholds, LD analysis, population structure analysis, and core collection development were added throughout the Methods section.

Revision made: Additional citations have been incorporated throughout the Materials and Methods section.

Comment 3:

"Clarify the software and algorithms used for core set identification."

Response:

Additional details regarding CoreHunter 3, the simulated annealing optimization procedure, the stratified CoreHunter approach, and the greedy stepwise heuristic algorithm were added. Evaluation metrics used to compare core sets were also clearly described.

Revision made: The core collection methodology was rewritten and expanded substantially.

Comment 4:

"Provide information regarding marker numbers before and after filtering."

Response:

The manuscript now clearly reports the initial DArTag dataset of 3,305 SNP markers and the final filtered dataset of 1,954 high-quality SNP markers used for downstream analyses.

Revision made: Clarified in both the Methods and Results sections.

EDITORIAL CHANGES

In addition to the reviewer-specific revisions, we have:

i. Standardized terminology throughout the manuscript.

ii. Replaced overinterpreted statements with more cautious and evidence-based language.

iii. Corrected typographical and grammatical errors.

iv. Harmonized SNP numbers across all sections.

v. Updated figure captions and table descriptions.

vi. Improved consistency between Results and Discussion sections.

vii. Expanded the discussion of the practical implications of the identified core subset for tropical maize breeding.

We sincerely thank the Editor and Reviewers for their valuable comments and suggestions. We believe the revised manuscript has been substantially improved and hope it is now suitable for publication.

Sincerely,

Idris Ishola Adejumobi (On behalf of all Authors)

Attachment Submitted filename: Response to Reviewers-Second revision.docx

# Peer review: Decision Letter 2

**DOI:** 10.1371/journal.pone.0358751.r005

**Authors:** Vikas Sharma

25 Jun 2026

Dear Dr. Adejumobi,

Thank you for submitting your manuscript to PLOS ONE. After careful consideration, we feel that it has merit but does not fully meet PLOS ONE’s publication criteria as it currently stands. Therefore, we invite you to submit a revised version of the manuscript that addresses the points raised during the review process.

==============================

**ACADEMIC EDITOR:**  Please incorporate minor changes suggested by reviewers Aug 09 2026 11:59PM. If you will need more time than this to complete your revisions, please reply to this message or contact the journal office at plosone@plos.org. When you're ready to submit your revision, log on to https://www.editorialmanager.com/pone/ and select the 'Submissions Needing Revision' folder to locate your manuscript file.

- A letter that responds to each point raised by the academic editor and reviewer(s). You should upload this letter as a separate file labeled 'Response to Reviewers'.
- A marked-up copy of your manuscript that highlights changes made to the original version. You should upload this as a separate file labeled 'Revised Manuscript with Track Changes'.
- An unmarked version of your revised paper without tracked changes. You should upload this as a separate file labeled 'Manuscript'.

If you would like to make changes to your financial disclosure, please include your updated statement in your cover letter. Guidelines for resubmitting your figure files are available below the reviewer comments at the end of this letter.

If applicable, we recommend that you deposit your laboratory protocols in protocols.io to enhance the reproducibility of your results. Protocols.io assigns your protocol its own identifier (DOI) so that it can be cited independently in the future. For instructions see: https://journals.plos.org/plosone/s/submission-guidelines#loc-laboratory-protocols. Additionally, PLOS ONE offers an option for publishing peer-reviewed Lab Protocol articles, which describe protocols hosted on protocols.io. Read more information on sharing protocols at https://plos.org/protocols?utm_medium=editorial-email&utm_source=authorletters&utm_campaign=protocols.

As the corresponding author, your ORCID iD is verified in the submission system and will appear in the published article. PLOS supports the use of ORCID, and we encourage all coauthors to register for an ORCID iD and use it as well. Please encourage your coauthors to verify their ORCID iD within the submission system before final acceptance, as unverified ORCID iDs will not appear in the published article. *Only*  the individual author can complete the verification step; PLOS staff *cannot*  verify ORCID iDs on behalf of authors.

We look forward to receiving your revised manuscript.

Kind regards,

Vikas Sharma, Ph.D

Academic Editor

PLOS One

Journal Requirements:

If the reviewer comments include a recommendation to cite specific previously published works, please review and evaluate these publications to determine whether they are relevant and should be cited. There is no requirement to cite these works unless the editor has indicated otherwise.

Please review your reference list to ensure that it is complete and correct. If you have cited papers that have been retracted, please include the rationale for doing so in the manuscript text, or remove these references and replace them with relevant current references. Any changes to the reference list should be mentioned in the rebuttal letter that accompanies your revised manuscript. If you need to cite a retracted article, indicate the article’s retracted status in the References list and also include a citation and full reference for the retraction notice.

[Note: HTML markup is below. Please do not edit.]

Reviewers' comments:

Reviewer's Responses to Questions

**Comments to the Author**

Reviewer #3: (No Response)

Reviewer #4: All comments have been addressed

Reviewer #5: All comments have been addressed

Reviewer #6: All comments have been addressed

**********

2. Is the manuscript technically sound, and do the data support the conclusions?

Reviewer #3: Yes

Reviewer #4: Yes

Reviewer #5: Yes

Reviewer #6: Yes

**********

3. Has the statistical analysis been performed appropriately and rigorously?

Reviewer #3: Yes

Reviewer #4: Yes

Reviewer #5: Yes

Reviewer #6: Yes

**********

4. Have the authors made all data underlying the findings in their manuscript fully available?

The PLOS Data policy

Reviewer #3: Yes

Reviewer #4: Yes

Reviewer #5: Yes

Reviewer #6: Yes

**********

5. Is the manuscript presented in an intelligible fashion and written in standard English?

Reviewer #3: Yes

Reviewer #4: Yes

Reviewer #5: Yes

Reviewer #6: (No Response)

**********

Reviewer #3: The author should give information about the normal characteristics for this inbred lines. Also, author must give detailed information about degree of tolerance of such inbred lines thanks

Reviewer #4: The responses to the reviewers comments and the subsequent corrections are well received. I have no further comments or questions regarding the manuscript. Thank you.

Reviewer #5: Dear Authors,

Thank you for your careful and comprehensive responses to the previous round of reviews. Your revisions have significantly strengthened the manuscript, and I appreciate the additional analyses performed for the core set validation.

Before final publication, please address the following minor points:

1. Abstract correction: Revise the statement regarding the core set size from "15%" to "20%" to match the Methods, Results, and Table 6, where a 76-line (20%) core is clearly described.

2. Typographical fixes:

o Change "admixt" to "admixed" in the population structure methodology.

o Correct punctuation: "IITA. Ibadan" → "IITA, Ibadan."

3. Data Availability Statement: Please rephrase the statement to state clearly and directly that all SNP data underlying the findings are publicly available at the Figshare repository with the provided DOI, rather than using vague terms like "part of the data" or "extended data."

4. Verify completeness: Please confirm that Table 2 and Figure 1 are complete and correctly formatted in your final submission files, as they were not fully visible in the review PDF.

With these minor corrections, I believe your manuscript will be a valuable contribution to the field and suitable for publication in PLOS ONE.

Reviewer #6: all the comments have been suitably addressed. In my opinion the manuscript is suitable for publication now

**********

what does this mean?). If published, this will include your full peer review and any attached files.

If you choose “no”, your identity will remain anonymous but your review may still be made public.

**Do you want your identity to be public for this peer review?**  For information about this choice, including consent withdrawal, please see our Privacy Policy

Reviewer #3: No

Reviewer #4: **Yes:** Venkatesh Bollina

Reviewer #5: No

Reviewer #6: No

**********

[NOTE: If reviewer comments were submitted as an attachment file, they will be attached to this email and accessible via the submission site. Please log into your account, locate the manuscript record, and check for the action link "View Attachments". If this link does not appear, there are no attachment files.]

To ensure your figures meet our technical requirements, please review our figure guidelines: https://journals.plos.org/plosone/s/figures

You may also use PLOS’s free figure tool, NAAS, to help you prepare publication quality figures: https://journals.plos.org/plosone/s/figures#loc-tools-for-figure-preparation.

NAAS will assess whether your figures meet our technical requirements by comparing each figure against our figure specifications.

Attachment Submitted filename: Reviewer Report _SBulut.pdf

# Author comment: Author response to Decision Letter 3

**DOI:** 10.1371/journal.pone.0358751.r006

16 Jul 2026

Response to the Editor

We sincerely thank the Editor for the careful evaluation of our revised manuscript and for the encouraging comments. We greatly appreciate your positive assessment that the revisions have strengthened the manuscript and that the additional analyses performed for the core set validation have improved the study. We have carefully addressed all the remaining minor comments as outlined below.

Comment 1:

Abstract correction: Revise the statement regarding the core set size from "15%" to "20%" to match the Methods, Results, and Table 6, where a 76-line (20%) core is clearly described.

Response

Thank you for identifying this inconsistency. The Abstract has been revised to correctly state that the core collection comprised 76 lines (20%), ensuring consistency with the Methods, Results, and Table 6.

Comment 2:

Typographical fixes:

• Change "admixt" to "admixed" in the population structure methodology.

• Correct punctuation: "IITA. Ibadan" → "IITA, Ibadan."

Response

Thank you for pointing out these typographical errors. Both corrections have been made in the revised manuscript. Specifically, "admixt" has been corrected to "admixed," and the punctuation has been revised from "IITA. Ibadan" to "IITA, Ibadan."

Comment 3:

Data Availability Statement: Please rephrase the statement to state clearly and directly that all SNP data underlying the findings are publicly available at the Figshare repository with the provided DOI, rather than using vague terms like "part of the data" or "extended data."

Response

We appreciate this suggestion. The Data Availability Statement has been revised to clearly state that all SNP data underlying the findings of this study are publicly available in the Figshare repository through the provided DOI, in accordance with the journal's data availability requirements.

Comment 4:

Verify completeness: Please confirm that Table 2 and Figure 1 are complete and correctly formatted in your final submission files, as they were not fully visible in the review PDF.

Response

Thank you for bringing this to our attention. We have carefully verified the final submission files and confirm that Table 2 and Figure 1 are complete, correctly formatted, and fully visible in the revised manuscript submitted for publication.

Once again, we sincerely thank the Editor for the constructive comments and careful handling of our manuscript. We appreciate the opportunity to revise the manuscript and believe that the changes made have further improved its clarity, consistency, and overall quality. We hope that the revised version is now suitable for publication in PLOS ONE.

Attachment Submitted filename: Response to the Editor-final.docx

# Editor report: Decision Letter 3

**DOI:** 10.1371/journal.pone.0358751.r007

**Authors:** Vikas Sharma

10 Aug 2026

Dear Dr. Adejumobi,

Thank you for submitting your manuscript to PLOS ONE. After careful consideration, we feel that it has merit but does not fully meet PLOS ONE’s publication criteria as it currently stands. Therefore, we invite you to submit a revised version of the manuscript that addresses the points raised during the review process.

==============================

**ACADEMIC EDITOR:**

Please submit your revised manuscript by Sep 24 2026 11:59PM. If you will need more time than this to complete your revisions, please reply to this message or contact the journal office at plosone@plos.org. When you're ready to submit your revision, log on to https://www.editorialmanager.com/pone/ and select the 'Submissions Needing Revision' folder to locate your manuscript file.

- A letter that responds to each point raised by the academic editor and reviewer(s). You should upload this letter as a separate file labeled 'Response to Reviewers'.
- A marked-up copy of your manuscript that highlights changes made to the original version. You should upload this as a separate file labeled 'Revised Manuscript with Track Changes'.
- An unmarked version of your revised paper without tracked changes. You should upload this as a separate file labeled 'Manuscript'.

If you would like to make changes to your financial disclosure, please include your updated statement in your cover letter. Guidelines for resubmitting your figure files are available below the reviewer comments at the end of this letter.

If applicable, we recommend that you deposit your laboratory protocols in protocols.io to enhance the reproducibility of your results. Protocols.io assigns your protocol its own identifier (DOI) so that it can be cited independently in the future. For instructions see: https://journals.plos.org/plosone/s/submission-guidelines#loc-laboratory-protocols. Additionally, PLOS ONE offers an option for publishing peer-reviewed Lab Protocol articles, which describe protocols hosted on protocols.io. Read more information on sharing protocols at https://plos.org/protocols?utm_medium=editorial-email&utm_source=authorletters&utm_campaign=protocols.

As the corresponding author, your ORCID iD is verified in the submission system and will appear in the published article. PLOS supports the use of ORCID, and we encourage all coauthors to register for an ORCID iD and use it as well. Please encourage your coauthors to verify their ORCID iD within the submission system before final acceptance, as unverified ORCID iDs will not appear in the published article. *Only*  the individual author can complete the verification step; PLOS staff *cannot*  verify ORCID iDs on behalf of authors.

We look forward to receiving your revised manuscript.

Kind regards,

Vikas Sharma, Ph.D

Academic Editor

PLOS One

Journal Requirements:

If the reviewer comments include a recommendation to cite specific previously published works, please review and evaluate these publications to determine whether they are relevant and should be cited. There is no requirement to cite these works unless the editor has indicated otherwise.

Please review your reference list to ensure that it is complete and correct. If you have cited papers that have been retracted, please include the rationale for doing so in the manuscript text, or remove these references and replace them with relevant current references. Any changes to the reference list should be mentioned in the rebuttal letter that accompanies your revised manuscript. If you need to cite a retracted article, indicate the article’s retracted status in the References list and also include a citation and full reference for the retraction notice.

Additional Editor Comments:

Please check Space between [5,8] and [19,20]

Table 1, legend – "Geographic origin", please check and revise.

Burn in period – 20,000 not acceptable please do it with at least 100,000

MCMC – 20,000 not acceptable please do it with at least 100,000

whats is "Harvester"?

Table legends (bold)

Format references as per journals style

[Note: HTML markup is below. Please do not edit.]

To ensure your figures meet our technical requirements, please review our figure guidelines: https://journals.plos.org/plosone/s/figures

You may also use PLOS’s free figure tool, NAAS, to help you prepare publication quality figures: https://journals.plos.org/plosone/s/figures#loc-tools-for-figure-preparation.

NAAS will assess whether your figures meet our technical requirements by comparing each figure against our figure specifications.

# Author comment: Author response to Decision Letter 4

**DOI:** 10.1371/journal.pone.0358751.r008

19 Aug 2026

Response to the Editor

We sincerely thank the Editor for the careful review of our manuscript and for the constructive comments. We have carefully considered all the points raised and have made the corresponding revisions to improve the clarity, consistency, and methodological presentation of the manuscript. Our point-by-point responses are provided below.

Comment 1

Please check space between [5,8] and [19,20].

Response

Thank you for pointing this out. We carefully checked the in-text citations throughout the manuscript and corrected the spacing and citation formatting for [5,8] and [19,20] to ensure consistency with the journal's citation style.

Comment 2

Table 1, legend – "Geographic origin", please check and revise.

Response

Thank you for this observation. We reviewed the caption which included "Geographic origin" in Table 1 and removed this description because the relevant information was already adequately captured in-text where the germplasm is described as “tropical germplasm”. We therefore considered the information in the table caption redundant and removed it to improve the clarity and conciseness of Table 1.

Comment 3

Burn in period – 20,000 not acceptable; please do it with at least 100,000.

Response

We thank the Editor for this recommendation. The original population-structure analysis was performed using STRUCTURE with a burn-in period of 20,000 iterations. However, the original STRUCTURE/STRUCTURE HARVESTER analysis environment is no longer accessible to us, and therefore we were unable to rerun the original STRUCTURE analysis using the recommended 100,000-iteration burn-in period.

To address this concern and independently reassess the population structure, we reanalyzed the genotype dataset using the sparse non-negative matrix factorization (sNMF) algorithm implemented in the LEA package in R. The analysis evaluated K values from 1 to 10, with five independent repetitions for each K, and cross-entropy was used to evaluate the fit of the alternative K values. The resulting ancestry estimates and inferred population structure were consistent with the pattern obtained from the original analysis. The Materials and Methods section has therefore been revised to describe the LEA/sNMF analysis, and the corresponding population-structure results have been updated accordingly.

Comment 4

MCMC – 20,000 not acceptable; please do it with at least 100,000.

Response

We appreciate the Editor's recommendation. Because the original STRUCTURE analysis environment is no longer accessible, we did not report a new STRUCTURE analysis with 100,000 MCMC iterations without being able to reproduce and verify the original analysis. Instead, we performed an independent population-structure analysis using the sNMF algorithm implemented in the LEA package. The sNMF analysis was conducted for K = 1–10, with five independent repetitions per K, and cross-entropy was used to assess the alternative population-structure models. The resulting ancestry coefficients and population-structure pattern were highly consistent with those obtained from the original analysis. We have therefore replaced the original STRUCTURE-based description with the reproducible LEA/sNMF analysis in the revised manuscript.

Comment 5

What is "Harvester"?

Response

Thank you for raising this point. STRUCTURE HARVESTER is a post-processing tool used to summarize and visualize STRUCTURE results and facilitate the evaluation of alternative numbers of clusters (K). Because the revised population-structure analysis was performed using the sNMF algorithm implemented in the LEA package and no longer relies on STRUCTURE HARVESTER, we have removed the term "Harvester" from the revised manuscript and replaced the corresponding description with the LEA/sNMF methodology.

Comment 6

Table legends (bold).

Response

Thank you. We have reformatted all table titles/legends in accordance with the requested journal formatting and ensured consistency throughout the manuscript. We also checked the placement and formatting of table titles and legends. PLOS ONE specifies that table titles are placed above the tables, while legends and footnotes are placed below them.

Comment 7

Format references as per journal style.

Response

Thank you for this comment. We carefully checked and reformatted the entire reference list according to the PLOS ONE reference style. We also checked the in-text citation numbering and the formatting of author names, journal titles, volume and page/article information, and DOI details where applicable.

We appreciate the Editor's constructive comments and recommendations, which have helped us improve the methodological clarity, consistency, and presentation of the manuscript.

Attachment Submitted filename: Response to the Editor-4th round of review.docx

# Editor report: Decision Letter 4

**DOI:** 10.1371/journal.pone.0358751.r009

**Authors:** Vikas Sharma

7 Sep 2026

Genetic Diversity and Population Structure of Early-Maturing Yellow and Orange Maize Inbred Lines for Improved Breeding Applications

PONE-D-25-49435R4

Dear Dr. Adejumobi,

We’re pleased to inform you that your manuscript has been judged scientifically suitable for publication and will be formally accepted for publication once it meets all outstanding technical requirements.

Within one week, you’ll receive an e-mail detailing the required amendments. When these have been addressed, you’ll receive a formal acceptance letter and your manuscript will be scheduled for publication.

An invoice will be generated when your article is formally accepted. Please note, if your institution has a publishing partnership with PLOS and your article meets the relevant criteria, all or part of your publication costs will be covered. Please make sure your user information is up-to-date by logging into Editorial Manager at Editorial Manager® and clicking the ‘Update My Information' link at the top of the page. For questions related to billing, please contact billing support.

If your institution or institutions have a press office, please notify them about your upcoming paper to help maximize its impact. If they’ll be preparing press materials, please inform our press team as soon as possible -- no later than 48 hours after receiving the formal acceptance. Your manuscript will remain under strict press embargo until 2 pm Eastern Time on the date of publication. For more information, please contact onepress@plos.org.

Kind regards,

Vikas Sharma, Ph.D

Academic Editor

PLOS One

Additional Editor Comments (optional):

Some figure legends still written in bold and normal (Mixed style, please check that, it can be made uniform during publication stage/ Galley Proof).

Authors have addressed all the raised concerns and manuscript can be accepted now.

Reviewers' comments:

# Editor report: Acceptance letter

**DOI:** 10.1371/journal.pone.0358751.r010

**Authors:** Vikas Sharma

PONE-D-25-49435R4

PLOS One

Dear Dr. Adejumobi,

I'm pleased to inform you that your manuscript has been deemed suitable for publication in PLOS One. Congratulations! Your manuscript is now being handed over to our production team.

At this stage, our production department will prepare your paper for publication. This includes ensuring the following:

* All references, tables, and figures are properly cited

* All relevant supporting information is included in the manuscript submission,

* There are no issues that prevent the paper from being properly typeset

You will receive further instructions from the production team, including instructions on how to review your proof when it is ready. Please keep in mind that we are working through a large volume of accepted articles, so please give us a few days to review your paper and let you know the next and final steps.

Lastly, if your institution or institutions have a press office, please let them know about your upcoming paper now to help maximize its impact. If they'll be preparing press materials, please inform our press team within the next 48 hours. Your manuscript will remain under strict press embargo until 2 pm Eastern Time on the date of publication. For more information, please contact onepress@plos.org.

You will receive an invoice from PLOS for your publication fee after your manuscript has reached the completed accept phase. If you receive an email requesting payment before acceptance or for any other service, this may be a phishing scheme. Learn how to identify phishing emails and protect your accounts at https://explore.plos.org/phishing.

If we can help with anything else, please email us at customercare@plos.org.

Thank you for submitting your work to PLOS One and supporting open access.

Kind regards,

PLOS One Editorial Office Staff

on behalf of

Dr. Vikas Sharma

Academic Editor

PLOS One
